# The beginning and the end: flanking nucleotides induce a parallel G-quadruplex topology

**DOI:** 10.1093/nar/gkab681

**Published:** 2021-08-11

**Authors:** Jielin Chen, Mingpan Cheng, Gilmar F Salgado, Petr Stadlbauer, Xiaobo Zhang, Samir Amrane, Aurore Guédin, Fangni He, Jiří Šponer, Huangxian Ju, Jean-Louis Mergny, Jun Zhou

**Affiliations:** State Key Laboratory of Analytical Chemistry for Life Science, School of Chemistry & Chemical Engineering, Nanjing University, Nanjing210023, China; State Key Laboratory of Analytical Chemistry for Life Science, School of Chemistry & Chemical Engineering, Nanjing University, Nanjing210023, China; ARNA Laboratory, Université de Bordeaux, Inserm U1212, CNRS UMR5320, IECB, Pessac33607, France; ARNA Laboratory, Université de Bordeaux, Inserm U1212, CNRS UMR5320, IECB, Pessac33607, France; Institute of Biophysics of the Czech Academy of Sciences, Královopolská 135, 612 65 Brno, Czech Republic; Regional Centre of Advanced Technologies and Materials, Czech Advanced Technology and Research Institute (CATRIN), Palacky University Olomouc, Šlechtitelů 241/27,783 71, Olomouc – Holice, Czech Republic; State Key Laboratory of Analytical Chemistry for Life Science, School of Chemistry & Chemical Engineering, Nanjing University, Nanjing210023, China; ARNA Laboratory, Université de Bordeaux, Inserm U1212, CNRS UMR5320, IECB, Pessac33607, France; ARNA Laboratory, Université de Bordeaux, Inserm U1212, CNRS UMR5320, IECB, Pessac33607, France; State Key Laboratory of Analytical Chemistry for Life Science, School of Chemistry & Chemical Engineering, Nanjing University, Nanjing210023, China; Institute of Biophysics of the Czech Academy of Sciences, Královopolská 135, 612 65 Brno, Czech Republic; Regional Centre of Advanced Technologies and Materials, Czech Advanced Technology and Research Institute (CATRIN), Palacky University Olomouc, Šlechtitelů 241/27,783 71, Olomouc – Holice, Czech Republic; State Key Laboratory of Analytical Chemistry for Life Science, School of Chemistry & Chemical Engineering, Nanjing University, Nanjing210023, China; State Key Laboratory of Analytical Chemistry for Life Science, School of Chemistry & Chemical Engineering, Nanjing University, Nanjing210023, China; ARNA Laboratory, Université de Bordeaux, Inserm U1212, CNRS UMR5320, IECB, Pessac33607, France; Institute of Biophysics of the Czech Academy of Sciences, Královopolská 135, 612 65 Brno, Czech Republic; Laboratoire d’Optique et Biosciences, Ecole Polytechnique, CNRS, Inserm, Institut Polytechnique de Paris, 91128Palaiseau cedex, France; State Key Laboratory of Analytical Chemistry for Life Science, School of Chemistry & Chemical Engineering, Nanjing University, Nanjing210023, China

## Abstract

Genomic sequences susceptible to form G-quadruplexes (G4s) are always flanked by other nucleotides, but G4 formation *in vitro* is generally studied with short synthetic DNA or RNA oligonucleotides, for which bases adjacent to the G4 core are often omitted. Herein, we systematically studied the effects of flanking nucleotides on structural polymorphism of 371 different oligodeoxynucleotides that adopt intramolecular G4 structures. We found out that the addition of nucleotides favors the formation of a parallel fold, defined as the ‘*flanking effect*’ in this work. This ‘*flanking effect*’ was more pronounced when nucleotides were added at the 5′-end, and depended on loop arrangement. NMR experiments and molecular dynamics simulations revealed that flanking sequences at the 5′-end abolish a strong *syn*-specific hydrogen bond commonly found in non-parallel conformations, thus favoring a parallel topology. These analyses pave a new way for more accurate prediction of DNA G4 folding in a physiological context.

## Graphical Abstract

**Graphical Abstract:**
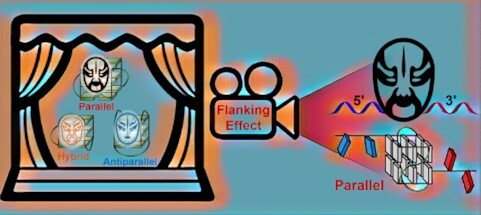
G-quadruplexes (G4) in the genomic context are flanked by other nucleotides. The addition of nucleotides favors the formation of a parallel fold, defined as the ‘flanking effect’, suggesting the biologically relevant G4 topology *in vivo* may often be parallel.

## INTRODUCTION

G-quadruplexes (G4s) are non-canonical nucleic acid structures formed by guanine-rich sequences (1,2), which are distributed in key regions of the genomes, such as telomeres and oncogene promoters (*e.g*. *c-kit*, *KRAS*, *c-myc*, *VEGF*). They contribute to essential cellular processes such as initiation of DNA replication, telomere maintenance and control of gene expression (3–5).

The topology of the G4 core may be parallel, antiparallel or hybrid, which provides a basis for the specific recognition by ligands and functional regulation, but also complicates structural and biophysical predictions (3,5). In addition, the motivation to explore new structures should be guided, at least in part, by evidence supporting their existence under physiological conditions. However, to simplify experimental design, studies have generally focused on short DNA (or RNA) oligonucleotides that match the core of the G-rich motif without considering its natural sequence context. Recently, efforts have been dedicated to the study of loop effects (6–8), but the role of flanking sequences has largely been ignored, even though the presence of flanking nucleotides is the rule rather than the exception when considering G4 formation within chromosomes.

Earlier reports point out that these extra nucleotides may play an important role: they are sometimes added to prevent higher order structures and favor intramolecular folding *in vitro* (9). In addition, flanking sequences may influence the formation of non-classical G4 structures (10), affect the terminal stacking between G4s (11,12), alter stability (13), and interact with the loops (14). A classic example of why flanking sequences are significant comes from the widely studied human telomeric motif (14,15), which can adopt diverse structures depending on the flanking nucleotides in K^+^ buffer (Supplementary Table S1). These observations suggest that flanking sequences alter the balance between topologies and favor specific conformations (16), although this has not been evaluated broadly or systematically.

Herein, 371 sequences were used to investigate the effect of flanking nucleotides on G4 topology. Strikingly, a general effect observed with over 80% of the tested motifs was discovered: The addition of flanking nucleotides favored a parallel topology over antiparallel or hybrid conformations. This flanking effect was observed under different ionic conditions and in sequences from the human and other genomes. The influence of flanking nucleotides was more pronounced at the 5′-end than at the 3′-end, and its strength depended on loop arrangement. Theoretical and experimental methods were combined to explain the intrinsic mechanism of the flanking effect. Our results demonstrate that flanking nucleotides should be considered when studying G4 structures and deepen our understanding of the folding process of G4 structures in a natural physiological context.

## MATERIALS AND METHODS

### Materials and reagents

#### DNA samples

DNA (except those used for NMR experiments) were purchased from Sangon Biotech. Sequence information is given in Supplementary Tables S2–S5.

#### Circular dichroism (CD)

CD spectra were recorded, with 5 μM DNA in 10 mM lithium cacodylate (pH 7.2) buffer supplemented with 100 mM KCl (or NaCl), to distinguish G4 topologies. Topologies were identified using the conformation index *r* (Equation 1) (6,17):

(1) $$\begin{equation*}{r} = \frac{{{\rm{C}}{{\rm{D}}_{{\rm{265}}}}}}{{{\rm{|C}}{{\rm{D}}_{{\rm{265}}}}{\rm{| + C}}{{\rm{D}}_{{\rm{290}}}}}}\end{equation*}$$

where CD_265_ and CD_290_ are the CD ellipticities at 265 and 290 nm, respectively. *r* ≥ 0.5, 0 ≤ *r* < 0.5, and *r* < 0 correspond to predominantly parallel, hybrid, and antiparallel topologies, respectively (6).

#### UV-melting experiments and UV absorbance spectroscopy

Melting temperature (*T*_m_) was determined by analysis of the first derivative of the melting curve. The isothermal difference spectrum (IDS) and thermal difference spectrum (TDS) was collected as described previously, which provide specific signatures for G4 formation (18,19).

#### Nuclear magnetic resonance (NMR)

HPLC purified oligonucleotides, purchased from IDT, further filtrated through 2-kDa molecular-weight cut-off filters were used for NMR experiments (The synthesis procedure of isotopically enriched samples are detailed in Supporting Information). NMR samples included 10% (v/v) D_2_O. NMR spectra were collected at 25°C with several classical experiments, including 1D ^1^H–^15^N/^13^C HMQC, 2D ^1^H–^1^H NOESY, TOCSY and ^1^H–^13^C HMBC (20).

#### Molecular dynamics (MD) simulations

Ten three-quartet G4 models differing in *anti*-*syn* combination of guanines and presence of flanking thymines were built up from known structures (PDB ID’s: 3TVB (21), 2GKU (22), 143D (23)). These were subjected to 2.5 μs long explicit solvent MD simulations each. Relative free-energy differences between the models were estimated by the MM-PBSA method (24) according to a previously applied protocol (25). Modeling and calculations were done with the AMBER program package (26) using the OL15 force field (27). See Supporting Information for more details.

## RESULTS

### Sequence design and nomenclature

To understand the effect of flanking nucleotides on G4 topology, a set of 150 model sequences (Supplementary Table S2) and 40 natural sequences, collected from the literature or identified by BLAST search of human and other genomes (28,29), were investigated first (Supplementary Tables S3–S5). To minimize the interaction between nucleotides from loops and flanking regions, both were mainly composed of thymines in model sequences unless otherwise stated. These sequences are represented as 5′-GGG*Ta*GGG*Tb*GGG*Tc*GGG-3′, where *a*, *b* and *c* are three integers corresponding to the number of nucleotides in the first (5′), second (central), and third (3′) loops, respectively, with a total loop length (*a* + *b* + *c*) of 7–13 nucleotides. When the sequences contained two extra thymines at the 5′-end, 3′-end, or both ends, they were named 5'T2-*abc*, 3'T2-*abc* or DT2-*abc*, respectively. For example, DT2-136 means that two thymines were added to both ends of a sequence with the first, second, and third loops of one, three, and six thymines, respectively. Further, a *group* of sequences is defined as all sequences formed by loop swapping; for instance, the ***136*** group is composed of six oligonucleotides with any combination of loops with one, three, and six thymines, which gives sequences with *a*, *b*, *c* values of 136, 163, 316, 361, 613 and 631; the group names are written in bold, italicized font.

### Influence of flanking sequences on topology

G4 formation was evaluated using a combination of biophysical experiments. CD spectra indicated that all sequences form G4 structures in the presence of 100 mM K^+^ (Supplementary Figure S1) or 100 mM Na^+^ (Supplementary Figure S2), although topologies differed, as discussed below. Thermal difference spectra (TDS, Supplementary Figure S3), isothermal difference spectra (IDS, Supplementary Figure S4), and 1H NMR spectra (Supplementary Figure S5) of three representative groups (***136***, ***144*** and ***145***) further verified the formation of G4 (18,19). In addition, formation of intramolecular structures was confirmed by size-exclusion chromatography (30) (Supplementary Figure S6).

**Figure 1.**
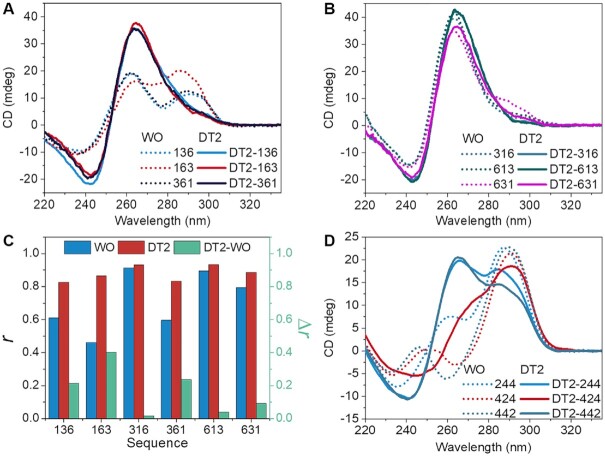
Influence of flanking sequences on topology. (**A**,**B**) CD spectra of ***136*** group oligonucleotides without (WO) or with (DT2) two thymines at both ends. (**C**) Values of the conformation index *r* for the ***136*** (blue) and DT2-***136*** (red) groups. The difference (Δ*r* = *r*_DT2_ – *r*_WO_) is shown in green (right Y-axis). *r* values for all other sequences are given in Supplementary Table S2. (**D**) CD spectra of the three sequences belonging to the ***244*** group, without (WO) or with (DT2) two thymines at both ends. Dashed lines indicate the parent sequences with no flanking nucleotides and solid lines indicate the corresponding DT2 sequences. All experiments were performed in 100 mM KCl.

To distinguish and to quantify the conformational differences of G4s reflected by the CD spectra, the conformation index *r* was calculated (Equation 1 shown in experimental procedures) as previously described (6,17). The G4 conformations can be divided into three types based on *r* values: parallel (*r* ≥ 0.5), hybrid (0 ≤ *r* < 0.5), and antiparallel (*r* < 0) (6,17). Results for all sequences are shown in Supplementary Figures S7–S8 and Table S2. In the ***136*** group, three sequences (136, 163 and 361) had shoulder peaks around 290 nm (Figure 1A), indicating that a significant proportion of the population was non-parallel structures. For the three other sequences (316, 613 and 631), CD indicated the predominance of the parallel fold (Figure 1B). When pairs of thymines were added to both ends of each sequence (DT2-***136*** group), the shoulders at 290 nm disappeared for DT2-136, DT2-163 and DT2-361, and the peaks at 265 nm were strengthened (Figure 1A). These results suggest that a conformational switch from non-parallel to parallel ensemble was induced by flanking nucleotides. This was verified by 2D ^1^H–^1^H NOESY NMR for two model sequences (163 and DT2-163) as detailed below. We refer to this phenomenon as the ‘flanking effect’. The other three combinations (316, 613 and 631), which were mostly or exclusively parallel in absence of flanking sequences, were not strongly affected by flanking nucleotides (Figure 1B). Nevertheless, comparison of these six sequences revealed that they exhibited higher *r* values (Δ*r = r*_DT2_ – *r*_WO_, Δ*r* > 0) upon the addition of two flanking thymines at both ends (Figure 1C). Similar changes in CD spectra were observed in the ***244*** group upon addition of flanking thymines (Figure 1D).

**Figure 2.**
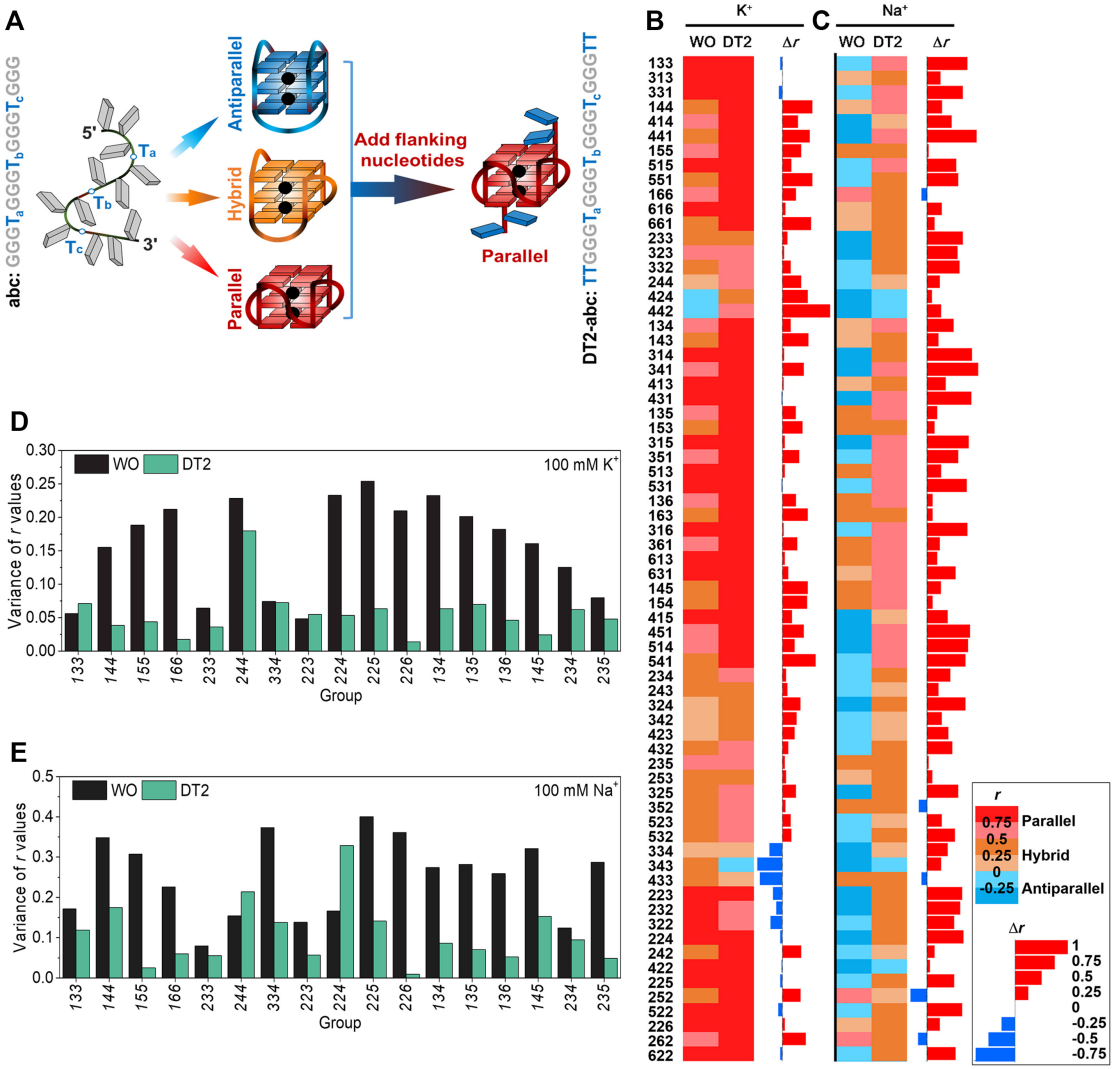
Flanking effect on G4 topology with *r* values analysis. (**A**) Schematic illustration of the flanking effect. (**B**, **C**) *r* values of sequences with (DT2) or without (WO) two flanking thymines at both ends and their differences (Δ*r = r*_DT2_ - *r*_WO_) in (B) 100 mM KCl and (C) 100 mM NaCl. The values of the conformation index *r* are shown as heat maps: parallel (*r* ≥ 0.5, red), hybrid (0 ≤ *r* < 0.5, khaki), and antiparallel (*r* < 0, blue). (**D**, **E**) The variance of *r* values (${\rm{\sigma \; = \;}}\sqrt {\frac{{\sum {{{\rm{(}}{r_i}{\rm{\; - \;\mu )}}}^{\rm{2}}}}}{{\rm{N}}}}$) for each group in (D) 100 mM KCl and (E) 100 mM NaCl.

CD spectra and the *r* values for all model sequences were collected and determined (Supplementary Figures S1–S2 and Table S2). The *r* values were almost always higher after the addition of two thymines at both ends (Figure 2A), suggesting that flanking nucleotides result in a preference for the parallel topology. For example, most sequences adopted a hybrid or parallel fold without flanking nucleotides in K^+^ buffer, and addition of thymines to both ends usually resulted in an increase in the parallel population (Figure 2B). Adding these flanking sequences not only converted hybrid structures to parallel but also induced an antiparallel to hybrid or parallel conversion as observed for 424 and 442 (Figure 1D), with *r* values changed from –0.13 and –0.16 to 0.27 and 0.60, respectively (Supplementary Table S2). The same CD experiments were performed in Na^+^ buffer as many G4-forming sequences tend to be parallel in K^+^ buffer and non-parallel in Na^+^ buffer (28). In Na^+^ buffer, more sequences adopted an antiparallel fold without flanking sequences, and the presence of thymines on both 5′- and 3′- ends resulted in an increase in *r* values for all but five of the sixty-nine sequences evaluated (Figure 2C). These examples demonstrate that the presence of flanking nucleotides favors a parallel topology with very few exceptions.

The average *r* value of each group generally increased upon the addition of flanking nucleotides, especially in Na^+^ buffer (Supplementary Table S6 and Figure S7). Moreover, after adding the flanking nucleotides, the *r* value variance for every group decreased significantly (Figures 2D and E), indicating that the sequences within the same group tend to adopt similar topologies (which we assume reflects the overall parallelization), with a few exceptions such as the ***244*** and ***224*** groups in Na^+^ buffer (Figure 2E). In other words, the presence of flanking nucleotides affects the balance between G4 conformations and favors a predominantly parallel conformation.

### Flanking effect in natural G-rich motifs

To complement the systematic study of model sequences, we selected eight natural (28) and six previously studied G4 sequences (29) (Supplementary Table S3) to determine whether the flanking effect is observed. Indeed, the addition of two thymines at both 5′- and 3′-ends resulted in increases in *r* values for 12 of the 14 sequences both in K^+^ (Supplementary Figure S8) and Na^+^ (Supplementary Figure S9). To analyze non-thymine flanking sequences, 32 natural sequences were selected by BLAST search (Supplementary Tables S4 and S5). Again, the flanking effect was observed in K^+^ (Supplementary Figure S10) and in Na^+^ (Supplementary Figure S11): For all the sequences, the population with a parallel fold increased when flanking nucleotides were included.

### Asymmetry of the flanking effect

**Figure 3.**
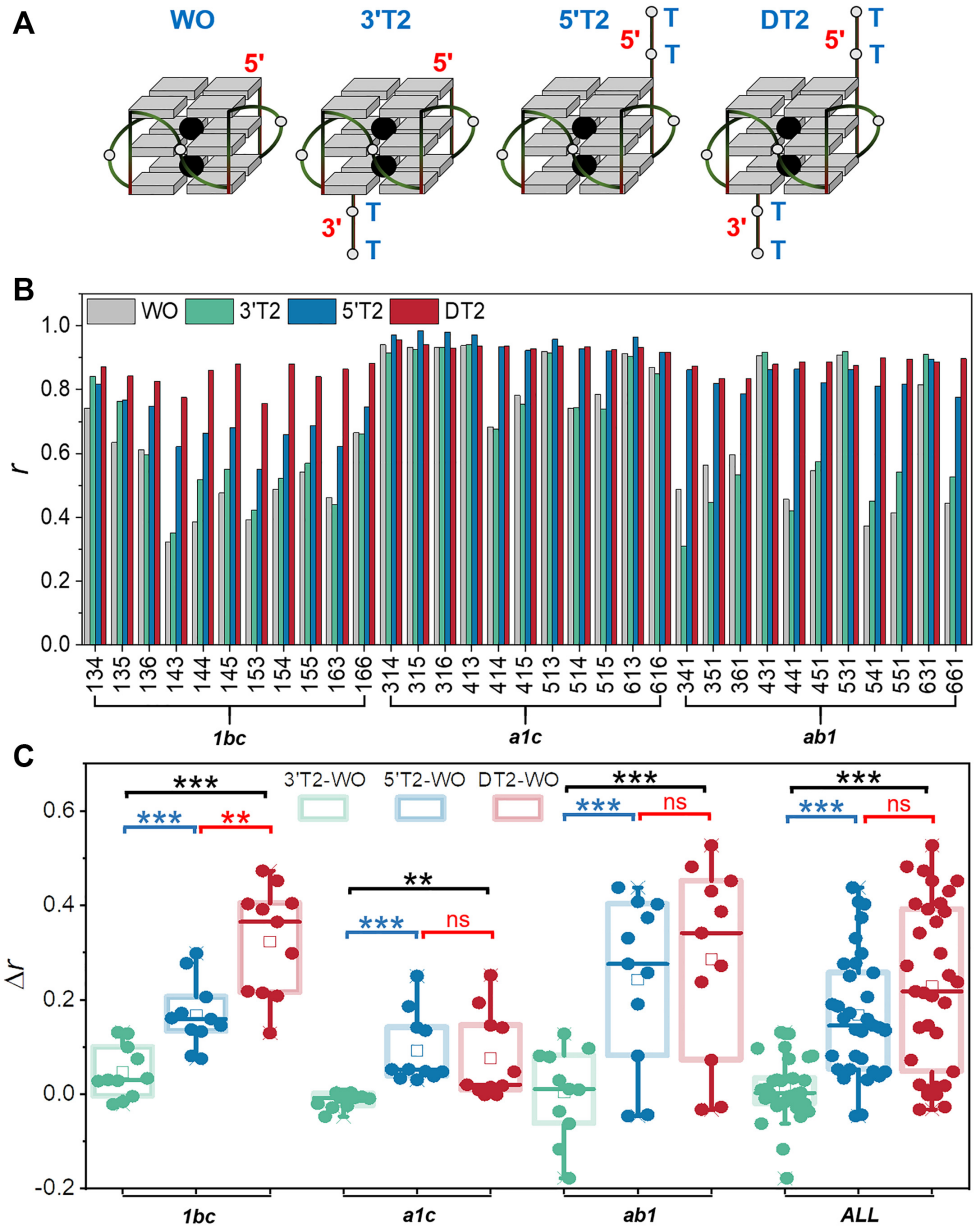
Asymmetry of the flanking effect. (**A**) Schematic illustrations of flanking nucleotide addition at different ends. (**B**) The *r* values for ***134***, ***135***, ***136***, ***144***, ***145***, ***155*** and ***166*** groups without any flanking nucleotide (WO) and with thymines added at the 3′- (3'T2), 5′- (5'T2) or both ends (DT2) in 100 mM K^+^. (**C**) Paired *t*-test of differences in *r* values difference (Δ*r = r*_3'T2,__or__5'T2 or DT2_ – *r*_WO_) between sequences with and without thymines for 3'T2, 5'T2 and DT2 groups. ***P < 0.001; **P < 0.01; ns: *P* > 0.05, no significant difference.

To understand the relative contributions of the 5′ and 3′ flanking nucleotides, we uncoupled these two modifications by studying the impact of the addition of two thymines at only the 5′-end or only the 3′-end (Figure 3A). We investigated representative sequences from the ***134***, ***135***, ***136***, ***144***, ***145***, ***155*** and ***166*** groups with two thymines added at the 3′-end (3'T2) or 5′-end (5'T2), and compared them to both ends (DT2). The flanking effect was far more pronounced at the 5′-end than at the 3′-end (Figure 3B and Supplementary Figures S12 and S13). In most cases, the 3′-end additions had little or no impact on topology, and sometimes led to opposite effects (Δ*r* < 0, *e.g*. 341 and 351), whereas a shift to parallel conformation was observed in the majority of cases when two thymines were added to the 5′-end.

We subdivided the sequences with one of the loops composed of a single thymine into three categories: (i) The shortest loop is the one closest to 5′-end (*e.g*. 134, 135, 136, etc.; ***1bc*** in Figure 3B); (ii) the central loop is the shortest (*e.g*. 314, 315, 316, *etc*.; ***a1c*** in Figure 3B) and (iii) the third loop is the shortest (*e.g*. 341, 351, 361, etc.; ***ab1*** in Figure 3B). We found that the location of the shortest loop influenced the effects of flanking nucleotides:

- When the shortest loop is in the middle position (*i.e*.***a1c***), the *r* values of sequences without terminal thymines are similar to those of the DT2 group (314, 315, 316, 413, 513, 613 and 616). Thus, sequences with a short central loop prefer to be parallel *per se*, whereas the other two groups (***1bc*** and ***ab1***) show less parallel or rather hybrid topologies. The sequences with a short central loop which are not fully parallel (414, 415, 514 and 515) in the absence of flanking thymines are fully converted to a parallel topology (*r* values are close to 1) upon addition of thymines at 5′-end.
- When the third loop is the shortest one (***ab1***), the *r* values of sequences without terminal thymines are near 0.5, except for 431 (0.91), 531 (0.91) and 631 (0.81). For these sequences, the addition of two thymines at the 5′-end increased *r* values from 0.5 to near 0.8, while for the DT2 group, *r* values reached 0.9.
- Finally, when the first loop was the shortest one (***1bc***), most *r* values for the 5'T2 group increased to around 0.65, except for 134, 135, 136 and 166, which exhibited higher *r* values with a 5'T2 flank. However, the CD data indicate that these sequences have an obvious shoulder peak around 290 nm, even with 5′-end flanking nucleotides, which means that a fraction of the population remains non-parallel (Supplementary Figure S12). These results demonstrate that the 5′-end flanking nucleotides have a weaker parallelization effect on sequences in which the first loop is the shortest one.

Globally speaking, the flanking effect is stronger at the 5′-end than at the 3′-end in K^+^ buffer, which is also verified by the results in Na^+^ buffer (Supplementary Figures S14 and S15). The 5′-end flanking effect also depends on the position of the shortest loop (Figure 3C). When the shortest loop is the second (***a1c***) or third (***ab1***) one, nucleotides added to the 5′-end can make the sequences completely parallel, and these effects are comparable to the flanking effects of two thymines at both ends. However, when the first loop is the shortest (***1bc***), the effect of 5'T2 is less pronounced than the effect of thymines added at both ends.

### Dependence of the flanking effect on the length of the 5′-terminal overhang

**Figure 4.**
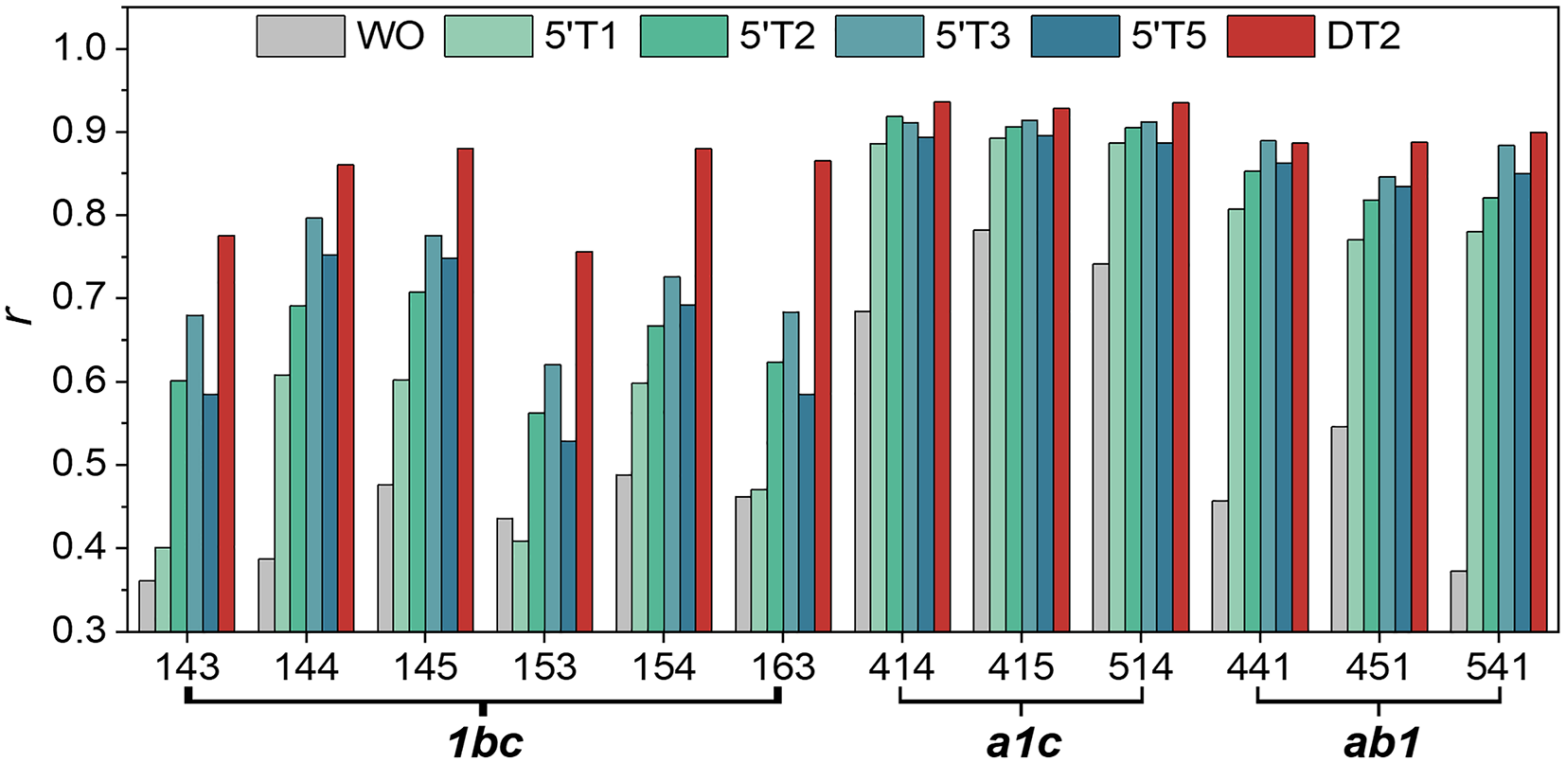
Length dependence of the flanking effect at the 5′-end. *r* values are provided for sequences with two thymines added at both ends (DT2) and 0, 1, 2, 3 or 5 thymines added at the 5′-end only (WO, 5'T1, 5'T2, 5'T2, 5'T3 and 5'T5). Experiments were performed in 100 mM KCl.

To evaluate whether the length of the 5′ overhang is important, we compared the impact of 5′-terminal dangling ends varying from 0 to 5 thymines (Supplementary Figure S16). When the *first* loop is the shortest (*i.e*. 143, 153, 163, 144, 145 and 154), adding a single 5′-thymine (5'T1) had little (*i.e*. 144, 145 and 154) or no effect (*i.e*. 143, 153 and 163) on *r* values. Increasing the 5'-overhang length further increased *r* values but even a 5-nucleotides overhang (5'T5) did not transform the topology to the same level as DT2 group (Figure 4). When the *middle* loop is the shortest (*i.e*. 414, 415 and 514), the structures were predominately parallel even in the absence of flanking nucleotides (Figure 4). The addition of 5′-end flanking sequences further increased the *r* values. Interestingly, a single thymine on the 5′-end (5'T1) had nearly the same effect as two on both ends, and further lengthening of the 5′-overhang had a little effect. When the shortest loop is the *third* loop (*i.e*. 441, 451 and 541), the addition of one or more 5′-thymines increased *r* values, but differences were relatively modest. In other words, a single 5′-thymine accounted for most of the *r* values increase. These data show that the flanking effect may depend on the length of 5′ overhang but that most of the flanking effect is due to the nucleotide immediately adjacent to the G4 core on the 5′ side.

### Statistical analysis of the flanking effect

**Figure 5.**
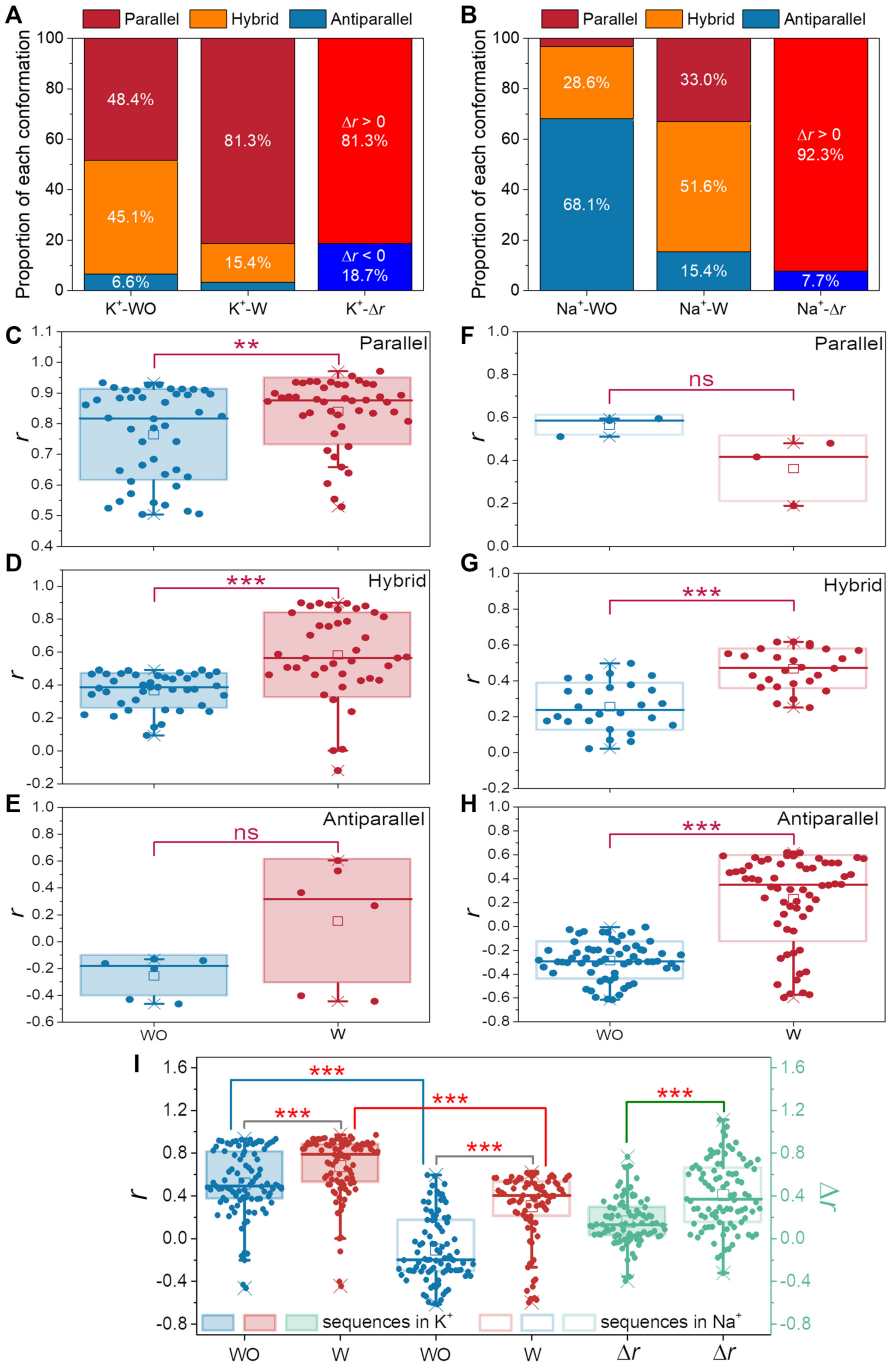
Statistical analysis of the flanking effect. (**A, B**) Proportion of different conformations without (WO) or with (W) flanking nucleotides, and their *r* value differences (Δ*r*) in **A**) K^+^ and **B**) Na^+^ buffer. (**C-I**) Paired *t*-tests of *r* values with (W) or without (WO) flanking nucleotides added at both ends and separated into three groups based on their primary topologies in (**C*–*E**) 100 mM K^+^ or (**F*–*H**) 100 mM Na^+^. For panels (**C**) and (**F**), the primary topology was parallel, *r* ≥ 0.5; for panels (**D**) and (**G**), the primary topology was hybrid, 0 < *r* < 0.5; and in panels (**E**) and (**H**), the primary topology was antiparallel, *r* < 0. *P*-values are provided for the comparison of flanking versus no-flanking sequences; ***P < 0.001; **P < 0.01; ns: *P* > 0.05, no significant difference. Both natural and model sequences listed in Supplementary Tables S2–S5 are considered here.

The differences in *r* values between sequences with or without flanking nucleotides are summarized in Figures 5A and B, Supplementary Figure S17 and Tables S2–S5. Strikingly, in K^+^ buffer (Figure 5A), before the addition of flanking nucleotides, both parallel and hybrid forms are the predominant conformations, and addition of flanking nucleotides converts most of the sequences (81.3%) into parallel structures. In Na^+^ buffer (Figure 5B), the addition of flanking nucleotides leads to a decrease in the proportion of antiparallel folds (from 68.1% to 15.4%), with a concomitant increase in parallel (from 3.3% to 33.0%) and hybrid (from 28.6% to 51.6%) configurations. The Δ*r* values were positive in 81.3% of all cases (74 out of 91) in K^+^, and even 92.3% (84 out of 91) in Na^+^.

The sequences were then grouped by their intrinsic topologies in the absence of any flanking nucleotides: parallel, hybrid, or antiparallel. Comparison of these *r* values are provided in Figures 5C–H. Very significant statistical differences (*P* < 0.001) were found between hybrid G4s in both K^+^ (Figure 5D) and Na^+^ (Figure 5G), as well as antiparallel G4s in Na^+^ (Figure 5H), whereas a significant difference (*P* = 0.0077) was found for parallel G4s in K^+^ (Figure 5C). In contrast, no significant differences (*P* > 0.05) were found for antiparallel G4s in K^+^ (Figure 5E) or for parallel G4s in Na^+^ (Figure 5F), possibly as a result of smaller sample sizes.

Further, very significant differences were found between sequences with and without flanking nucleotides both in K^+^ (*P* = 3.57E–04) and Na^+^ (*P* = 2.55E–15) (Figure 5I). Concurrently, the differences between K^+^ and Na^+^ for sequences with (*P* = 1.19E–13) and without (*P* = 1.35E–25) flanking nucleotides were also highly significant, which illustrates that most sequences adopt a parallel topology in K^+^. Moreover, the Δ*r* values in Na^+^ are larger than those in K^+^. This is consistent with previous observations that the fraction of sequences exhibiting a positive Δ*r* is higher in Na^+^ (92.3%) than in K^+^ (81.3%) (Figure 5A). These results demonstrate that the switch from non-parallel to more parallel (*e.g*. antiparallel to hybrid, or hybrid to parallel) is easier and larger in magnitude in Na^+^ than in K^+^.

### Effect of flanking sequences on thermal stability

We determined the melting temperatures (*T*_m_) in K^+^ buffer of all model sequences (Supplementary Table S7 and Figure S18) and discovered that the presence of flanking nucleotides tends to *destabilize* the G4 structure. Nearly all the *T*_m_ values were lower when flanking nucleotides were present, sometimes dramatically, as found for sequence 253 for which *T*_m_ decreased 16.5°C. In general, this effect is relatively modest, however (Δ*T*_m_ < 4°C for 45 out of 69 sequences). Moreover, we found little or no correlation between *r* and *T*_m_ values with or without flanking nucleotides (*R*^2^ = –0.009 and 0.06, respectively, Supplementary Figure S19): this implies that the topology does not directly determine the thermal stability of the G4 structure.

**Figure 6.**
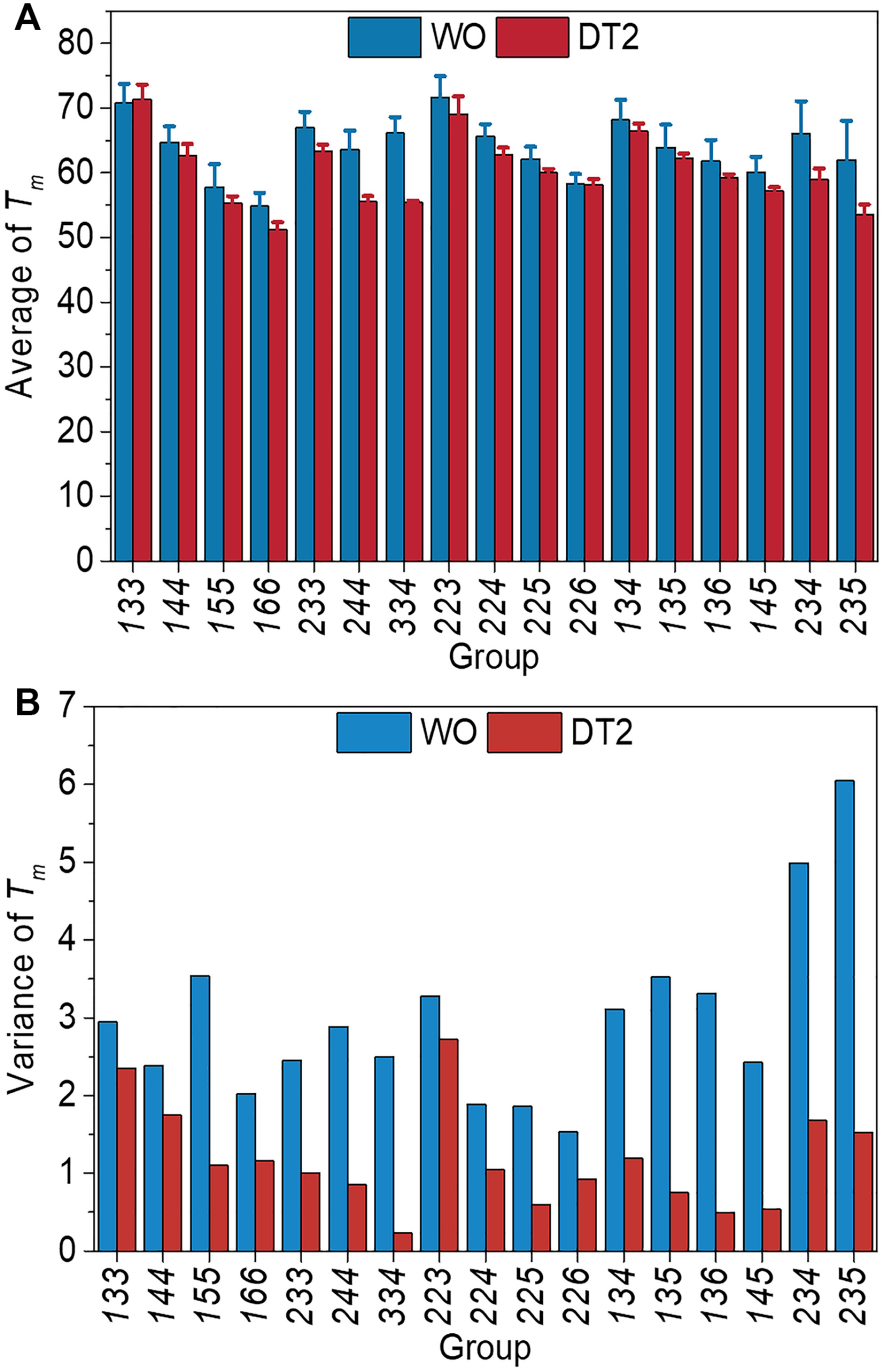
Influence of the flanking nucleotides on G4 thermal stability. (**A**) Average *T*_m_ values without (WO) or with (DT2) two thymines at both ends for every group. (**B**) The variance of *T*_m_ values (${\rm{\sigma \; = \;}}\sqrt {\frac{{\sum {{{\rm{(}}{T_{m,i}}{\rm{\; - \;\mu )}}}^{\rm{2}}}}}{{\rm{N}}}}$) for each group in 100 mM KCl.

Calculations of average *T*_m_ values for each group revealed that the flanking nucleotides have a rather small impact on the average value of the entire group (the larger differences were observed for ***244**, **334**, **234*** and ***235*** groups) (Figure 6A). Moreover, variations in *T*_m_s within groups were significantly smaller when flanking nucleotides were present than when they were not (Figure 6B). This is consistent with the smaller variance of the *r* value observed when there were flanking nucleotides (Figure 2D-E). In other words, when flanking sequences are present, the sequences in the same group tend to adopt similar topologies with similar stabilities.

### MD simulations suggest the potential mechanism of the 5′-terminal flanking effect

Our previous research has suggested that a 5′-terminal G with a *syn* glycosidic bond orientation can form a stabilizing 5′O-H···N3 hydrogen bond, while a 5′-terminal *anti*-G cannot (31,32). To further evaluate the effect of 5′-terminal *syn* Gs, we performed MD simulations followed by MM-PBSA free-energy calculations and hydrogen-bond population analysis. We considered the following four model systems: parallel all-*anti*, parallel with the first G-quartet converted to *syn*, and a hybrid and an antiparallel conformation with known *syn/anti* patterns (Supplementary Figures S20–S22). The simulations were carried out with or without a single thymine as the 5′-end flanking nucleotide. The MM-PBSA free-energy analysis allowed comparison of relative energies of the stems with or without the flanking sequences. The free-energy calculations were done in such a way that the intrinsic stability of the G4 stem structure was evaluated with effects of the flanking segments subtracted (see Supporting Information).

**Figure 7.**
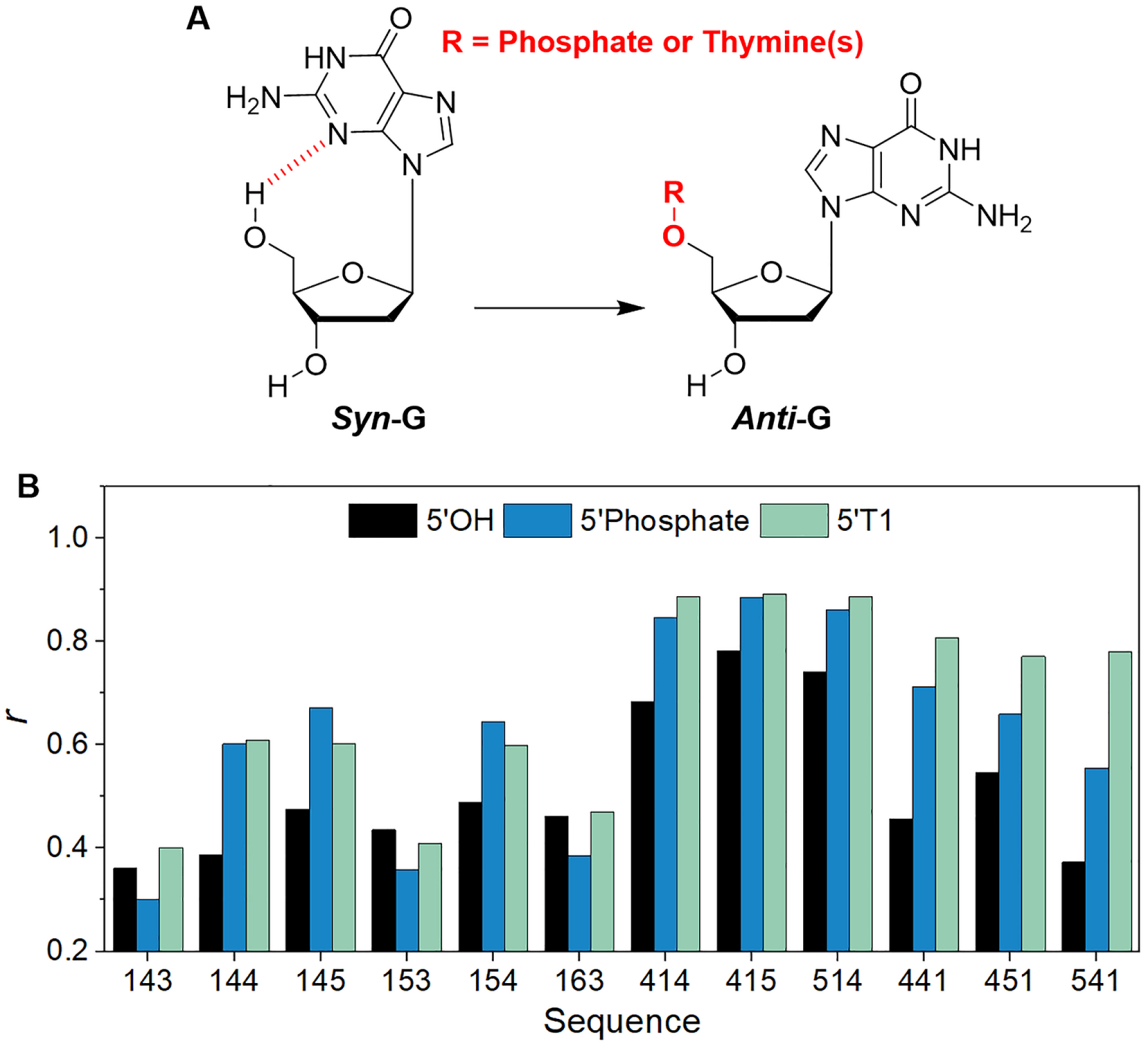
Influence of the glycosidic bond on G4 conformation. (**A**) In the *syn* G conformation, a hydrogen bond (5′O-H···N3) can be formed provided that the 5′ OH group is available *(*i.e. when this G is the 5′-terminal base and there is no terminal phosphate). (**B**) *r* values for sequences with 5′ hydroxyl (5'OH), 5′ phosphate (5′Phosphate) and a single 5′ thymine (5'T1) in 100 mM KCl.

The calculations confirmed that the presence of a 5′-end *syn* G is accompanied by the formation of a stable terminal intramolecular 5′O-H···N3 hydrogen bond (Figure 7A). No terminal hydrogen bond can be formed when the 5′-end G is in *anti*-conformation, as there is no suitable acceptor. The terminal hydrogen bond is also eliminated by the presence of a 5′-end flanking nucleotide. The reason is that the interaction requires that the G has a free 5′-OH end, which is obviously impossible when a 5′-end flanking sequence is present, irrespective of its length or nucleotide composition.

Any experimentally studied G4 sequence with a 5′-terminal G may form a mixture of G4 structures. Many have the 5′-terminal G in the *syn* conformation, whereas in the parallel G4 the conformation is *anti*. Any 5′-end flanking nucleotide abolishes the formation of the 5′O-H···N3 hydrogen bond in all the structures with a 5′-end *syn* G but has no effect on folds with this G in the *anti* conformation (Supplementary Tables S8–S10). Thus, in the ensembles of measured structures, there is a universal 5′-end flanking effect that destabilizes all folds that have a 5′-terminal *syn* G with respect to all the remaining folds. Therefore, we suggest that this destabilization of conformations with a 5′-terminal *syn* G explains in large part the increased population of parallel-stranded structures upon addition of 5′-end flanking sequences (additional analyses and discussion of the free-energy estimate and justification are available in Supplementary data). The experimental results agree with the picture suggested by the simulations: Most of the *r* values for sequences with 5′-phosphate are similar to those of sequences with a single 5′ thymine (Figure 7B). Further, the flanking effect is not affected by the identity of flanking nucleotide (Supplementary Figure S23). All the data imply that the dominant mechanism for the 5′-flanking effect is the elimination of the hydrogen bond between 5′-OH and 5′-terminal *syn* G.

We verified that the abolished H-bond is not replaced by the possible *syn*-specific interaction between the amino group and phosphate group of the G (see Supplementary Tables S11-S12 and the accompanying Supplementary data discussion).

### NMR analysis supports the mechanism of the flanking effect

**Figure 8.**
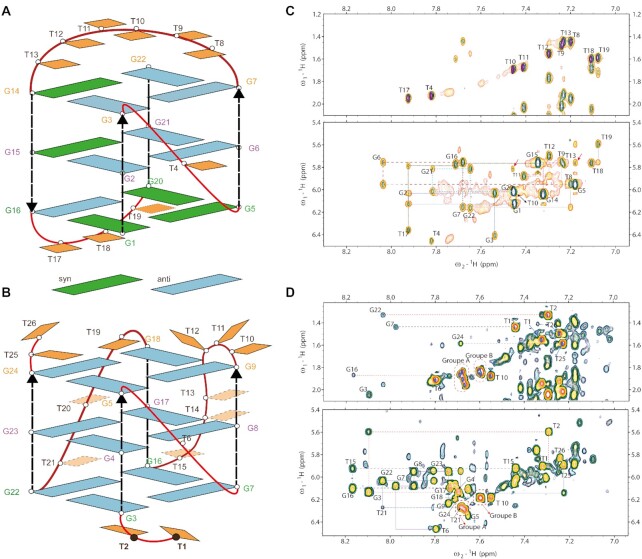
(**A**) 163 adopts a hybrid conformation, having five guanines with *syn* glycosidic bond angles (G1, G5, G14, G15 and G20; shown in green), including the 5′-terminal G. (**B**) DT2-163 adopts a parallel conformation, where all guanines adopt an *anti* glycosidic bond (cyan). (**C**, **D**) 2D ^1^H–^1^H NOESY NMR spectra of oligo 163 and DT2-163 respectively in 10 mM KPi buffer, acquired at 25°C and obtained with a mixing time of 250 ms. The top (ω_1_ = 1.2–2.1 ppm) and bottom (ω_1_ = 5.4–6.5 ppm) spectrum depicts NOEs that correlate the CH3 (purple and blue) from thymines and H8/H6H1 base respectively, with H1’ sugar, representing the sequential connectivities with dashed lines. The red arrows in panel C indicate the ‘square’ pattern of G*_syn_*–G*_anti_* observed in conformer 163.

NMR spectroscopy was used to analyze the interaction between G-quartets and flanking nucleotides in atomic detail. The fold topology of 163 and DT2-163, were analyzed and compared, with additional information gained from the analysis of 5'T2-163 and 3'T2-163 (Figure 8). To unambiguously study the cross peaks in 2D ^1^H–^1^H NOESY spectra, we performed the assignments for all guanines using selectively labeled samples containing a single guanine enriched with both ^15^N and ^13^C isotopes (Supplementary Figure S24). Natural abundance long-range ^1^H–^13^C HMBC (Supplementary Figure S25) was used to identify the H8 hydrogens belonging to each guanine. The assignments were also supported by 2D ^1^H–^1^H TOSCY and ^1^H–^13^C HSQC and by analyses of samples containing point substitutions that replaced thymines with 2′-deoxyuridines. The results clearly show that both 163 and DT2-163 exhibit 12 sharp imino peaks. In 2D ^1^H–^1^H NOESY spectra, we observed G(i)H8/G(i + 1)H1' NOEs for both 163 and DT2-163 that are characteristic of hybrid and parallel conformations respectively (Figures 8C and D). Most importantly, the intensities of the GH8-H1′ cross peaks in the 163 spectrum indicate that five Gs adopt a *syn* conformation (G1, G5, G14, G15 and G20), but no *syn* Gs were observed in DT2-163 (Figures 8A and B). This observation corroborates the prediction obtained from MD simulations that the absence of a terminal intramolecular 5′O-H···N3 hydrogen bond results in a loss of the (substantial) stabilization of the *syn* conformation and the formation of a more stable parallel fold.

The spectra revealed that additional interactions are present, because the terminal or/and loop thymines can interact with the 5′-end G-quartet and stabilize the parallel conformation. The thymines in the lateral loop T17–T19 of sequence 163 have multiple contacts with the 5′-end G-quartet and stabilize the hybrid topology, which is indicated by intense cross peaks with the guanines in the 5′-end G-quartet. In contrast, for DT2-163, the methyl group of T2 is roughly at the same distance from H1 of G16 and G22 (≈3–4.5 Å) and its H6 is closer to H1 of G22, meaning that T2 can stack and interact with the 5′-terminal G-quartet, as well as T1 and T15 to a lesser extent.

The NMR data for 5'T2-163 and 3'T2-163 also support for the hypothesis that the 5′-terminal flanking nucleotide influences the conformation of the adjacent guanine (Figure 8, and Supplementary Figure S26). 5'T2-163 exhibited 12 sharp imino peaks with a cross-peak pattern similar to DT2-163 (not shown), whereas 3'T2-163 had 1D and 2D NMR spectra remarkably similar to 163 with the same five Gs in the *syn* conformation (G1, G5, G14, G15 and G20). Nevertheless, the 3'T2-163 sample was a mixture of two species: the predominant species (∼80%) had similar peak dispersion and NOESY cross peaks to 163; and the minor species (∼20%) had spectral characteristics similar to those of DT2-163.

### Flanking sequences influence function

To demonstrate that the flanking sequences play roles in G4 functions, a well-known hemin aptamer, PS2.M (33), was chosen as an example. The results exhibited that the addition of flanking nucleotides affects its conformation from hybrid to parallel (Supplementary Figure S27A), its affinity for hemin (*K*_d_ increases from 3.78 to 10.43 μM; Supplementary Figures S27B and S28), and its catalytic activity (Supplementary Figures S27C and D). The flanking effect on the catalytic activity was also confirmed by other sequences (Supplementary Figure S29).

## DISCUSSION

Most structural studies on G-rich motifs have focused on the core G4 region and rarely included the nucleotide sequences at either end. Even in machine learning (34), sequencing (35), and bioinformatics approaches (36), the impact of the flanking nucleotides on the structure is rarely considered. This is surprising, given that flanking nucleotides are known to play a major role in telomeric G4 topology (14,15).

Here, the effect of flanking sequences was investigated by analyses of over 300 different sequences based on CD analysis. These sequences formed G4s with loops of different lengths, different core G4 topologies, different nucleotide compositions, and overhang lengths. The results allowed us to draw the general conclusion that flanking nucleotides had a significant impact on G4 topology, favoring a parallel fold. Many, but not all, sequences easily converted to a parallel topology, either by adding K^+^ or terminal nucleotides (Supplementary Figure S30). It should be noted that these results are derived from CD analysis, which is sufficient to determine topology with excellent accuracy, but it is a low-resolution technique that cannot provide detailed structural information on the G4 structure (17).

Furthermore, the flanking effect was found to be valid for natural sequences with diverse nucleotide compositions of loops and flanking segments, and extended to G4s involving adenines, cytosines or guanines as flanking nucleotides (Supplementary Figure S23), adenines in the loops and the flanking regions (Supplementary Figure S31), and for those with four quartets (Supplementary Figure S32). The nucleotides at the 5′-end have a stronger effect than those at the 3′-end, illustrating a 5′-3′ asymmetry in G4 folding previously described (37). Beyond general effects, subtle variations and exceptions were found, especially in K^+^. Previously, we showed that loop permutation affects the topology and stability of G4 (6). Herein, we verified that the sequences with the shortest loop in the central position are the most sensitive to the flanking effect. The permutation and combination of loops will indeed affect the formation of G4 conformation, which in turn tunes the occurrence and magnitude of the flanking effect. In addition, for the non-classical G4 structures with G-vacancy (38,39) or a bulge (40), which mostly adopt a parallel conformation, the presence of flanking sequences maintains the original topology (Supplementary Figure S33). More importantly, for the single sequence with a bulge that adopted an antiparallel fold, the addition of flanking nucleotides converted it to a more parallel conformation, showing that this flanking effect was still valid for this non-canonical G4.

Using MD simulations and NMR structural analysis, we demonstrated that the 5′-flanking effect is caused, at least in part, by the abrogation of a *syn*-specific intra-nucleotide hydrogen bond between the 5′-OH and terminal 5′-*syn* G. When this 5′-OH group is made unavailable by the presence of 5′-nucleotides or even a simple phosphate, this hydrogen bond is lost and the *syn* conformation is less favored. Consequently, this stabilizing contribution to non-parallel conformations is no longer present, explaining why the sequences tend to adopt a more parallel character when nucleotides flank the G4 core. At the same time, the substantial systematic 5′-end effect consistently seen in our experiments can be considered as an ultimate validation and quantification of the suggested role of the 5′-terminal hydrogen bond. In addition, the terminal thymines (and likely also other nucleotides) can interact with and stabilize the 5′-end G-quartet through diverse non-covalent interactions such as van der Waals interactions and ion-base coordination contacts. These, however, probably do not exert a strong effect on the *syn* versus *anti* balance. Furthermore, the terminal thymines can also stabilize the unfolded state, so their overall free-energy effect is not easy to predict and may be variable.

Undeniably, there are still some outliers which do not follow this flanking effect rule. According to previous theoretical simulations (32), the terminal guanines of hybrid G4s adopt a *syn* conformation, while antiparallel G4 can adopt both *syn* and *anti* conformations. Therefore, in non-parallel assembles, the addition of flanking nucleotides may reduce the population of hybrid (with first *syn*-G) in favor of antiparallel (with first *anti*-G) while not detectably increasing the population of the parallel folds, so the flanking nucleotides could sometimes lead to reduced ‘parallelization’ based on the *r* parameter.

## CONCLUSION

In summary, this study illustrates that G4 motifs cannot be considered as isolated islands: Interactions among G-quartets, loops, flanking sequences, and bulges or other structural imperfections must be taken into account, especially when considering G4 genomic structures with important physiological functions that may serve as drug targets or when designing G4 motifs to be attached to a surface, nanoparticle, or biomolecule via a nucleotidic linker. In the present work, we systematically analyzed the generality, mechanism, and potential application impacts of the flanking effect. The ultimate goal will be to expand this study in a more chromatin-like environment, with long 5′ and 3′ extensions, in the presence of the C-rich complementary strand and nuclear proteins.

## Supplementary Material

gkab681_Supplemental_File

## References

[1] Mergny J.-L., Sen D. DNA quadruple helices in nanotechnology. Chem. Rev. 2019; 119:6290–6325. 30605316 10.1021/acs.chemrev.8b00629

[2] Chaires J.B., Graves D. Quadruplex Nucleic Acids. 2013; Springer.

[3] Kwok C.K., Merrick C.J. G-Quadruplexes: prediction, characterization, and biological application. Trends Biotechnol. 2017; 35:997–1013. 28755976 10.1016/j.tibtech.2017.06.012

[4] Tian T., Chen Y.-Q., Wang S.-R., Zhou X. G-Quadruplex: a regulator of gene expression and its chemical targeting. Chem. 2018; 4:1314–1344.

[5] Spiegel J., Adhikari S., Balasubramanian S. The structure and function of DNA G-quadruplexes. Trends Chem. 2020; 2:123–136. 32923997 10.1016/j.trechm.2019.07.002 PMC7472594

[6] Cheng M., Cheng Y., Hao J., Jia G., Zhou J., Mergny J.-L., Li C. Loop permutation affects the topology and stability of G-quadruplexes. Nucleic Acids Res. 2018; 46:9264–9275. 30184167 10.1093/nar/gky757 PMC6182180

[7] Risitano A., Fox K.R. Influence of loop size on the stability of intramolecular DNA quadruplexes. Nucleic Acids Res. 2004; 32:2598–2606. 15141030 10.1093/nar/gkh598 PMC419475

[8] Hazel P., Huppert J., Balasubramanian S., Neidle S. Loop-length-dependent folding of G-quadruplexes. J. Am. Chem. Soc. 2004; 126:16405–16415. 15600342 10.1021/ja045154j

[9] Smargiasso N., Rosu F., Hsia W., Colson P., Baker E.S., Bowers M.T., De Pauw E., Gabelica V. G-quadruplex DNA assemblies: loop length, cation identity, and multimer formation. J. Am. Chem. Soc. 2008; 130:10208–10216. 18627159 10.1021/ja801535e

[10] Kettani A., Bouaziz S., Wang W., Jones R.A., Patel D.J. Bombyx mori single repeat telomeric DNA sequence forms a G-quadruplex capped by base triads. Nat. Struct. Biol. 1997; 4:382–389. 9145109 10.1038/nsb0597-382

[11] Do N.Q., Phan A.T. Monomer-dimer equilibrium for the 5′-5′ stacking of propeller-type parallel-stranded G-quadruplexes: NMR structural study. Chem. Eur. J. 2012; 18:14752–14759. 23019076 10.1002/chem.201103295

[12] Pavc D., Wang B., Spindler L., Drevensek-Olenik I., Plavec J., Sket P. GC ends control topology of DNA G-quadruplexes and their cation-dependent assembly. Nucleic Acids Res. 2020; 48:2749–2761. 31996902 10.1093/nar/gkaa058 PMC7049726

[13] Hatzakis E., Okamoto K., Yang D. Thermodynamic stability and folding kinetics of the major G-quadruplex and its loop isomers formed in the nuclease hypersensitive element in the human c-Myc promoter: effect of loops and flanking segments on the stability of parallel-stranded intramolecular G-quadruplexes. Biochemistry. 2010; 49:9152–9160. 20849082 10.1021/bi100946g PMC2964402

[14] Zhang Z., Dai J., Veliath E., Jones R.A., Yang D. Structure of a two-G-tetrad intramolecular G-quadruplex formed by a variant human telomeric sequence in K^+^ solution: insights into the interconversion of human telomeric G-quadruplex structures. Nucleic Acids Res. 2010; 38:1009–1021. 19946019 10.1093/nar/gkp1029 PMC2817458

[15] Phan A.T. Human telomeric G-quadruplex: structures of DNA and RNA sequences. FEBS J. 2010; 277:1107–1117. 19951353 10.1111/j.1742-4658.2009.07464.x

[16] Dai J., Carver M., Yang D. Polymorphism of human telomeric quadruplex structures. Biochimie. 2008; 90:1172–1183. 18373984 10.1016/j.biochi.2008.02.026 PMC2556180

[17] Del Villar-Guerra R., Trent J.O., Chaires J.B. G-Quadruplex secondary structure obtained from circular dichroism spectroscopy. Angew. Chem. Int. Ed. 2018; 57:7171–7175. 10.1002/anie.201709184 PMC5920796 29076232

[18] Mergny J.-L., Phan A.T., Lacroix L. Following G-quartet formation by UV-spectroscopy. FEBS Lett. 1998; 435:74–78. 9755862 10.1016/s0014-5793(98)01043-6

[19] Mergny J.-L., Li J., Lacroix L., Amrane S., Chaires J.B. Thermal difference spectra: a specific signature for nucleic acid structures. Nucleic Acids Res. 2005; 33:e138. 16157860 10.1093/nar/gni134 PMC1201377

[20] Phan A.T. Long-range imino proton-13C J-couplings and the through-bond correlation of imino and non-exchangeable protons in unlabeled DNA. J. Biomol. NMR. 2000; 16:175–178. 10723997 10.1023/a:1008355231085

[21] Clark G.R., Pytel P.D., Squire C.J. The high-resolution crystal structure of a parallel intermolecular DNA G-4 quadruplex/drug complex employing syn glycosyl linkages. Nucleic Acids Res. 2012; 40:5731–5738. 22373921 10.1093/nar/gks193 PMC3384316

[22] Wang Y., Patel D.J. Solution structure of the human telomeric repeat d[AG3(T2AG3)3] G-tetraplex. Structure. 1993; 1:263–282. 8081740 10.1016/0969-2126(93)90015-9

[23] Luu K.N., Phan A.T., Kuryavyi V., Lacroix L., Patel D.J. Structure of the human telomere in K^+^ solution: an intramolecular (3+1) G-quadruplex scaffold. J. Am. Chem. Soc. 2006; 128:9963–9970. 16866556 10.1021/ja062791w PMC4692383

[24] Kollman P.A., Massova I., Reyes C., Kuhn B., Huo S., Chong L., Lee M., Lee T., Duan Y., Wang W. et al. Calculating structures and free energies of complex molecules: combining molecular mechanics and continuum models. Acc. Chem. Res. 2000; 33:889–897. 11123888 10.1021/ar000033j

[25] Islam B., Stadlbauer P., Neidle S., Haider S., Sponer J. Can we execute reliable MM-PBSA free energy computations of relative stabilities of different guanine quadruplex folds. J. Phys. Chem. B. 2016; 120:2899–2912. 26918369 10.1021/acs.jpcb.6b01059

[26] Case D., Ben-Shalom I., Brozell S., Cerutti D., Cheatham T. III, Cruzeiro V., Darden T., Duke R., Ghoreishi D., Gilson M.K. et al. 2018; San Francisco. AMBER 2018.

[27] Zgarbova M., Šponer J., Otyepka M., Cheatham T.E. 3rd, Galindo-Murillo R., Jurecka P. Refinement of the sugar-phosphate backbone torsion beta for AMBER force fields improves the description of Z- and B-DNA. J. Chem. Theory Comput. 2015; 11:5723–5736. 26588601 10.1021/acs.jctc.5b00716

[28] Largy E., Marchand A., Amrane S., Gabelica V., Mergny J.-L. Quadruplex turncoats: cation-dependent folding and stability of quadruplex-DNA double switches. J. Am. Chem. Soc. 2016; 138:2780–2792. 26837276 10.1021/jacs.5b13130

[29] Dvorkin S.A., Karsisiotis A.I., Webba da Silva M. Encoding canonical DNA quadruplex structure. Sci. Adv. 2018; 4:eaat3007. 30182059 10.1126/sciadv.aat3007 PMC6118410

[30] Largy E., Mergny J.-L. Shape matters: size-exclusion HPLC for the study of nucleic acid structural polymorphism. Nucleic Acids Res. 2014; 42:e149. 25143531 10.1093/nar/gku751 PMC4231728

[31] Cang X., Šponer J., Cheatham T.E. 3rd Explaining the varied glycosidic conformational, G-tract length and sequence preferences for anti-parallel G-quadruplexes. Nucleic Acids Res. 2011; 39:4499–4512. 21296760 10.1093/nar/gkr031 PMC3105399

[32] Šponer J., Mladek A., Spackova N., Cang X.H., Cheatham T.E., Grimme S. Relative stability of different DNA guanine quadruplex stem topologies derived using large-scale quantum-chemical computations. J. Am. Chem. Soc. 2013; 135:9785–9796. 23742743 10.1021/ja402525c PMC3775466

[33] Travascio P., Li Y., Sen D. DNA-enhanced peroxidase activity of a DNA-aptamer-hemin complex. Chem. Biol. 1998; 5:505–517. 9751647 10.1016/s1074-5521(98)90006-0

[34] Puig Lombardi E., Londono-Vallejo A. A guide to computational methods for G-quadruplex prediction. Nucleic Acids Res. 2020; 48:1–15. 31943112 10.1093/nar/gkaa033 PMC7026631

[35] Rodriguez R., Miller K.M., Forment J.V., Bradshaw C.R., Nikan M., Britton S., Oelschlaegel T., Xhemalce B., Balasubramanian S., Jackson S.P. Small-molecule-induced DNA damage identifies alternative DNA structures in human genes. Nat. Chem. Biol. 2012; 8:301–310. 22306580 10.1038/nchembio.780 PMC3433707

[36] Chambers V.S., Marsico G., Boutell J.M., Di Antonio M., Smith G.P., Balasubramanian S. High-throughput sequencing of DNA G-quadruplex structures in the human genome. Nat. Biotechnol. 2015; 33:877–881. 26192317 10.1038/nbt.3295

[37] Gray R.D., Trent J.O., Chaires J.B. Folding and unfolding pathways of the human telomeric G-quadruplex. J. Mol. Biol. 2014; 426:1629–1650. 24487181 10.1016/j.jmb.2014.01.009 PMC3969789

[38] Li X.M., Zheng K.W., Zhang J.Y., Liu H.H., Yuan B.F., Hao Y.H., Tan Z. Guanine-vacancy–bearing G-quadruplexes responsive to guanine derivatives. Proc. Natl. Acad. Sci. U.S.A. 2015; 112:14581–14586. 26553979 10.1073/pnas.1516925112 PMC4664295

[39] Winnerdy F.R., Das P., Heddi B., Phan A.T. Solution structures of a G-quadruplex bound to linear-and cyclic-dinucleotides. J. Am. Chem. Soc. 2019; 141:18038–18047. 31661272 10.1021/jacs.9b05642

[40] Mukundan V.T., Phan A.T. Bulges in G-quadruplexes: broadening the definition of G-quadruplex-forming sequences. J. Am. Chem. Soc. 2013; 135:5017–5028. 23521617 10.1021/ja310251r

