# Supplementary material for: The beginning and the end: flanking nucleotides induce a parallel G-quadruplex topology
Source: Nucleic Acids Res. 2021 Aug 11;49(16):9548–59. doi: 10.1093/nar/gkab681 (PMC8450091; doi:10.1093/nar/gkab681)
Supplement: gkab681_Supplemental_File [file gkab681_supplemental_file.docx]

**Supplementary Information for**

**The beginning and the end: Flanking nucleotides induce a parallel G-quadruplex topology**

Jielin Chen^1,#^, Mingpan Cheng^1,2,#^, Gilmar F. Salgado^2,#^, Petr Stadlbauer^3,4^, Xiaobo Zhang^1^, Samir Amrane^2^, Aurore Guédin^2^, Fangni He^1^, Jiří Šponer^3,4^, Huangxian Ju^1,^*, Jean-Louis Mergny^1,2,3,5,^*, and Jun Zhou^1,^*

^1^ State Key Laboratory of Analytical Chemistry for Life Science, School of Chemistry & Chemical Engineering, Nanjing University, Nanjing 210023, China.

^2^ ARNA Laboratory, Université de Bordeaux, Inserm U1212, CNRS UMR5320, IECB, Pessac 33607, France.

^3^ Institute of Biophysics of the Czech Academy of Sciences, Královopolská 135, 612 65 Brno, Czech Republic.

^4^ Regional Centre of Advanced Technologies and Materials, Czech Advanced Technology and Research Institute (CATRIN), Palacky University Olomouc, Šlechtitelů 241/27,783 71, Olomouc – Holice, Czech Republic.

^5^ Laboratoire d’Optique et Biosciences, Ecole Polytechnique, CNRS, Inserm, Institut Polytechnique de Paris, 91128 Palaiseau cedex, France.

^#^ These authors contributed equally to this work.

*Emails: hxju@nju.edu.cn; jean-louis.mergny@polytechnique.edu; jun.zhou@nju.edu.cn.

**Content**

Full Computational DetailsS3

Table S1S6

Table S2S7

Table S3S10

Table S4S11

Table S5S12

Table S6S13

Table S7S14

Table S8S16

Table S9S17

Table S10S18

Table S11S18

Table S12S19

Figure S1S23

Figure S2S24

Figure S3S25

Figure S4S26

Figure S5S27

Figure S6S28

Figure S7S29

Figure S8S30

Figure S9S31

Figure S10S32

Figure S11S33

Figure S12S34

Figure S13S35

Figure S14S36

Figure S15S37

Figure S16S38

Figure S17S39

Figure S18S40

Figure S19S41

Figure S20S42

Figure S21S43

Figure S22S44

Figure S23S45

Figure S24S46

Figure S25S47

Figure S26S48

Figure S27S49

Figure S28S50

Figure S29S51

Figure S30S52

Figure S31S53

Figure S32S54

Figure S33S55

ReferencesS56

**Full Computational Details**

**System preparation.** The influence of *anti* and *syn* guanines on stability of different G4 folds was assessed on ten different G4 models that were built for MD simulations. The parallel-stranded models were built up from the tetramolecular parallel structure of (GGGG)_4_ (PDB ID: 3TVB (S1)) by removal of either the 5ʹ or 3ʹ terminal quartet to achieve an all-*anti* structure or 5ʹ-*syn* structure, respectively. Two human telomeric structures, namely the antiparallel basket (PDB ID: 143D (S2)) and (3+1) hybrid type-1 (PDB ID: 2GKU (S3)), were taken as a basis for the antiparallel and (3+1) hybrid model, respectively. Modelling of terminal thymines was done in the xleap module of AMBER (S4). Based on our extensive experience with G-quadruplex simulations we suggest that the present simulation timescale is entirely sufficient to eliminate any influence stemming from the specific experimental structures used to prepare the models. In other words, usage of any other experimental structures with the same topologies would be equivalent.

Each model was solvated in a truncated octahedral box of SPC/E water molecules (S5) with a distance of at least 10 Å between the G4 and the box border. Two K^+^ cations were placed inside the G4 channel between the quartets. The simulation box was further neutralized with K^+^ cations and then 150 mM excess KCl was added (S6). The systems were simulated using the latest AMBER OL15 version (S7) of the Cornell *et al*. force field (S8), which integrates several dihedral-potential modifications (S9-S11).

**Equilibration**. Starting structures were equilibrated using standard protocol. Each system was first minimized with 500 steps of steepest descent followed by 500 steps of conjugate gradient minimization with 25 kcal·mol^-1^·A^-2^ position restraints on DNA atoms. It was then heated from 0 to 300 K during 100 ps with constant volume and position restraints of 25 kcal·mol^-1^·A^-2^. Minimization with 5 kcal·mol^-1^·A^-2^ restraints followed, using 500 steps of steepest descent method and 500 steps of conjugate gradient. The restraints of 5 kcal·mol^-1^·A^-2^ were maintained on DNA atoms and the system was equilibrated for 50 ps at constant temperature of 300 K and pressure of 1 atm. An analogous series of alternating minimizations and equilibrations followed using decreasing position restraints of 4, 3, 2 and 1 kcal·mol^-1^·A^-2^ consecutively. The final equilibration was carried out with position restraints of 0.5 kcal·mol^-1^·A^-2^ and starting velocities from the previous equilibration, followed by a short free MD simulation of 50 ps. Temperature and pressure coupling time constant during equilibration was set to 0.2 ps, and during the last molecular dynamics phase to 5 ps.

**Production Dynamics**. Production runs were carried out after the equilibration stage. Each system was simulated once for 2.5 µs. The temperature was set to 300 K using Langevin thermostat with a collision frequency of 2 ps^-1^, and the pressure was held at 1 bar by Berendsen barostat. Electrostatic interactions were treated by the Particle mesh Ewald method (S12). Non-bonded cut-off was set to 9 Å. Covalent bonds with hydrogen in solute and solvent were constrained by SHAKE (S13) and SETTLE (S14), respectively. The hydrogen mass repartitioning method was employed (S15). The integration time step was thus set to 4 fs. The calculations were done using the CUDA version of the pmemd module of AMBER 18 (S4,S16).

**Free-Energy Calculation**. To obtain insights into energetics of the different G-quadruplex models, we performed MM-PBSA calculation (S17,S18) and an hydrogen-bond population analysis based on the calculated trajectories.

For evaluation of relative free-energy differences (Δ*G*) of various G-stems (Figure S20), we employed the MM-PBSA method as implemented in the python script of AMBER 18 (S4). MM-PBSA is an approximate free-energy method which is based on post-processing of explicit-solvent MD trajectories using the continuum solvent approximation.

Snapshots from the production dynamics were taken every 2 ns, starting from 501 ns to 2500 ns (*i.e.*, first 500 ns of each trajectory was not taken into account). To ensure that exactly the same number of atoms would be considered in various G4s, so their free energy could be compared directly, trajectories for MM-PBSA calculation were processed as follows: only the three-quartet stems of a G4 were considered, *i.e.*, the flanking nucleotides were excluded from the MM-PBSA analysis despite being present in the simulation. If a flanking nucleotide was removed from the 5ʹ-end of a strand, the connecting phosphate bridge (formally belonging to the G-stem) was also removed; if there was no 5ʹ-terminal flanking nucleotide to be removed from a strand, its 3ʹ-terminal HO3ʹ atom was removed instead. In this way, the calculated free-energies of all systems can be directly compared. Further, the MM-PBSA energies evaluated in this way reflect only internal free energies of the different G-stems (including the 5ʹ-terminal H-bond) while other contributions of the flanking nucleotides (including, e.g., their stacking interactions with the stem) are excluded from the computation. This can be considered as an optimal protocol to assess the net effect of the 5ʹ-terminal H-bond.

Two channel cations were retained while other ions and waters were stripped off. Solvent probe radius was set to 1.6 Å and internal AMBER pre-computed atomic radii were used. Entropic contributions were not included in the calculation for we expected similar values in the models that were to be compared.

Independently of the MM-PBSA calculation, we performed population analysis of the 5ʹ-OH···N3 H-bond in 5ʹ-terminal Gs in the *syn* conformation (see models **1b**, **1c** and **1d** in Figure S20) to estimate Δ*G* of the H-bond formation. The population fraction of the H-bond presence *p*_HB_ was used to calculate Δ*G* according to the formula Δ*G* = –RTln(*p*_HB_/(1-*p*_HB_)). The boundary for the bond’s presence was set to O5ʹ···N3 distance of 3.5 Å and the O5ʹ-HO5ʹ···N3 angle needed to be greater than 135 degrees. We note that any such cut-off geometrical H-bond criteria is inevitably arbitrary. Nevertheless, the calculated populations are fairly insensitive to moderate variations in the cut-offs.

It has been suggested that DNA chains can form also internal (intranucleotide) base···phosphate interaction between the phosphate group and the amino group of a *syn*-oriented guanosine-5ʹ-phosphate within the nucleotide. Such interaction can contribute to stability of the *syn* orientation of the χ dihedral angle. This interaction is present, for example, in some (but not all) *syn*-guanines in NMR models of human telomeric G-quadruplexes (2MBJ (S19), 2KF8 (S20), 2KKA (S21), 2JSM (S22), 2GKU (S3), 2HY9 (S23), 2JSL (S3,S24) 143D (S2). The interaction has been observed in MD simulations of some G-hairpins that may can participate in folding pathways of G-quadruplexes (S25), and has been predicted for nucleotides in some Z-DNA backbone conformational states (S26). Thus, it could potentially compensate or partly compensate for the loss of the 5ʹ-terminal *syn*-specific 5ʹ-OH···N3 H-bond upon adding the 5’-end flanking sequence. We have therefore performed a population analysis of this interaction where applicable (see models **2b**, **3b**, **3c** and **3d** in Figure S20). In addition, we have analysed our older MD simulation trajectories of the human telomeric quadruplexes (S27) and inspected whether the base···phosphate interaction was present in those. The interaction was considered present if any of the distances X···N2 and angles X···H22-N2 was shorter than 3.5 Å and greater than 135 degrees, respectively, where X is O5ʹ, OP1, OP2 or O3ʹ (of the preceding thymine).

**Table S1**. Intramolecular G4 structure formed by human telomere sequences with different flanking nucleotides in K^+^ buffer.

| Topologies | Sequence (5'-3') *^a^* | PDB ID | References |
| --- | --- | --- | --- |
| Antiparallel | G_3_(T_2_AG_3_)_3_T | 2KF8 | (S20) |
|  | AG_3_(T_2_AG_3_)_3_T *^b^* | 2KKA | (S21) |
| Hybrid-1 | TAG_3_(T_2_AG_3_)_3_ | 2JSM | (S22) |
|  | T_2_G_3_(T_2_AG_3_)_3_A | 2GKU | (S3) |
|  | A_3_G_3_(T_2_AG_3_)_3_A | 2HY9 | (S23) |
| Hybrid-2 | TAG_3_(T_2_AG_3_)_3_T_2_ | 2JSL | (S22,S24) |
|  | T_2_AG_3_(T_2_AG_3_)_3_T_2_ | 2JPZ | (S28) |

*^a^* The core of human telomere sequence is marked with red color.

*^b^* In this sequence, G14 is substituted by inosine.

**Table S2**. Information of artificial design sequences and their conformation index *r* of sequences with (*r*_DT2_), without (*r*_WO_) flanking nucleotides and their difference (Δ*r* = *r*_DT2_ - *r*_WO_) in 100 mM KCl or NaCl.

| Name | Sequence (5'→3') | *r*_WO_ | *r*_DT2_ | Δ*r* | *r*_WO_ | *r*_DT2_ | Δ*r* |
| --- | --- | --- | --- | --- | --- | --- | --- |
|  |  | K^+^ | | | Na^+^ | | |
| ***133*** *Group* |  |  |  |  |  |  |  |
| 133 | G_3_ T G_3_ T_3_ G_3_ T_3_ G_3_ | 0.82 | 0.79 | -0.03 | -0.21 | 0.59 | 0.80 |
| 313 | G_3_ T_3_ G_3_ T G_3_ T_3_ G_3_ | 0.93 | 0.93 | 0.00 | 0.13 | 0.38 | 0.25 |
| 331 | G_3_ T_3_ G_3_ T_3_ G_3_ T G_3_ | 0.89 | 0.83 | -0.06 | -0.11 | 0.59 | 0.70 |
| ***144*** *Group* |  |  |  |  |  |  |  |
| 144 | G_3_ T G_3_ T_4_ G_3_ T_4_ G_3_ | 0.39 | 0.86 | 0.47 | 0.22 | 0.51 | 0.29 |
| 414 | G_3_ T_4_ G_3_ T G_3_ T_4_ G_3_ | 0.68 | 0.94 | 0.26 | -0.27 | 0.21 | 0.48 |
| 441 | G_3_ T_4_ G_3_ T_4_ G_3_ T G_3_ | 0.46 | 0.89 | 0.43 | -0.45 | 0.51 | 0.96 |
| ***155*** *Group* |  |  |  |  |  |  |  |
| 155 | G_3_ T G_3_ T_5_ G_3_ T_5_ G_3_ | 0.54 | 0.84 | 0.30 | 0.44 | 0.48 | 0.04 |
| 515 | G_3_ T_5_ G_3_ T G_3_ T_5_ G_3_ | 0.79 | 0.93 | 0.14 | -0.05 | 0.52 | 0.57 |
| 551 | G_3_ T_5_ G_3_ T_5_ G_3_ T G_3_ | 0.41 | 0.90 | 0.49 | -0.13 | 0.48 | 0.61 |
| ***166*** *Group* |  |  |  |  |  |  |  |
| 166 | G_3_ T G_3_ T_6_ G_3_ T_6_ G_3_ | 0.66 | 0.88 | 0.22 | 0.59 | 0.48 | -0.11 |
| 616 | G_3_ T_6_ G_3_ T G_3_ T_6_ G_3_ | 0.87 | 0.92 | 0.05 | 0.18 | 0.46 | 0.28 |
| 661 | G_3_ T_6_ G_3_ T_6_ G_3_ T G_3_ | 0.45 | 0.90 | 0.45 | 0.21 | 0.37 | 0.16 |
| ***233*** *Group* |  |  |  |  |  |  |  |
| 233 | G_3_ T_2_ G_3_ T_3_ G_3_ T_3_ G_3_ | 0.39 | 0.47 | 0.08 | -0.39 | 0.31 | 0.70 |
| 323 | G_3_ T_3_ G_3_ T_2_ G_3_ T_3_ G_3_ | 0.50 | 0.53 | 0.03 | -0.29 | 0.31 | 0.60 |
| 332 | G_3_ T_3_ G_3_ T_3_ G_3_ T_2_ G_3_ | 0.40 | 0.53 | 0.13 | -0.23 | 0.40 | 0.63 |
| ***244*** *Group* |  |  |  |  |  |  |  |
| 244 | G_3_ T_2_ G_3_ T_4_ G_3_ T_4_ G_3_ | 0.25 | 0.55 | 0.30 | -0.05 | 0.20 | 0.25 |
| 424 | G_3_ T_4_ G_3_ T_2_ G_3_ T_4_ G_3_ | -0.13 | 0.27 | 0.40 | -0.31 | -0.22 | 0.09 |
| 442 | G_3_ T_4_ G_3_ T_4_ G_3_ T_2_ G_3_ | -0.16 | 0.60 | 0.76 | -0.32 | -0.04 | 0.28 |
| ***134*** *Group* |  |  |  |  |  |  |  |
| 134 | G_3_ T G_3_ T_3_ G_3_ T_4_ G_3_ | 0.74 | 0.87 | 0.13 | 0.10 | 0.62 | 0.52 |
| 143 | G_3_ T G_3_ T_4_ G_3_ T_4_ G_3_ | 0.36 | 0.78 | 0.42 | 0.17 | 0.40 | 0.23 |
| 314 | G_3_ T_3_ G_3_ T G_3_ T_4_ G_3_ | 0.93 | 0.96 | 0.03 | -0.38 | 0.49 | 0.87 |
| 341 | G_3_ T_3_ G_3_ T_4_ G_3_ T G_3_ | 0.53 | 0.87 | 0.34 | -0.44 | 0.56 | 1.00 |
| 413 | G_3_ T_4_ G_3_ T G_3_ T_3_ G_3_ | 0.91 | 0.94 | 0.03 | 0.07 | 0.43 | 0.36 |
| 431 | G_3_ T_4_ G_3_ T_3_ G_3_ T G_3_ | 0.88 | 0.88 | 0.00 | -0.30 | 0.57 | 0.87 |
|  |  |  |  |  |  |  |  |
|  |  |  |  |  |  |  |  |
|  |  |  |  |  |  |  |  |
|  |  |  |  |  |  |  |  |
|  |  |  |  |  |  |  |  |
|  |  |  |  |  |  |  |  |
| **Table S2**. Continued | |  |  |  |  |  |  |
| Name | Sequence (5'→3') | *r*_WO_ | *r*_DT2_ | Δ*r* | *r*_WO_ | *r*_DT2_ | Δ*r* |
|  |  | K^+^ | | | Na^+^ | | |
| ***135*** *Group* |  |  |  |  |  |  |  |
| 135 | G_3_ T G_3_ T_3_ G_3_ T_5_ G_3_ | 0.63 | 0.84 | 0.21 | 0.42 | 0.62 | 0.20 |
| 153 | G_3_ T G_3_ T_5_ G_3_ T_3_ G_3_ | 0.44 | 0.76 | 0.32 | 0.27 | 0.41 | 0.14 |
| 315 | G_3_ T_3_ G_3_ T G_3_ T_5_ G_3_ | 0.91 | 0.94 | 0.03 | -0.26 | 0.55 | 0.81 |
| 351 | G_3_ T_3_ G_3_ T_5_ G_3_ T G_3_ | 0.57 | 0.84 | 0.27 | -0.08 | 0.53 | 0.61 |
| 513 | G_3_ T_5_ G_3_ T G_3_ T_3_ G_3_ | 0.90 | 0.94 | 0.04 | 0.25 | 0.53 | 0.28 |
| 531 | G_3_ T_5_ G_3_ T_3_ G_3_ T G_3_ | 0.88 | 0.88 | 0.00 | -0.20 | 0.58 | 0.78 |
| ***136*** *Group* |  |  |  |  |  |  |  |
| 136 | G_3_ T G_3_ T_3_ G_3_ T_6_ G_3_ | 0.61 | 0.83 | 0.22 | 0.50 | 0.61 | 0.11 |
| 163 | G_3_ T G_3_ T_6_ G_3_ T_3_ G_3_ | 0.46 | 0.87 | 0.41 | 0.36 | 0.47 | 0.11 |
| 316 | G_3_ T_3_ G_3_ T G_3_ T_6_ G_3_ | 0.91 | 0.93 | 0.02 | -0.18 | 0.61 | 0.79 |
| 361 | G_3_ T_3_ G_3_ T_6_ G_3_ T G_3_ | 0.60 | 0.83 | 0.23 | 0.34 | 0.59 | 0.25 |
| 613 | G_3_ T_6_ G_3_ T G_3_ T_3_ G_3_ | 0.89 | 0.93 | 0.04 | 0.38 | 0.58 | 0.20 |
| 631 | G_3_ T_6_ G_3_ T_3_ G_3_ T G_3_ | 0.79 | 0.89 | 0.10 | 0.02 | 0.58 | 0.56 |
| ***145*** *Group* |  |  |  |  |  |  |  |
| 145 | G_3_ T G_3_ T_4_ G_3_ T_5_ G_3_ | 0.48 | 0.88 | 0.40 | 0.27 | 0.54 | 0.27 |
| 154 | G_3_ T G_3_ T_5_ G_3_ T_4_ G_3_ | 0.49 | 0.88 | 0.39 | 0.41 | 0.52 | 0.11 |
| 415 | G_3_ T_4_ G_3_ T G_3_ T_5_ G_3_ | 0.78 | 0.93 | 0.15 | -0.25 | 0.15 | 0.40 |
| 451 | G_3_ T_4_ G_3_ T_5_ G_3_ T G_3_ | 0.55 | 0.89 | 0.34 | -0.31 | 0.53 | 0.84 |
| 514 | G_3_ T_5_ G_3_ T G_3_ T_4_ G_3_ | 0.74 | 0.94 | 0.20 | -0.30 | 0.51 | 0.81 |
| 541 | G_3_ T_5_ G_3_ T_4_ G_3_ T G_3_ | 0.37 | 0.90 | 0.53 | -0.22 | 0.53 | 0.75 |
| ***234*** *Group* |  |  |  |  |  |  |  |
| 234 | G_3_ T_2_ G_3_ T_3_ G_3_ T_4_ G_3_ | 0.44 | 0.50 | 0.06 | -0.20 | 0.26 | 0.46 |
| 243 | G_3_ T_2_ G_3_ T_4_ G_3_ T_3_ G_3_ | 0.31 | 0.39 | 0.08 | -0.05 | 0.17 | 0.22 |
| 324 | G_3_ T_3_ G_3_ T_2_ G_3_ T_4_ G_3_ | 0.14 | 0.43 | 0.29 | -0.43 | 0.32 | 0.75 |
| 342 | G_3_ T_3_ G_3_ T_4_ G_3_ T_3_ G_3_ | 0.24 | 0.46 | 0.22 | -0.20 | 0.08 | 0.28 |
| 423 | G_3_ T_4_ G_3_ T_2_ G_3_ T_3_ G_3_ | 0.25 | 0.44 | 0.19 | -0.19 | 0.22 | 0.41 |
| 432 | G_3_ T_4_ G_3_ T_3_ G_3_ T_2_ G_3_ | 0.47 | 0.56 | 0.09 | -0.16 | 0.33 | 0.49 |
| ***235*** *Group* |  |  |  |  |  |  |  |
| 235 | G_3_ T_2_ G_3_ T_3_ G_3_ T_5_ G_3_ | 0.51 | 0.55 | 0.04 | 0.34 | 0.36 | 0.02 |
| 253 | G_3_ T_2_ G_3_ T_5_ G_3_ T_3_ G_3_ | 0.37 | 0.43 | 0.06 | 0.19 | 0.30 | 0.11 |
| 325 | G_3_ T_3_ G_3_ T_2_ G_3_ T_5_ G_3_ | 0.29 | 0.51 | 0.22 | -0.27 | 0.34 | 0.61 |
| 352 | G_3_ T_3_ G_3_ T_5_ G_3_ T_2_ G_3_ | 0.47 | 0.52 | 0.05 | 0.43 | 0.27 | -0.16 |
| 523 | G_3_ T_5_ G_3_ T_2_ G_3_ T_3_ G_3_ | 0.37 | 0.51 | 0.14 | -0.04 | 0.24 | 0.28 |
| 532 | G_3_ T_5_ G_3_ T_3_ G_3_ T_2_ G_3_ | 0.42 | 0.56 | 0.14 | -0.19 | 0.35 | 0.54 |
| ***334*** *Group* |  |  |  |  |  |  |  |
| 334 | G_3_ T_3_ G_3_ T_3_ G_3_ T_4_ G_3_ | 0.21 | 0.01 | -0.20 | -0.30 | 0.10 | 0.40 |
| 343 | G_3_ T_3_ G_3_ T_4_ G_3_ T_3_ G_3_ | 0.28 | -0.12 | -0.40 | -0.30 | -0.03 | 0.27 |
| 433 | G_3_ T_4_ G_3_ T_3_ G_3_ T_3_ G_3_ | 0.36 | 0.00 | -0.36 | 0.35 | 0.25 | -0.10 |
|  |  |  |  |  |  |  |  |
| **Table S2**. Continued | |  |  |  |  |  |  |
| Name | Sequence (5'→3') | *r*_WO_ | *r*_DT2_ | Δ*r* | *r*_WO_ | *r*_DT2_ | Δ*r* |
|  |  | K^+^ | | | Na^+^ | | |
| ***223*** *Group* |  |  |  |  |  |  |  |
| 223 | G_3_ T_2_ G_3_ T_2_ G_3_ T_3_ G_3_ | 0.91 | 0.77 | -0.14 | -0.30 | 0.39 | 0.69 |
| 232 | G_3_ T_2_ G_3_ T_3_ G_3_ T_2_ G_3_ | 0.82 | 0.73 | -0.09 | -0.30 | 0.36 | 0.66 |
| 322 | G_3_ T_3_ G_3_ T_2_ G_3_ T_2_ G_3_ | 0.84 | 0.66 | -0.18 | -0.06 | 0.47 | 0.53 |
| ***224*** *Group* |  |  |  |  |  |  |  |
| 224 | G_3_ T_2_ G_3_ T_2_ G_3_ T_4_ G_3_ | 0.91 | 0.89 | -0.02 | -0.29 | 0.42 | 0.71 |
| 242 | G_3_ T_2_ G_3_ T_4_ G_3_ T_2_ G_3_ | 0.49 | 0.79 | 0.30 | -0.01 | 0.15 | 0.16 |
| 422 | G_3_ T_4_ G_3_ T_2_ G_3_ T_2_ G_3_ | 0.88 | 0.87 | -0.01 | -0.30 | -0.24 | 0.06 |
| ***225*** *Group* |  |  |  |  |  |  |  |
| 225 | G_3_ T_2_ G_3_ T_2_ G_3_ T_5_ G_3_ | 0.92 | 0.88 | -0.04 | -0.13 | 0.40 | 0.53 |
| 252 | G_3_ T_2_ G_3_ T_5_ G_3_ T_2_ G_3_ | 0.47 | 0.76 | 0.29 | 0.51 | 0.19 | -0.32 |
| 522 | G_3_ T_5_ G_3_ T_2_ G_3_ T_2_ G_3_ | 0.90 | 0.84 | -0.06 | -0.23 | 0.46 | 0.69 |
| ***226*** *Group* |  |  |  |  |  |  |  |
| 226 | G_3_ T2 G_3_ T2 G_3_ T6 G_3_ | 0.86 | 0.90 | 0.04 | 0.18 | 0.43 | 0.25 |
| 262 | G_3_ T_2_ G_3_ T_6_ G_3_ T_2_ G_3_ | 0.52 | 0.89 | 0.37 | 0.60 | 0.42 | -0.18 |
| 622 | G_3_ T_6_ G_3_ T_2_ G_3_ T_2_ G_3_ | 0.91 | 0.87 | -0.04 | -0.12 | 0.43 | 0.55 |

Notes: Unless otherwise stated, 5 μM DNA were prepared in 10 mM lithium cacodylate (pH 7.2) buffer supplemented with 100 mM KCl (or NaCl), heated at 95 °C for 5 min, and then slowly annealed to room temperature.

**Table S3**. Information of 8 natural and 6 previously studied sequences. Their conformation index *r* of sequences with (*r*_DT2_), without (*r*_WO_) flanking nucleotides and their difference (Δ*r* = *r*_DT2_ - *r*_WO_) in 100 mM KCl or NaCl.

| Name ^a^ | Sequence (5'→3') | Description | K^+^ | | | Na^+^ | | |
| --- | --- | --- | --- | --- | --- | --- | --- | --- |
|  |  |  | *r*_WO_ | *r*_DT2_ | Δ*r* | *r*_WO_ | *r*_DT2_ | Δ*r* |
| Ara24 | G_3_ T_3_A G_3_ T_3_A G_3_ T_3_A G_3_ | Arabidopsis telomere | 0.24 | 0.31 | 0.07 | -0.39 | -0.45 | -0.06 |
| Tet22 | G_4_ T_2_ G_4_ T_2_ G_4_ T_2_ G_4_ | Tetrahymena telomere | 0.82 | 0.81 | -0.01 | 0.20 | 0.55 | 0.35 |
| Chla27 | G_3_ T_4_A G_3_ T_4_A G_3_ T_4_A G_3_ | Chlamydomonas telomere | 0.38 | 0.59 | 0.21 | -0.35 | -0.40 | -0.05 |
| Scer21 | G_3_ TGT G_3_ TGT G_3_ TGT G_3_ | S. cerevisiae telomere | 0.65 | 0.69 | 0.04 | -0.20 | 0.45 | 0.65 |
| Par21 | G_3_ T_3_ G_3_ T_3_ G_3_ T_3_ G_3_ | Paramecium telomere | 0.09 | 0.24 | 0.15 | -0.31 | 0.35 | 0.66 |
| Gla26 | G_4_ TCT G_3_ TGCTGT G_4_ TCT G_3_ | C. glabrata telomere | 0.51 | 0.64 | 0.13 | -0.46 | -0.27 | 0.19 |
| Oxy28 | G_4_ T_4_ G_4_ T_4_ G_4_ T_4_ G_4_ | Oxytricha telomere | 0.40 | 0.61 | 0.21 | -0.58 | -0.56 | 0.02 |
| Oxy30 | T G_4_ T_4_ G_4_ T_4_ G_4_ T_4_ G_4_ T | Oxytricha telomere | 0.65 | 0.60 | -0.05 | -0.59 | -0.56 | 0.03 |
| 5J4W | G_3_ T_3_ G_2_ T_4_ G_2_ T_2_ G_2_ | Artificially designed ^b^ | -0.46 | -0.44 | 0.02 | -0.52 | -0.38 | 0.14 |
| 5J6U | G_4_ T_3_ G_4_ T_4_ G_4_ A_2_ G_4_ | Artificially designed ^b^ | -0.20 | 0.37 | 0.57 | -0.61 | -0.60 | 0.01 |
| 2M6W | G_4_ T_2_ G_4_ T_4_ G_4_ A_2_ G_4_ | Artificially designed ^b^ | -0.14 | 0.53 | 0.67 | -0.61 | -0.57 | 0.04 |
| 5J05 | G_3_ T_3_ G_3_ T_4_ G_3_ A G_3_ | Artificially designed ^b^ | 0.16 | 0.84 | 0.68 | -0.54 | -0.20 | 0.34 |
| 2M6V | G_3_ T_2_ G_3_ T_4_ G_3_ T G_3_ | Artificially designed ^b^ | 0.93 | 0.97 | 0.04 | -0.49 | 0.62 | 1.11 |
| 5J4P | G_2_ T_3_ G_2_ T_4_ G_2_ T_3_ G_2_ | Artificially designed ^b^ | -0.43 | -0.40 | 0.03 | -0.53 | -0.49 | 0.04 |

^a^ The sequence names are the same to them in the references S29,S30.

^b^ These sequences are collected from reference S30.

**Table S4**. Conformation index *r* of natural sequences collected in 100 mM KCl. These sequences, named by the length of three natural loops, searched from BLAST, with or without both terminal natural nucleotides.

| Name | Sequence (5'→3') | Description | *r* |
| --- | --- | --- | --- |
| 143 ^a^ | G_3_ T G_3_ T_4_ G_3_ T_3_ G_3_ | Aquila chrysaetos | 0.34 |
| 143F | GT G_3_ T G_3_ T_4_ G_3_ T_3_ G_4_ T_2_ | Aquila chrysaetos | 0.84 |
| 343N1 | G_4_ T_3_ G_3_ T_4_ G_3_ T_3_ G_3_ | Brassica oleracea HDEM | 0.46 |
| 343NF1 | T_2_ G_4_ T_3_ G_3_ T_4_ G_3_ T_3_ G_3_ T_2_ | Brassica oleracea HDEM | 0.57 |
| 343N2 | G_3_ T_3_ G_3_ T_4_ G_3_ T_3_ G_4_ | Viga unguiculata cultivar xiabao 2 | 0.34 |
| 343NF2 | TC G_3_ T_3_ G_3_ T_4_ G_3_ T_3_ G_4_ T_2_ | Viga unguiculata cultivar xiabao 2 | 0.46 |
| 343N3 | G_4_ T_3_ G_3_ T_4_ G_3_ T_3_ G_4_ | Bony fishes | 0.49 |
| 343NF3 | T_2_ G_4_ T_3_ G_3_ T_4_ G_3_ T_3_ G_4_ T_2_ | Bony fishes | 0.70 |
| 242N1 | G_3_ T_2_ G_3_ T_4_ G_3_ CT G_3_ | Human chromosome 3 | 0.48 |
| 242NF1 | A_2_ G_3_ T_2_ G_3_ T_4_ G_3_ CT G_4_ A | Human chromosome 3 | 0.69 |
| 242N2 | G_3_ TA G_3_ T_4_ G_3_ T_2_ G_3_ | Human chromosome 15 | 0.22 |
| 242NF2 | A G_4_ TA G_3_ T_4_ G_3_ T_2_ G_3_ T_2_ | Human chromosome 15 | 0.34 |
| 252N | G_3_ TA G_3_ T_3_GT G_3_ T_2_ G_3_ | Human chromosome 10 | 0.63 |
| 252NF | CT G_3_ TA G_3_ T_3_GT G_3_ T_2_ G_3_ TG | Human chromosome 10 | 0.71 |
| 341N | G_3_ T_2_A G_3_ T_2_AT G_3_ A G_3_ | Human chromosome 4 | 0.47 |
| 341NF | CA G_3_ T_2_A G_3_ T_2_AT G_3_ A G_3_ AC | Human chromosome 4 | 0.81 |

^a^ The sequence was already mentioned in the design sequence tested above, so the previous name was kept here.

**Table S5**. Conformation index *r* of natural sequences gathered in 100 mM NaCl. These sequences, named by the length of three natural loops, searched from BLAST, with or without both terminal natural nucleotides.

| Name | Sequence (5'→3') | Description | *r* |
| --- | --- | --- | --- |
| 133N | G_3_ T G_3_ T_3_ G_3_ T_3_ G_2_ | Human chromosome 3 | -0.34 |
| 133NF | CT G_3_ T G_3_ T_3_ G_3_ T_3_ G_2_ T_2_ | Human chromosome 3 | 0.40 |
| 314N1 | G_3_ T_2_A G_3_ T G_3_ AT_3_ G_3_ | Human chromosome 20 | -0.35 |
| 314NF1 | CT G_3_ T_2_A G_3_ T G_3_ AT_3_ G_4_ C_2_ | Human chromosome 20 | 0.02 |
| 314N2 | G_3_ T_3_ G_3_ T G_3_ TGT_2_ G_3_ | Human chromosome 3 | -0.28 |
| 314NF2 | GA G_3_ T_3_ G_3_ T G_3_ TGT_2_ G_3_ A_2_ | Human chromosome 3 | 0.62 |
| 341N | G_3_ T_3_ G_3_ T_2_AT G_3_ T G_3_ | Human chromosome 15 | -0.03 |
| 341NF | CA G_3_ T_3_ G_3_ T_2_AT G_3_ T G_3_ TA | Human chromosome 15 | 0.58 |
| 431N1 | G_3_ T_4_ G_3_ T_2_A G_3_ T G_3_ | Human chromosome X | 0.15 |
| 431NF1 | GT G_3_ T_4_ G_3_ T_2_A G_3_ T G_4_ A | Human chromosome X | 0.57 |
| 431N2 | G_3_ T_2_AT G_3_ TGT G_3_ T G_3_ | Human chromosome X | 0.06 |
| 431NF2 | GA G_3_ T_2_AT G_3_ TGT G_3_ T G_3_ AG | Human chromosome X | 0.34 |
| 222N1 | G_3_ CT G_3_ T_2_ G_3_ T_2_ G_3_ | Human chromosome 6 | -0.48 |
| 222NF1 | T_2_ G_3_ CT G_3_ T_2_ G_3_ T_2_ G_3_ T_2_ | Human chromosome 6 | 0.57 |
| 222 ^a^ | G_3_ T_2_ G_3_ T_2_ G_3_ T_2_ G_3_ | Human chromosome 12 | -0.24 |
| 222F | AT G_3_ T_2_ G_3_ T_2_ G_3_ T_2_ G_3_ T_2_ | Human chromosome 12 | 0.59 |

^a^ This sequence was already mentioned in the design sequence tested above, so the previous name was hold.

**Table S6**. The average of *r* values (‾*r* ) and variance (*r-*δ) for every group in 100 mM KCl or NaCl.

| Groups | K^+^ | | | | Na^+^ | | | |
| --- | --- | --- | --- | --- | --- | --- | --- | --- |
|  | *‾r* (WO) | *r*-δ (WO) | *‾r* (DT2) | *r*-δ (DT2) | *‾r* (WO) | *r*-δ (WO) | *‾r* (DT2) | *r*-δ (DT2) |
| ***133*** | 0.88 | 0.06 | 0.85 | 0.07 | -0.06 | 0.17 | 0.52 | 0.12 |
| ***144*** | 0.51 | 0.16 | 0.89 | 0.04 | -0.17 | 0.35 | 0.41 | 0.17 |
| ***155*** | 0.58 | 0.19 | 0.89 | 0.04 | 0.09 | 0.31 | 0.49 | 0.03 |
| ***166*** | 0.66 | 0.21 | 0.90 | 0.02 | 0.33 | 0.23 | 0.43 | 0.06 |
| ***233*** | 0.43 | 0.06 | 0.51 | 0.04 | -0.31 | 0.08 | 0.34 | 0.06 |
| ***244*** | -0.01 | 0.23 | 0.47 | 0.18 | -0.23 | 0.15 | -0.02 | 0.21 |
| ***334*** | 0.28 | 0.07 | -0.04 | 0.07 | -0.08 | 0.37 | 0.11 | 0.14 |
| ***223*** | 0.86 | 0.05 | 0.72 | 0.05 | -0.22 | 0.14 | 0.40 | 0.06 |
| ***224*** | 0.76 | 0.23 | 0.85 | 0.05 | -0.20 | 0.17 | 0.11 | 0.33 |
| ***225*** | 0.76 | 0.25 | 0.83 | 0.06 | 0.05 | 0.40 | 0.35 | 0.14 |
| ***226*** | 0.76 | 0.21 | 0.89 | 0.01 | 0.22 | 0.36 | 0.43 | 0.01 |
| ***134*** | 0.73 | 0.23 | 0.88 | 0.06 | -0.13 | 0.27 | 0.51 | 0.09 |
| ***135*** | 0.72 | 0.20 | 0.87 | 0.07 | 0.07 | 0.28 | 0.53 | 0.07 |
| ***136*** | 0.71 | 0.18 | 0.88 | 0.05 | 0.24 | 0.26 | 0.57 | 0.05 |
| ***145*** | 0.57 | 0.16 | 0.90 | 0.02 | -0.06 | 0.32 | 0.46 | 0.15 |
| ***234*** | 0.31 | 0.13 | 0.46 | 0.06 | -0.21 | 0.12 | 0.23 | 0.09 |
| ***235*** | 0.41 | 0.08 | 0.51 | 0.05 | 0.08 | 0.29 | 0.31 | 0.05 |

**Table S7**. The melting temperature (*T_m_*) for each sequence, the average of *T_m_* values (‾*T_m_*) and *T_m_* variance (δ) for every group in 100 mM KCl. Data were collected from Figure S16, Tm deduced from the first derivative of the melting curves.

| Name | *T_m_* | *‾T_m_* for group | *T_m_* δ for group | Name | *T_m_* | *‾T_m_* for group | *T_m_* δ for group |
| --- | --- | --- | --- | --- | --- | --- | --- |
| 133 | 72.4 | 70.8 | 2.95 | DT2-133 | 72.4 | 71.3 | 2.35 |
| 313 | 67.4 |  |  | DT2-313 | 68.6 |  |  |
| 331 | 72.6 |  |  | DT2-331 | 72.9 |  |  |
| 144 | 67.5 | 64.8 | 2.39 | DT2-144 | 63.5 | 62.7 | 1.74 |
| 414 | 63.7 |  |  | DT2-414 | 60.7 |  |  |
| 441 | 63.1 |  |  | DT2-441 | 63.9 |  |  |
| 155 | 61.6 | 57.8 | 3.54 | DT2-155 | 56.4 | 55.3 | 1.10 |
| 515 | 54.6 |  |  | DT2-515 | 54.2 |  |  |
| 551 | 57.2 |  |  | DT2-551 | 55.2 |  |  |
| 166 | 55.5 | 54.9 | 2.03 | DT2-166 | 52.4 | 51.2 | 1.16 |
| 616 | 52.6 |  |  | DT2-616 | 51.0 |  |  |
| 661 | 56.5 |  |  | DT2-661 | 50.1 |  |  |
| 233 | 66.9 | 67.0 | 2.45 | DT2-233 | 63.0 | 63.4 | 1.00 |
| 323 | 64.6 |  |  | DT2-323 | 62.6 |  |  |
| 332 | 69.5 |  |  | DT2-332 | 64.5 |  |  |
| 244 | 65.3 | 63.6 | 2.89 | DT2-244 | 54.7 | 55.5 | 0.85 |
| 424 | 60.3 |  |  | DT2-424 | 56.4 |  |  |
| 442 | 65.3 |  |  | DT2-442 | 55.5 |  |  |
| 134 | 67.0 | 68.2 | 3.11 | DT2-134 | 66.5 | 66.4 | 1.19 |
| 143 | 74.2 |  |  | DT2-143 | 67.6 |  |  |
| 314 | 66.2 |  |  | DT2-314 | 64.6 |  |  |
| 341 | 68.9 |  |  | DT2-341 | 67.6 |  |  |
| 413 | 66.4 |  |  | DT2-413 | 65.5 |  |  |
| 431 | 66.4 |  |  | DT2-431 | 66.8 |  |  |
| 135 | 63.0 | 63.9 | 3.52 | DT2-135 | 61.8 | 62.2 | 0.75 |
| 153 | 70.9 |  |  | DT2-153 | 62.9 |  |  |
| 315 | 61.5 |  |  | DT2-315 | 62.5 |  |  |
| 351 | 64.0 |  |  | DT2-351 | 63.1 |  |  |
| 513 | 62.1 |  |  | DT2-513 | 61.1 |  |  |
| 531 | 62.1 |  |  | DT2-531 | 62.0 |  |  |
| 136 | 62.9 | 61.8 | 3.31 | DT2-136 | 59.8 | 59.3 | 0.50 |
| 163 | 67.4 |  |  | DT2-163 | 58.8 |  |  |
| 316 | 58.7 |  |  | DT2-316 | 58.6 |  |  |
| 361 | 62.6 |  |  | DT2-361 | 59.8 |  |  |
| 613 | 60.5 |  |  | DT2-613 | 59.4 |  |  |
| 631 | 58.6 |  |  | DT2-631 | 59.3 |  |  |
|  |  |  |  |  |  |  |  |
| **Table S7**. Continued | | |  |  |  |  |  |
| 145 | 61.6 | 60.1 | 2.42 | DT2-145 | 57.7 | 57.2 | 0.54 |
| 154 | 64.3 |  |  | DT2-154 | 57.0 |  |  |
| 415 | 58.3 |  |  | DT2-415 | 57.3 |  |  |
| 451 | 58.0 |  |  | DT2-451 | 57.8 |  |  |
| 514 | 59.2 |  |  | DT2-514 | 57.2 |  |  |
| 541 | 59.1 |  |  | DT2-541 | 56.3 |  |  |
| 234 | 66.4 | 66.1 | 4.99 | DT2-234 | 57.7 | 59.0 | 1.68 |
| 243 | 74.2 |  |  | DT2-243 | 59.3 |  |  |
| 324 | 61.1 |  |  | DT2-324 | 59.5 |  |  |
| 342 | 69.0 |  |  | DT2-342 | 61.3 |  |  |
| 423 | 61.3 |  |  | DT2-423 | 56.5 |  |  |
| 432 | 64.6 |  |  | DT2-432 | 59.7 |  |  |
| 235 | 63.1 | 62.0 | 6.04 | DT2-235 | 52.9 | 53.6 | 1.53 |
| 253 | 71.5 |  |  | DT2-253 | 55.0 |  |  |
| 325 | 57.2 |  |  | DT2-325 | 52.5 |  |  |
| 352 | 65.3 |  |  | DT2-352 | 55.2 |  |  |
| 523 | 54.6 |  |  | DT2-523 | 51.4 |  |  |
| 532 | 60.4 |  |  | DT2-532 | 54.4 |  |  |
| 334 | 64.4 | 66.1 | 2.50 | DT2-334 | 55.6 | 55.5 | 0.23 |
| 343 | 69.0 |  |  | DT2-343 | 55.6 |  |  |
| 433 | 65.0 |  |  | DT2-433 | 55.2 |  |  |
| 223 | 73.2 | 71.7 | 3.28 | DT2-223 | 69.8 | 69.1 | 2.72 |
| 232 | 67.9 |  |  | DT2-232 | 66.1 |  |  |
| 322 | 73.9 |  |  | DT2-322 | 71.4 |  |  |
| 224 | 64.0 | 65.6 | 1.89 | DT2-224 | 61.8 | 62.8 | 1.05 |
| 242 | 67.7 |  |  | DT2-242 | 63.9 |  |  |
| 422 | 65.2 |  |  | DT2-422 | 62.8 |  |  |
| 225 | 60.9 | 62.2 | 1.86 | DT2-225 | 60.0 | 60.0 | 0.60 |
| 252 | 64.3 |  |  | DT2-252 | 59.4 |  |  |
| 522 | 61.3 |  |  | DT2-522 | 60.6 |  |  |
| 226 | 56.5 | 58.3 | 1.53 | DT2-226 | 57.6 | 58.1 | 0.93 |
| 262 | 59.1 |  |  | DT2-262 | 59.2 |  |  |
| 622 | 59.2 |  |  | DT2-622 | 57.6 |  |  |

**Table S8**. Number of different GpG steps and 5ʹ-terminal OH···N3 H-bonds (HB) in each G4 model (see Figure S20) and its MM-PBSA relative free energy Δ*G*. Each model can be thought of as if composed of eight individual GpG steps and, if applicable, 5ʹ-terminal *syn*-G specific 5ʹOH···N3 H-bonds.

| **Model** | **GpG step** | | | | **5ʹ-terminal H-bond in *syn*-G** | | **Δ*G*** |
| --- | --- | --- | --- | --- | --- | --- | --- |
|  | *anti-anti* | *syn-anti* | *syn-syn* | *anti-syn* | 5ʹ-HB-*syn*-*anti ^a^* | 5ʹ-HB-*syn*-*syn ^b^* | (kcal/mol) |
| **1a** | 8 | 0 | 0 | 0 | 0 | 0 | 0 ^c^ |
| **1b** | 4 | 4 | 0 | 0 | 4 | 0 | -33.3 |
| **1c** | 3 | 4 | 1 | 0 | 3 | 1 | -26.9 |
| **1d** | 0 | 4 | 0 | 4 | 2 | 0 | -17.8 |
| **2a** | 8 | 0 | 0 | 0 | 0 | 0 | -0.2 |
| **2b** | 4 | 4 | 0 | 0 | 3 | 0 | -27.0 |
| **3a** | 8 | 0 | 0 | 0 | 0 | 0 | +1.9 |
| **3b** | 4 | 4 | 0 | 0 | 0 | 0 | -3.8 |
| **3c** | 3 | 4 | 1 | 0 | 0 | 0 | -1.8 |
| **3d** | 0 | 4 | 0 | 4 | 0 | 0 | -2.0 |

^a^ The H-bond in 5ʹ-terminal G is in a *syn*-*anti* GpG step.

^b^ The H-bond in 5ʹ-terminal G is in a *syn*-*syn* GpG step.

^c^ Model **1a** is used as a reference for all the other G-stems.

**Table S9**. Relative free-energy contributions of the four possible types of GpG steps and stabilizing free-energy effect of the 5ʹ-terminal 5ʹOH···N3 H-bonds (the last two rows) estimated from the MM-PBSA Δ*G* calculations in Table S8. The values were derived by the ordinary least squares method from the overall MM-PBSA data, in which Δ*G* of the GpG step type or H-bond was taken as a variable and the total number of its occurrences in a given model as a coefficient. The resultant coefficient of determination R^2^ of the fit is equal to 0.998.

| **GpG step or 5ʹ-syn-G H-bond** | **Δ*G* (kcal/mol)** |
| --- | --- |
| *anti*-*anti ^a^* | 0 ^a^ |
| *syn*-*anti ^b^* | -1.1 |
| *syn*-*syn ^b^* | +2.0 |
| *anti*-*syn* | +0.4 |
| 5ʹ-HB-*syn*-*anti* | -7.6 |
| 5ʹ-HB-*syn*-*syn* | -2.5 |

^a^ *Anti-anti* step is used as a reference for all the other steps.

^b^ To obtain Δ*G* of the corresponding 5ʹ-terminal step, it is necessary to add the term of the corresponding 5ʹ-terminal H-bond.

**Interpretation of the data in Tables S8 and S9**

The MM-PBSA free-energy calculations clearly show that adding a 5'-end flanking sequence to a 5'-end *anti*-G has no effect on stability of the G-stem, while the same addition to a 5'-end *syn*-G leads to a destabilization (Table S8). The data would predict that the free-energy effect of one 5'O-H···N3 H-bond should be around -7 kcal/mol (Table S9). However, the MM-PBSA method, while providing correct relative trends, is well-known to over-estimate the free-energy differences (S18). Thus, an alternative and probably more realistic estimate can be obtained based on analysis of population of the H-bond in the simulations (Table S10, Figure S21). Considering all data, we suggest that the stabilizing free-energy effect associated with the 5'O-H···N3 H-bond is about -1 to -2 kcal/mol. This stabilizing contribution is lost upon adding any flanking sequence to any 5'-end *syn*-G.

**Table S10**. Population analysis of the terminal 5ʹ-OH···N3 hydrogen-bond in models **1b**, **1c** and **1d** (see **Figures S20** and **S21**).

| **Model** | **nucleotide** | **H-bond pop. (%)** | **Δ*G* (kcal/mol)** |
| --- | --- | --- | --- |
| **1b** | G1 | 88.5 | -1.2 |
|  | G4 | 90.7 | -1.4 |
|  | G7 | 88.0 | -1.2 |
|  | G10 | 89.7 | -1.3 |
| **1c** | G1 | 89.1 | -1.2 |
|  | G4 | 90.2 | -1.3 |
|  | G7 ^a^ | 61.9 | -0.3 |
|  | G10 | 94.7 | -1.7 |
| **1d** | G4 | 92.2 | -1.5 |
|  | G10 | 89.8 | -1.3 |

^a^ 5ʹ-G in a *syn*-*syn* step; all the other Gs in a *syn*-*anti* step.

**Table S11**. Population analysis of the internal base(N2)···phosphate interaction in models **2b**, **3b**, **3c** and **3d** (see **Figures S20** and **S22**).

| **Model** | **nucleotide** | **H-bond pop. (%)** | **Δ*G* (kcal/mol)** |
| --- | --- | --- | --- |
| **2b** | G2 | 10.4 | +1.3 |
| **3b** | G2 | 74.5 | -0.6 |
|  | G6 | 43.0 | +0.2 |
|  | G10 | 2.9 | +2.1 |
|  | G14 | 50.8 | 0.0 |
| **3c** | G2 | 0.1 | +4.1 |
|  | G6 | 43.2 | 0.2 |
|  | G10 ^a^ | 0.1 | +4.1 |
|  | G14 | 78.5 | -0.8 |
| **3d** | G6 | 0.3 | +3.6 |
|  | G14 | 61.6 | -0.3 |

^a^ G in a *syn*-*syn* step; all the other Gs in a *syn*-*anti* step.

**Table S12**. Population analysis of the internal base(N2)···phosphate interaction in *syn*-G’s in selected human telomeric G-quadruplex models as observed in 10 µs-long MD-simulation trajectories taken from our preceding studies.

| **Model** | **nucleotide** | **H-bond pop. (%)** | **Δ*G* (kcal/mol)** |
| --- | --- | --- | --- |
| **2MBJ** (S19) | G4 *^a, b^* | 0.6 | +1.3 |
|  | G5 | 0 | >5.5 |
|  | G10 *^b, c^* | 83.1 | -1.0 |
|  | G16 *^b^* | 0 | >5.5 |
|  | G22 *^a, b^* | 0.1 | +4.4 |
|  | G23 *^b^* | 0.2 | +3.9 |
| **2JPZ** (S28) | G4 | 39.4 | +0.3 |
|  | G10 *^a^* | 0.0 | +4.8 |
|  | G11 | 0 | >5.5 |
|  | G16 *^c^* | 0.6 | +3.1 |
|  | G22 | 0.3 | +3.6 |
| **2HY9** (S23) | G4 *^b^* | 5.8 | +1.7 |
|  | G10 | 57.9 | -0.2 |
|  | G16 *^a^* | 2.0 | +2.3 |
|  | G17 | 0 | >5.5 |
|  | G22 *^c^* | 71.8 | -0.6 |
| **143D** (S2) | G3 | 0 | >5.5 |
|  | G8 | 0.1 | +4.4 |
|  | G10 | 3.1 | +2.1 |
|  | G15 | 0 | >5.5 |
|  | G20 *^b, c^* | 66.5 | -0.4 |
|  | G22 *^b^* | 0.1 | +4.3 |
| **2KF8** (S20) | G1 *^b, d^* | 92.5 | -1.5 |
|  | G7 *^b^* | 0.2 | +3.6 |
|  | G14 | 1.3 | +2.6 |
|  | G19 *^b, c^* | 65.5 | -0.4 |

^a^ G in a *syn*-*syn* step; all the other Gs in a *syn*-*anti* step.

^b^ internal H-bond present in the NMR model (at least one, if several are published). In absence of footnote “b”, the H-bond is absent.

^c^ first G after TTA loop spanning the narrow groove

^d^ this line shows 5ʹ-end *syn*-specific 5ʹ-OH···N3 H-bond, the only one in the whole Table

**Detailed explanation of the significance and interpretation of the free-energy computations**

Detailed explanation how the stabilizing effect of the 5ʹ-terminal 5ʹ-OH···N3 H-bond can be derived from the relative free energies of the molecules depicted in **Figure S20** as summarized in **Table S8**.

First, structures **1a**, **2a** and **3a** have identical free energies (within the limits of sampling). It means that addition of a flanking T to the 5ʹ-end having *anti* G does not have any effect on the free energy of the G-stem. On the other hand, comparison of MM-PBSA energies of structures **1b**, **2b** and **3b** reveals a major destabilizing effect of adding the flanking T to the *syn* 5ʹ-terminal G. Namely, the free-energy difference between structures **3b** and **1b** is 29.5 kcal/mol, which means free-energy penalty of 7.4 kcal/mol per one added flanking T. This is consistent with the 6.3 kcal/mol difference between structures **2b** and **1b**. Difference around 1 kcal/mol between the two calculations can be considered to be within the overall uncertainty of the MM-PBSA method.

When considering the stem in the hybrid topology **3**, we see energy difference of 25.1 kcal/mol between **3c** and **1c**, which means a free-energy penalty of 6.3 kcal/mol per flanking T added to one *syn* 5ʹ-terminal G. Finally, in case of structures **3d** and **1d**, the free-energy difference is 15.8 kcal/mol, leading to 7.9 kcal/mol difference per one realization of added flanking T to 5ʹ*-*terminal G (assuming that the flanking Ts added to the two terminal *anti* Gs have no effect on the free energies).

The computations thus predict that addition of flanking segment to the 5ʹ-terminus in *anti* has no effect on the free energy of the stem while addition of flanking T to 5ʹ-terminus of G in *syn* is universally (for all three folds) destabilizing in the range of 6.3 to 7.9 kcal/mol. These numbers deserve two comments. First, the variation of the numbers can be considered to be within the limits of the noise of the MM-PBSA method. Second, the free-energy effect is evidently over-estimated. This, however, is a common feature of the MM-PBSA data (S31), which typically need to be scaled down. It is more realistic to expect that formation of one strong H-bond in aqueous environment can bring 1-2 kcal/mol stabilization. Therefore, as another analysis, we estimated free energies of the 5ʹ-OH···N3 H-bond from its population in simulations (**Table S10**). Although this computation does not directly provide contribution of this H-bond to the stem stability, it is fair to assume that it can be used as its qualified estimate. The **Table S10** predicts that one *syn*-specific 5ʹ-OH…N3 H-bond increases stability of the stem by 1.2-1.7 kcal/mol. The only outlier is the third H-bond of the hybrid topology, where the stabilizing effect is estimated to be 0.3 kcal/mol. Interestingly, this outlier corresponds to the only 5ʹ-terminal GpG step with *syn*-*syn* configuration while in all other cases the studied 5ʹ-terminal GpG steps have *syn*-*anti* arrangement.

The latter result may indicate that the destabilizing effect of the 5ʹ-flanking sequences is larger for the 5ʹ-terminal GpG *syn*-*anti* steps than for GpG *syn*-*syn* steps. This is also consistent with the MM-PBSA data, since the energy gain per one 5ʹ-OH···N3 H-bond is smaller for the **3c** structure than for the **4c** and **2c** structures. However, we caution that we have only one occurrence of the GpG *syn*-*syn* step in our dataset and the difference may be affected by the noise of the computations and various approximations. To prove larger effect of the 5ʹ-OH···N3 H-bond in GpG *syn*-*anti* compared to the GpG *syn*-*syn* steps may require further investigations. Nevertheless, on quantitative note, the computations indicate that the effect of 5'OH···N3 H-bond might be somewhat stronger for G4 folds that start with 5'-*syn*-*anti* GpG step (such as, *e.g.* the 2HY9 or 2JPZ hybrid folds of human telomeric G4 without flanking nucleotides) compared to folds starting with 5'-*syn*-*syn* GpG step (such as the 2MBJ antiparallel fold of human telomeric G4 without flanking nucleotides).

In the past, MM-PBSA calculations on minimal two-quartet stems have been used to suggest different stabilities of GpG dinucleotides from which the G-stems are constructed (S32,S33). These predictions were many times successfully used to rationalize observed G4 topologies and their *syn/anti* patterns. Thus, we have also used our new MM-PBSA data (obtained for larger stems, with a longer time-scale and significantly improved OL15 force field) to construct an analogous (for more details see the earlier works) prediction, which is summarized in the **Table S9**. Although the results are numerically slightly different, we confirm the earlier-predicted MM-PBSA stability order of GpG steps *syn*-*anti* > *anti*-*anti* > *anti*-*syn* > *syn*-*syn*. We note that while three-quartet stems are more complete model systems than two-quartet stems, the MM-PBSA data for three-quartet stems may be noisier due to occurrence of substates and imperfect description of the inter-cation interaction inside the stem, as discussed elsewhere (S18). It should also be noted that the higher stability of the *syn*-*anti* step compared to *anti*-*anti* step seems at first sight somewhat counter-intuitive. However, it has been shown using advanced electronic structure (quantum-chemical, QM) calculations (S33) that the AMBER force field underestimates relative stability of the *anti*-*anti* GpG step compared to the remaining three arrangements. Thus, a correction of the MM-PBSA-predicted free-energy order based on QM calculations of potential energies has been suggested (S33). It leads to the following final stability order: *anti*-*anti* ≥ *syn*-*anti* > *anti*-*syn* > *syn*-*syn* (S33), i.e., the *anti*-*anti* and *syn*-*anti* steps should be considered as equally stable. It dictates the intrinsic *syn/anti* preferences of different G4 folds based on the composition of the steps. We did not attempt calculation of such QM correction in the present work as it is not necessary to derive the free-energy contribution from the 5ʹ-terminal H-bond.

**Analysis of the potential guanine amino group**···**phosphate H-bond.**

We have carefully analysed also the potential role of the *syn* guanine amino-group···phosphate intra-nucleotide H-bond that could be present upon adding the 5’-end flanking sequence. This interaction could potentially compensate for the loss of the 5ʹ-OH···N3 terminal H-bond. However, the amino-group···phosphate interaction seems to be considerably less important. Close amino···phosphate contacts form to certain extent in the simulations of the tetrameric models, but their population in the overall simulation ensemble is around 30% (**Table S11**, **Figure S22**). In our simulations we observed a few dynamically exchanging substates of the flanking thymines, and the “*syn*-G” close contacts between the guanine amino group and the phosphate group occur only for some of them, i.e., only in specific conformational substates. The populations of these substates vary across our models/simulations, as the thymines can interact not only with the G-stem, but also with other thymines, whose relative position depends on a given model. Although the individual trajectories are not fully converged, we consider our aggregate trajectory data fairly representative. Population analysis of the interaction in our older simulations of the human telomeric G4s also suggest that the H-bond is not decisively stabilizing the folds (Table S12). The only systematic exception, apart from 2JPZ, is the H-bond formation in the first *syn*-G following the lateral TTA loop that spans the narrow groove. Formation of this bond has also been observed in previous simulations of G-hairpins (S25). Obviously, since this particular type of base···phosphate interaction requires a loop and a preceding G-tract, it cannot compensate for the loss of 5ʹ-OH···N3 terminal H-bond by principle. The NMR models of the structures also suggest formation of the base···phosphate interaction at this site (Table S12). Some NMR models suggest formation of the base···phosphate interaction also at other *syn*-G sites, typically along the narrow groove, but we think that this can be to a certain extent an artifact attributed to the refinement procedure. Our belief is corroborated by the fact that at a particular *syn*-G site, the bond is usually not present in all the published models. The base···phosphate interaction can sometimes be populated in a specific loop and/or flanking nucleotide substate. In other words, the interaction is not universally present and likely not formed permanently.

Since this interaction was originally suggested for Z-DNA, we have also inspected a few Z-DNA crystal structures and found out that they do not indicate a substantial population of such H-bonds. *Syn*-G in Z-DNA rather prefers a *water-mediated* (bridged) interaction of the amino group either to the 3ʹ-phosphate or both 3ʹ- and 5ʹ-phosphate. It is to be noted that the interaction was suggested based on model quantum-chemical (QM) calculations on isolated G-nucleotide and involved a substantial pyramidalization of the amino group to be fully developed (S26). Pyramidalization of nucleobase amino groups has been discovered and extensively studied by QM calculations in isolation as well as in diverse contexts (S34,S35). Amino groups of isolated nucleic acid bases in gas phase are indeed nonplanar, due to a partial sp^3^ hybridization of the amino group nitrogens, allowing to potentially form diverse out-of-plane H-bonds. However, the partial sp^3^ hybridization is abolished when the amino group is involved in planar base pairing, due to a change of the electronic structure of the amino group (S35). Thus, formation of the amino-group···phosphate H-bond would likely be electronically in conflict (anti-cooperative) with the involvement of the guanine in the G-quartet, reducing to our opinion the energy gains by formation of the amino-group···phosphate interactions. In summary, our data do not suggest a significant stabilizing role of a *syn*-specific internal base···phosphate interaction formed between the phosphate group and amino group of G (**Table S11**). The interaction seems to be formed to certain extent but only intermittently with rather low population. Thus, we suggest that formation of this interaction does not energetically compensate for the loss of the 5ʹ-terminal 5ʹ-OH···N3 H-bond upon addition of a 5ʹ flanking sequence.


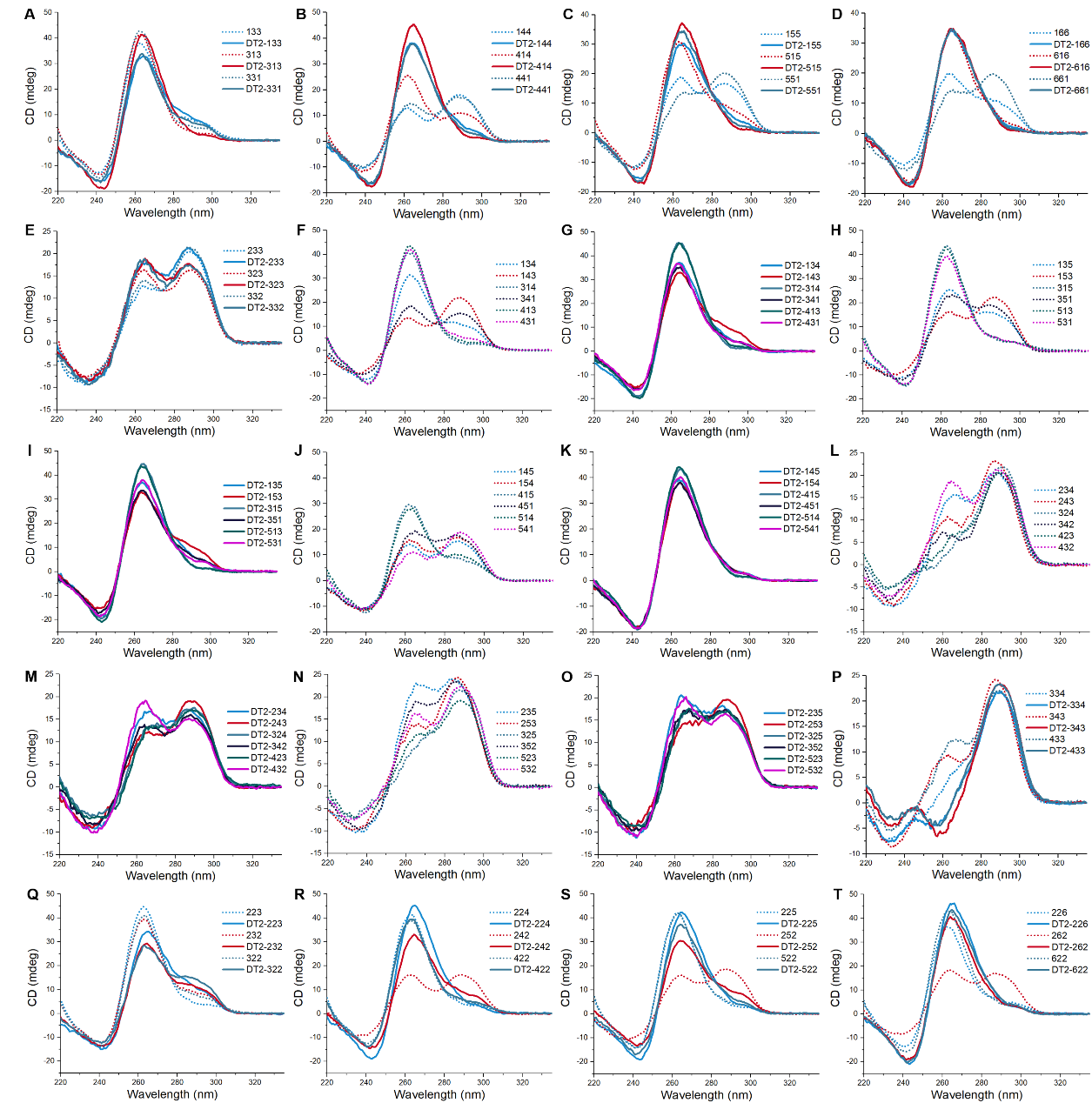


**Figure S1.** CD spectra of (**A**) ***133*** group, (**B**) ***144*** group, (**C**) ***155*** group, (**D**) ***166*** group, (**E**) ***233*** group, (**F, G**) ***134*** group, (**H, I**) ***135*** group, (**J, K**) ***145*** group, (**L, M**) ***234*** group, (**N, O**) ***235*** group, (**P**) ***334*** group, (**Q**) ***223*** group, (**R**) ***224*** group, (**S**) ***225*** group, and (**T**) ***226*** group without or with both terminal 2dTs. CD experiments were performed at 20 ^o^C by using 5 μM strand concentrations in 100 mM KCl (pH 7.2). It should be noted that ***136*** group and ***244*** group are shown in the main manuscript, so not shown here.


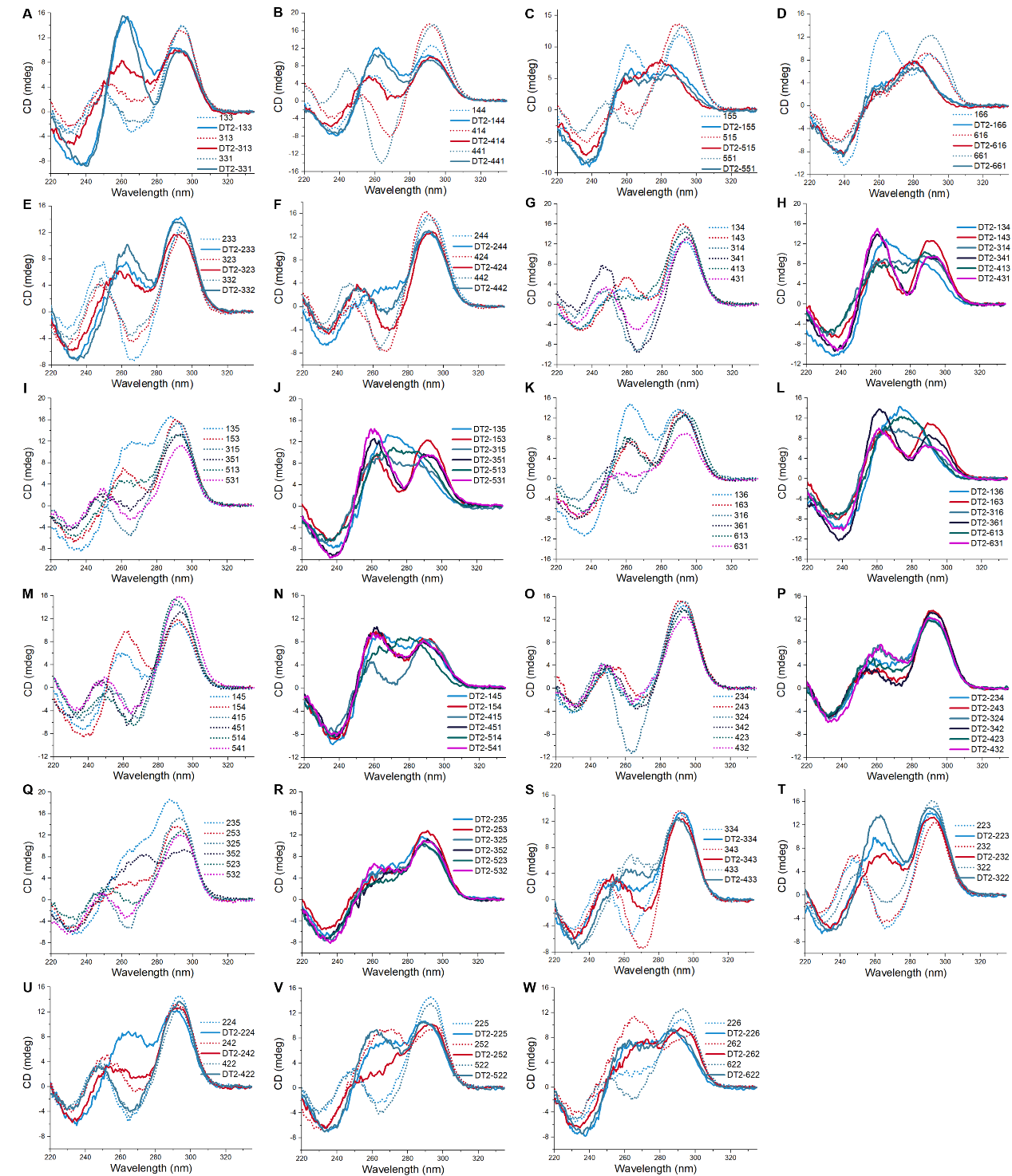


**Figure S2.** CD spectra of (**A**) ***133*** group, (**B**) ***144*** group, (**C**) ***155*** group, (**D**) ***166*** group, (**E**) ***233*** group, (**F**) ***244*** group, (**G, H**) ***134*** group, (**I, J**) ***135*** group, (**K, L**) ***136*** group, (**M, N**) ***145*** group, (**O, P**) ***234*** group, (**Q, R**) ***235*** group, (**S**) ***334*** group, (**T**) ***223*** group, (**U**) ***224*** group, (**V**) ***225*** group, and (**W**) ***226*** group without and with both terminal 2dTs. CD experiments were carried out at 20 ^o^C by using 5 μM strand concentrations in 100 mM NaCl (pH 7.2).


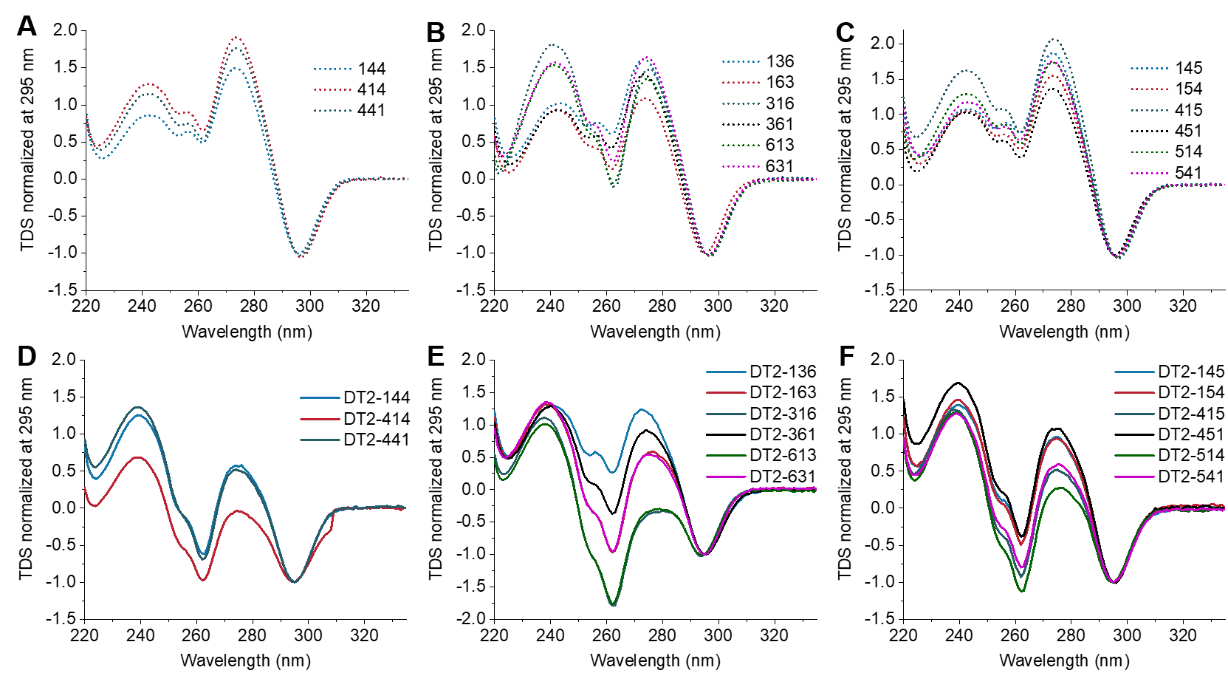


**Figure S3**. TDS spectra for (**A**) ***144*** group, (**B**) ***136*** group, (**C**) ***145*** group, (**D**) DT2*-****144*** group, (**E**) DT2*-****136*** group, and (**F**) DT2*-****145*** group. Experiments were performed at 4 and 90 ^o^C, with 100 mM KCl.


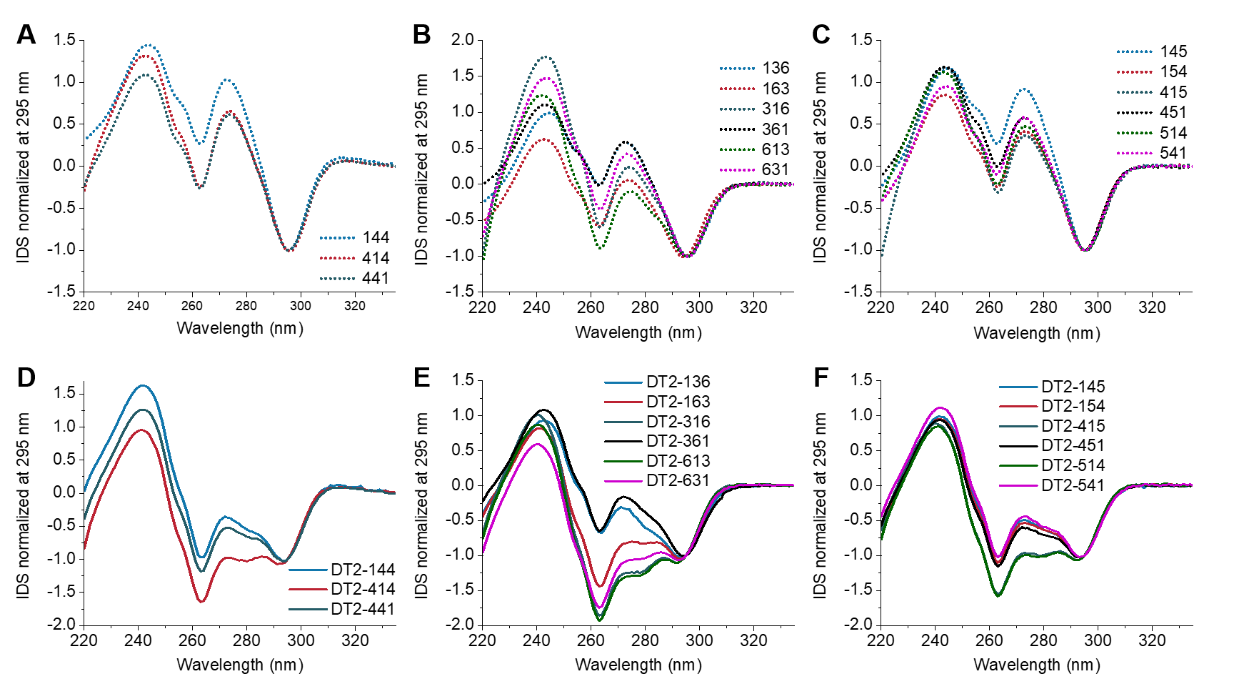


**Figure S4**. IDS spectra for (**A**) ***144*** group, (**B**) ***136*** group, (**C**) ***145*** group, (**D**) DT2*-****144*** group, (**E**) DT2*-****136*** group, and (**F**) DT2*-****145*** group. Experiments were performed in the absence or presence of 100 mM KCl.


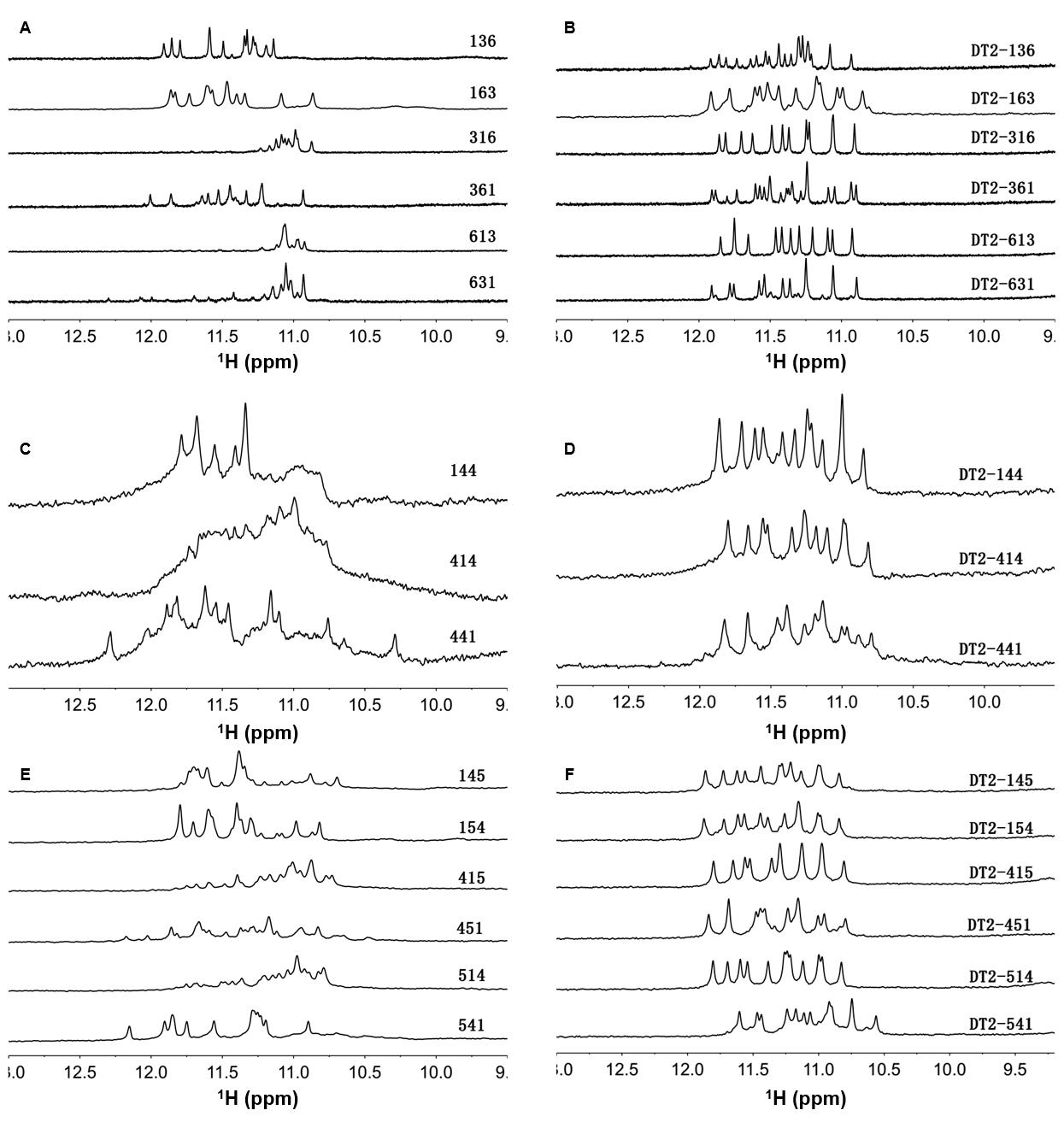


**Figure S5**. ^1^H NMR spectra of imino protons of G4s from (**A**) ***136*** group, (**B**) DT2*-****136*** group, (**C**) ***144*** group, (**D**) DT2*-****144*** group, (**E**) ***145*** group, (**F**) DT2*-****145*** group. NMR experiments were performed using 100 μM strand concentrations in 10 mM KPi (pH 6.6).

**
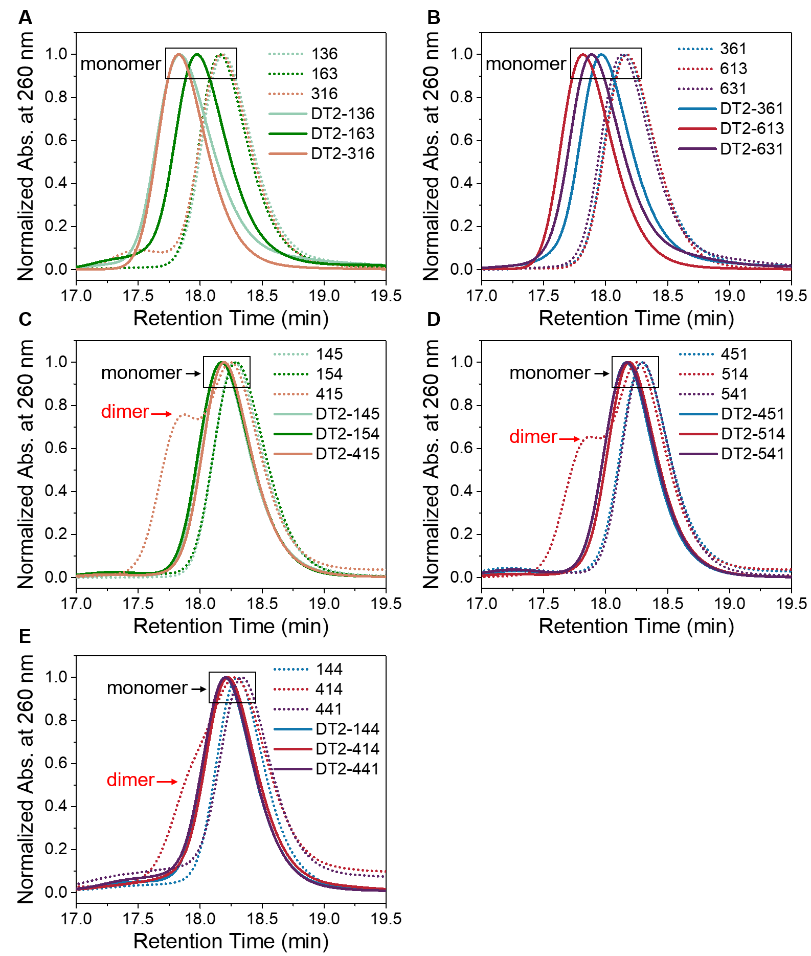
**

**Figure S6**. SE-HPLC profiles of (**A**, **B**) ***136*** group, (**C**, **D**) ***145*** group and (**E**) ***144*** group without or with both terminal 2dTs, in 100 mM KCl.

**Size-Exclusion HPLC**

Size-exclusion HPLC was performed essentially as described previously (S36). Aliquots of 20 µL of solutions of 10 µM oligonucleotide were injected onto a Waters Alliance e2695 HPLC system equipped with a Thermo Acclaim SEC-300 column (4.6 × 300 mm, 5-µm hydrophilic polymethacrylate resin spherical particles, 300 Å pore size). Unless otherwise stated, elution was performed at 0.15 mL/min in 10 mM Tris-HCl (pH 7.0) buffer, supplemented with 100 mM KCl. The column and sample temperatures were 20 °C.

From the spectra, almost all of these sequences formed monomer, except for 415, 514, and 414 contain small fraction of dimer.


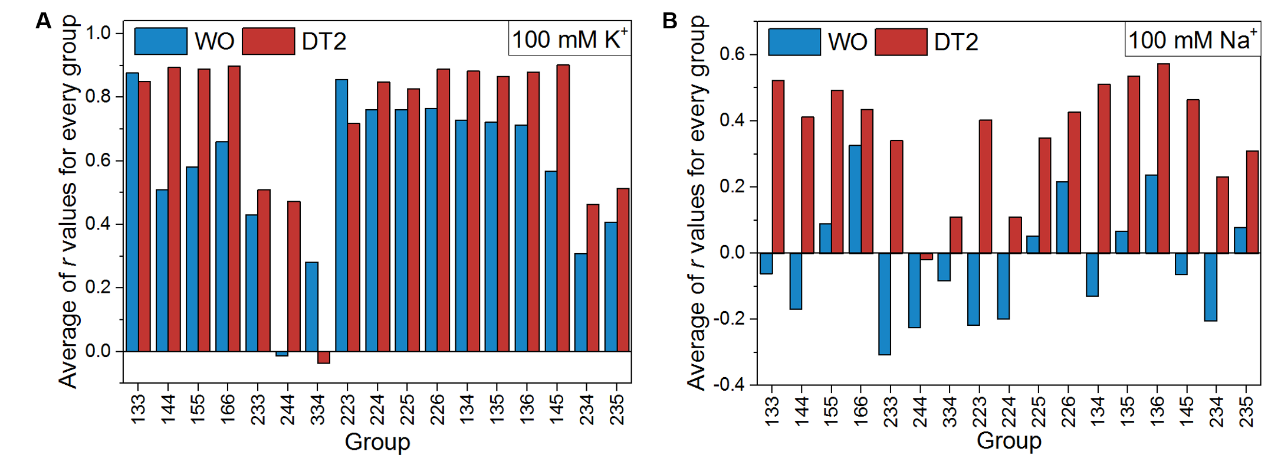


**Figure S7**. The average of *r* values, without (WO) and with (DT2) addition of 2dTs at both terminals, for every group in 100 mM (**A**) KCl and (**B**) NaCl.


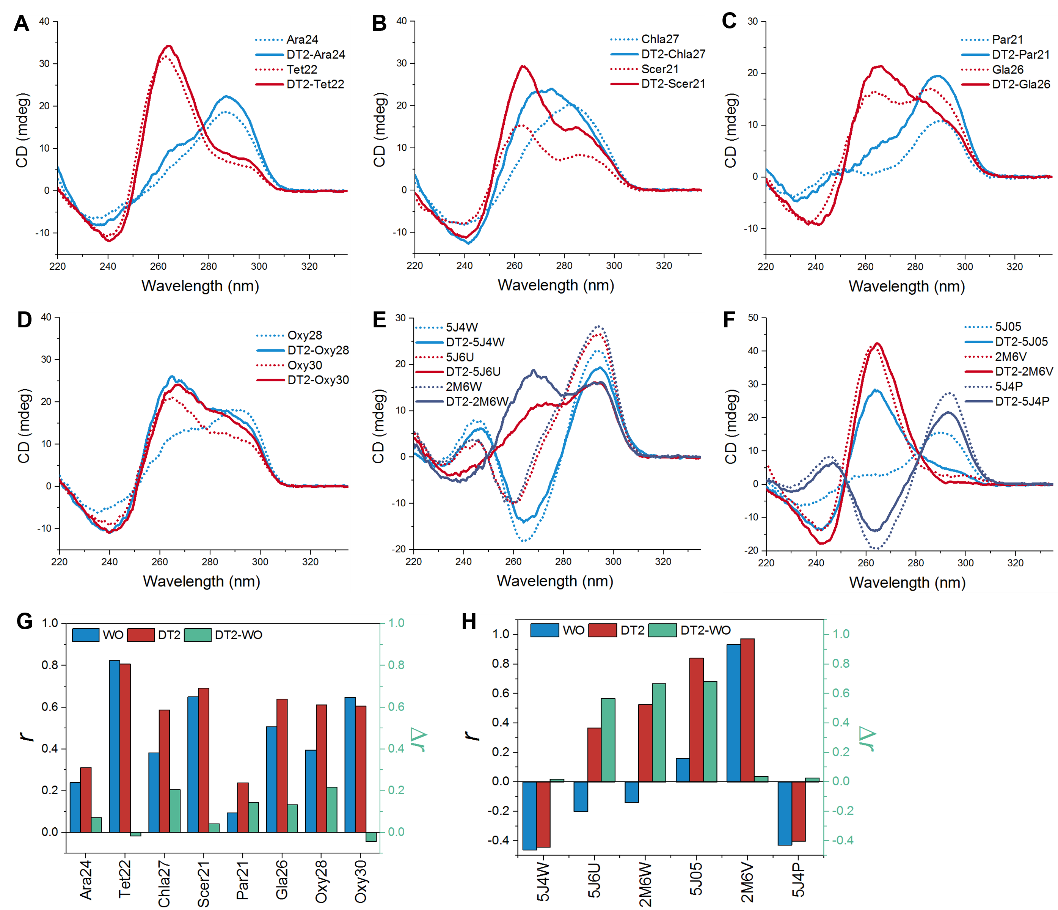


**Figure S8**. Natural and previously studied sequences gathered from references (S29,S30). The CD spectra (**A-F**) and *r* values (**G-H**) of sequences without (WO) and with (DT2) 2dTs at both terminals. Experiments were performed in 100 mM KCl.


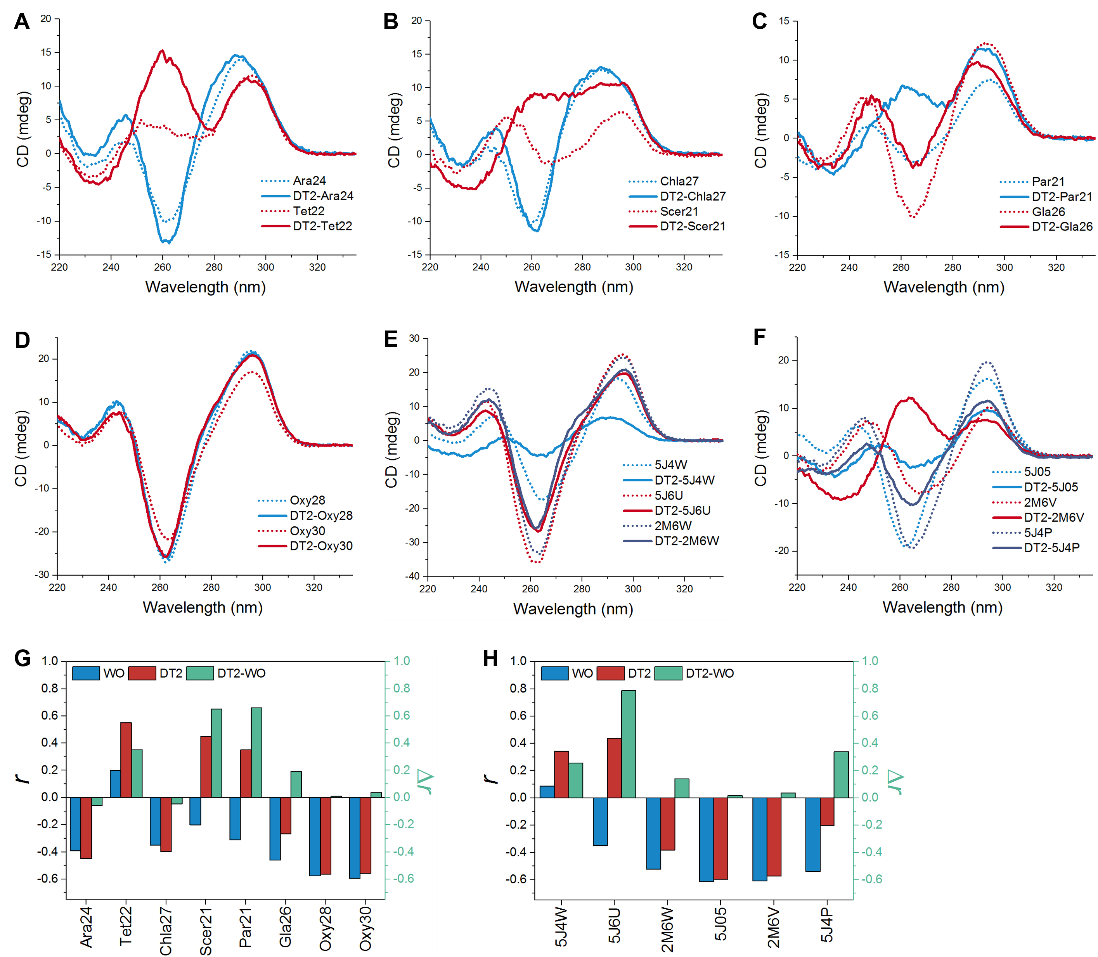


**Figure S9**. Natural and previously studied sequences gathered from references (S29,S30). The CD spectra (**A-F**) and *r* values (**G-H**) of sequences without (WO) and with (DT2) 2dTs at both terminals. Experiments were performed in 100 mM NaCl.


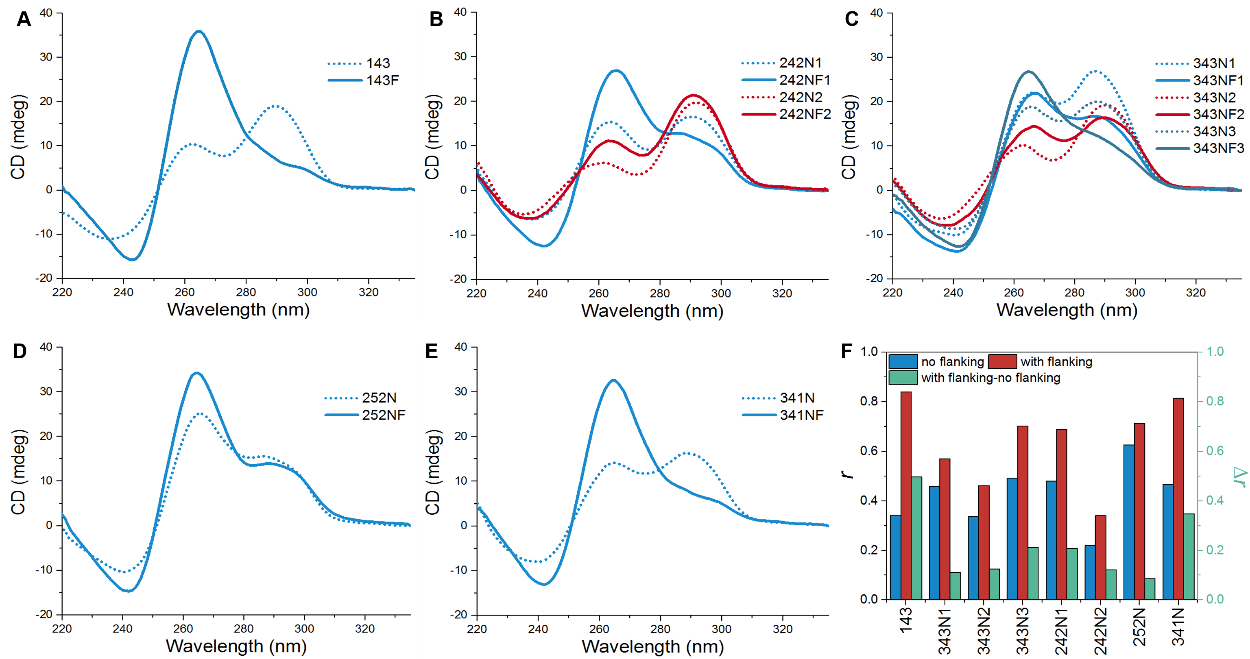


**Figure S10**. Natural sequences searched from BLAST. The CD spectra (**A-E**), *r* values (**F**), and their difference (Δ*r*, **F**) of sequences without and with natural flanking nucleotides at both terminals. All of them got higher *r* values due to the addition of natural flanking sequences. Experiments were performed in 100 mM KCl.


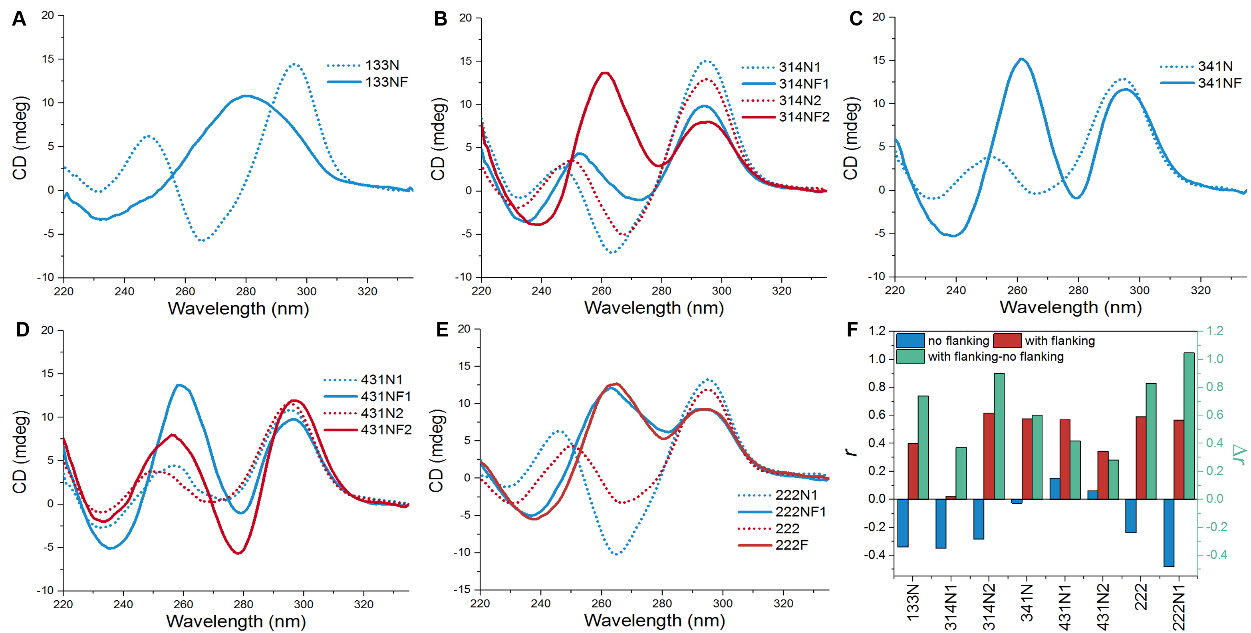


**Figure S11**. Natural sequences searched from BLAST. The CD spectra (**A-E**), *r* values (**F**), and their difference (Δ*r*, **F**) of sequences without and with natural flanking nucleotides at both terminals. All of them got higher *r* values due to the addition of natural flanking sequences. Experiments were performed in 100 mM NaCl.


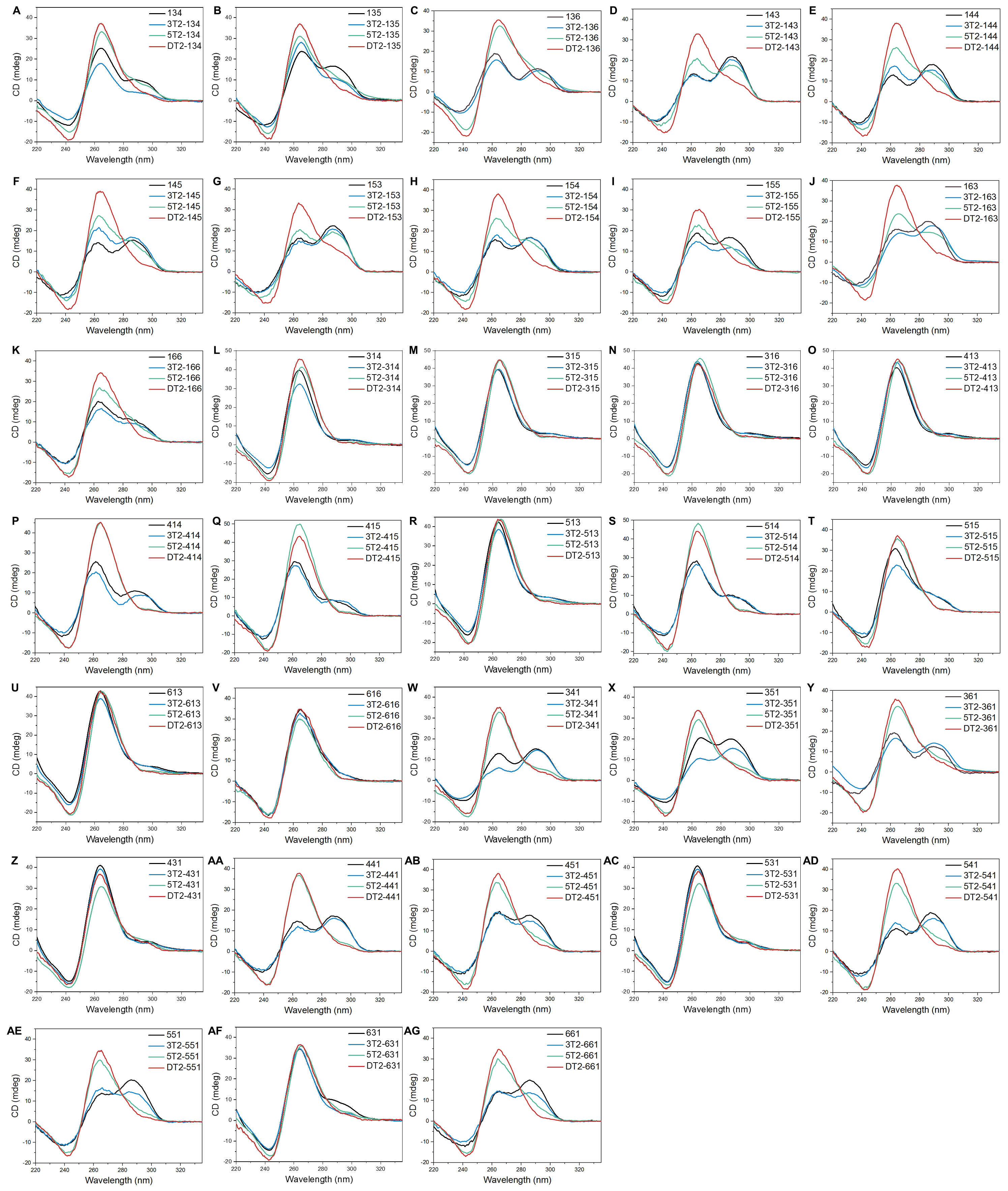


**Figure S12.** CD spectra of sequences (**A**) 134, (**B**) 135, (**C**) 136, (**D**) 143, (**E**) 144, (**F**) 145, (**G**) 153, (**H**) 154, (**I**) 155, (**J**) 163, (**K**) 166, (**L**) 314, (**M**) 315, (**N**) 316, (**O**) 413, (**P**) 414, (**Q**) 415, (**R**) 513, (**S**) 514, (**T**) 515, (**U**) 613, (**V**) 616, (**W**) 341, (**X**) 351, (**Y**) 361, (**Z**) 431, (**AA**) 441, (**AB**) 451, (**AC**) 531, (**AD**) 541, (**AE**) 551, (**AF**) 631, (**AG**) 661, without and with 3ʹ-terminal (3'T2), 5ʹ-terminal (5'T2) or both terminal 2dTs (DT2). CD experiments were carried out at 20 ^o^C by using 5 μM strand concentrations in 100 mM KCl.


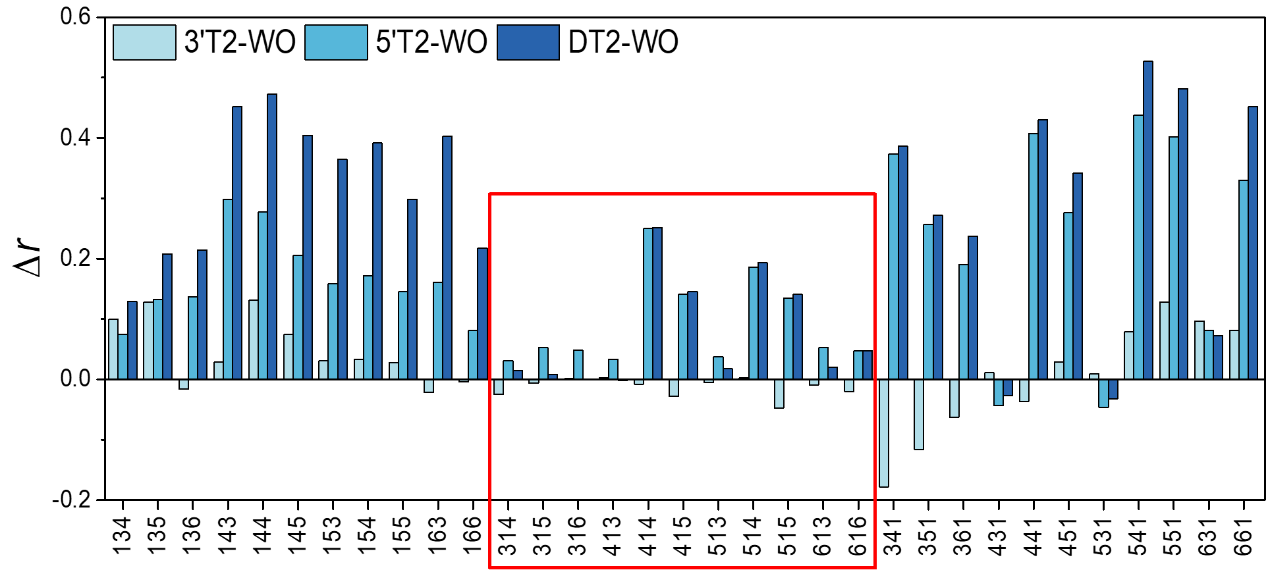


**Figure S13**. The *r* values difference (Δ*r*) of sequences without (WO) and with 3ʹ-terminal (3'T2), 5ʹ-terminal (5'T2) or both terminal (DT2) 2dTs. Experiments were performed in 100 mM KCl.


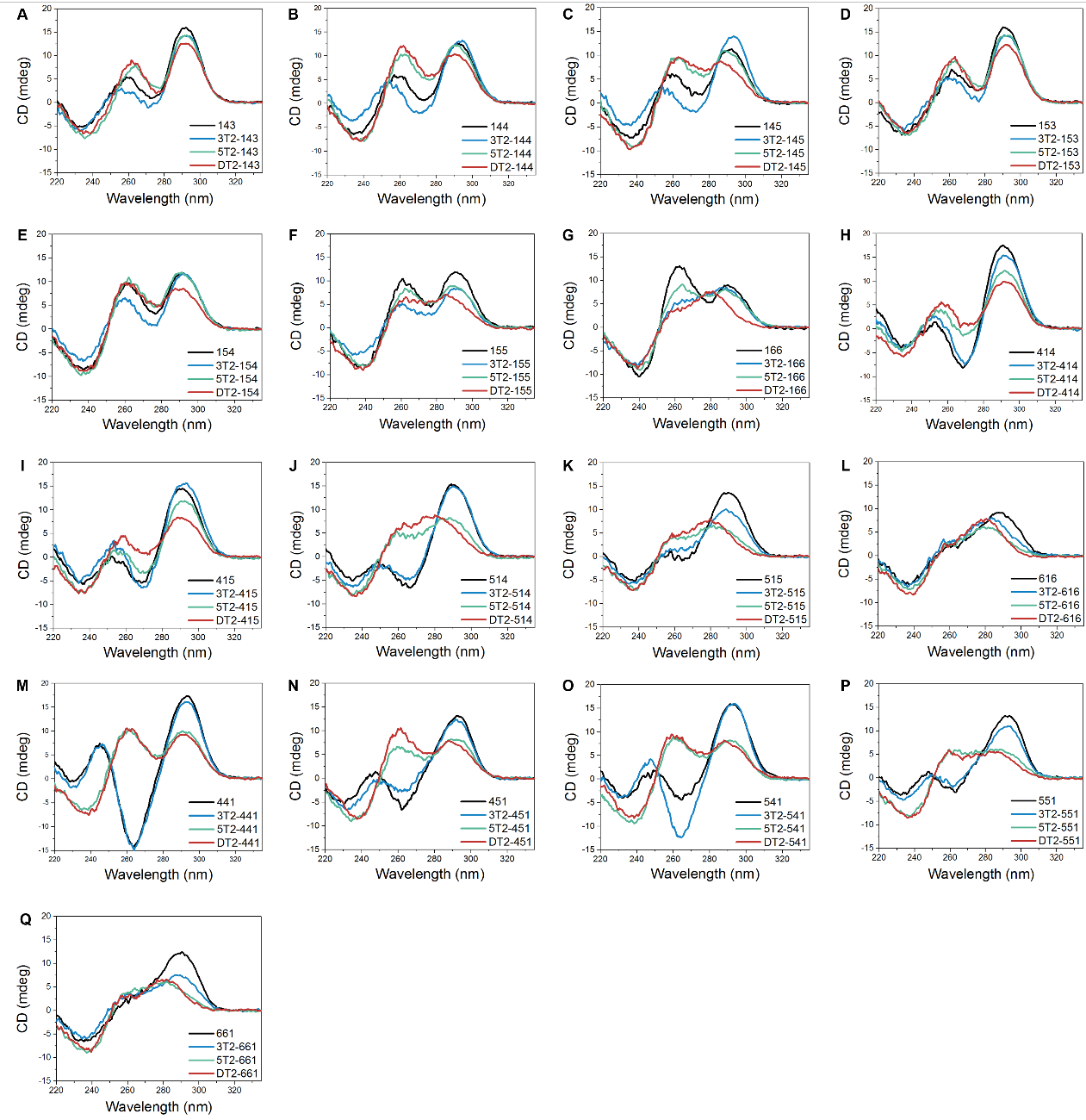


**Figure S14.** CD spectra of sequences (**A**) 143, (**B**) 144, (**C**) 145, (**D**) 153, (**E**) 154, (**F**) 155, (**G**) 166, (**H**) 414, (**I**) 415, (**J**) 514, (**K**) 515, (**L**) 616, (**M**) 441, (**N**) 451, (**O**) 541, (**P**) 551, (**Q**) 661 without and with 3ʹ-terminal (3'T2), 5ʹ-terminal (5'T2) or both terminal 2dTs (DT2). CD experiments were carried out at 20 ^o^C by using 5 μM strand concentrations in 100 mM NaCl.


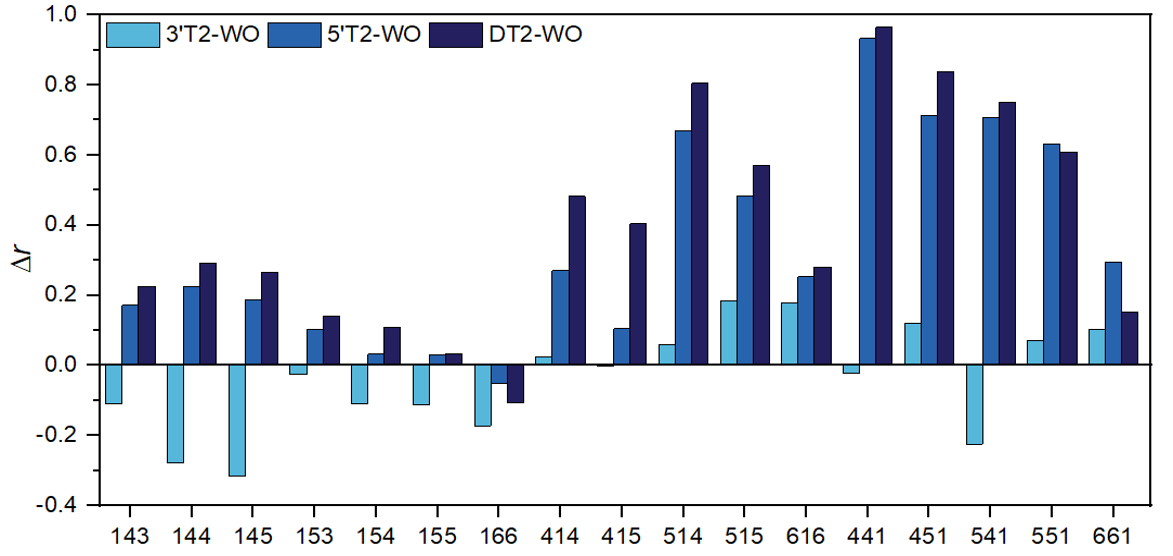


**Figure S15**. The *r* values difference (Δ*r*) of sequences without (WO) and with 3ʹ-terminal (3'T2), 5ʹ-terminal (5'T2) or both terminal (DT2) 2dTs. Experiments were performed in 100 mM NaCl.


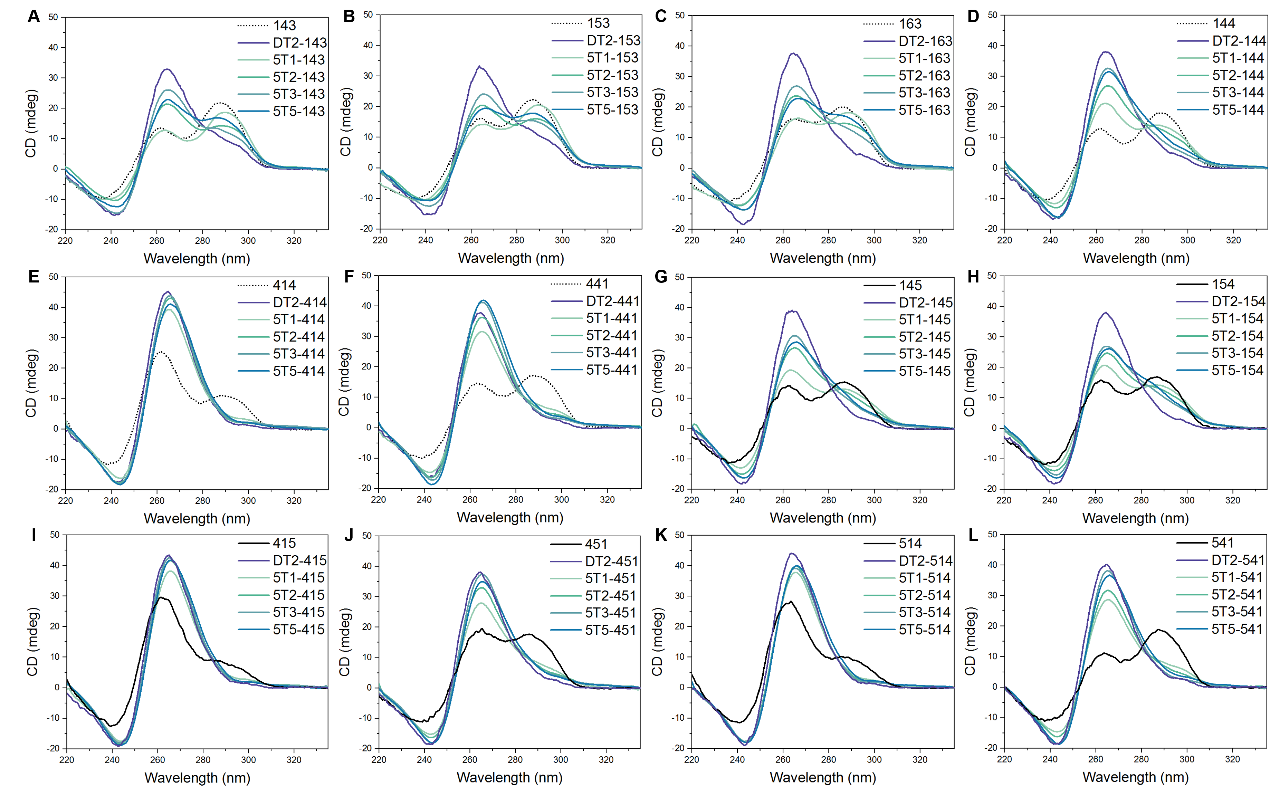


**Figure S16.** CD spectra of sequence (**A**) 143, (**B**) 153, (**C**) 163, (**D**) 144, (**E**) 414, (**F**) 441, (**G**) 145, (**H**) 154, (**I**) 415, (**J**) 451, (**K**) 514, (**L**) 541 with different length of dT (from one thymine to 5 thymines) at 5ʹ-terminal, compared with sequences without and with both terminal 2dTs. CD experiments were carried out at 20 ^o^C by using 5 μM strand concentrations in 100 mM KCl.


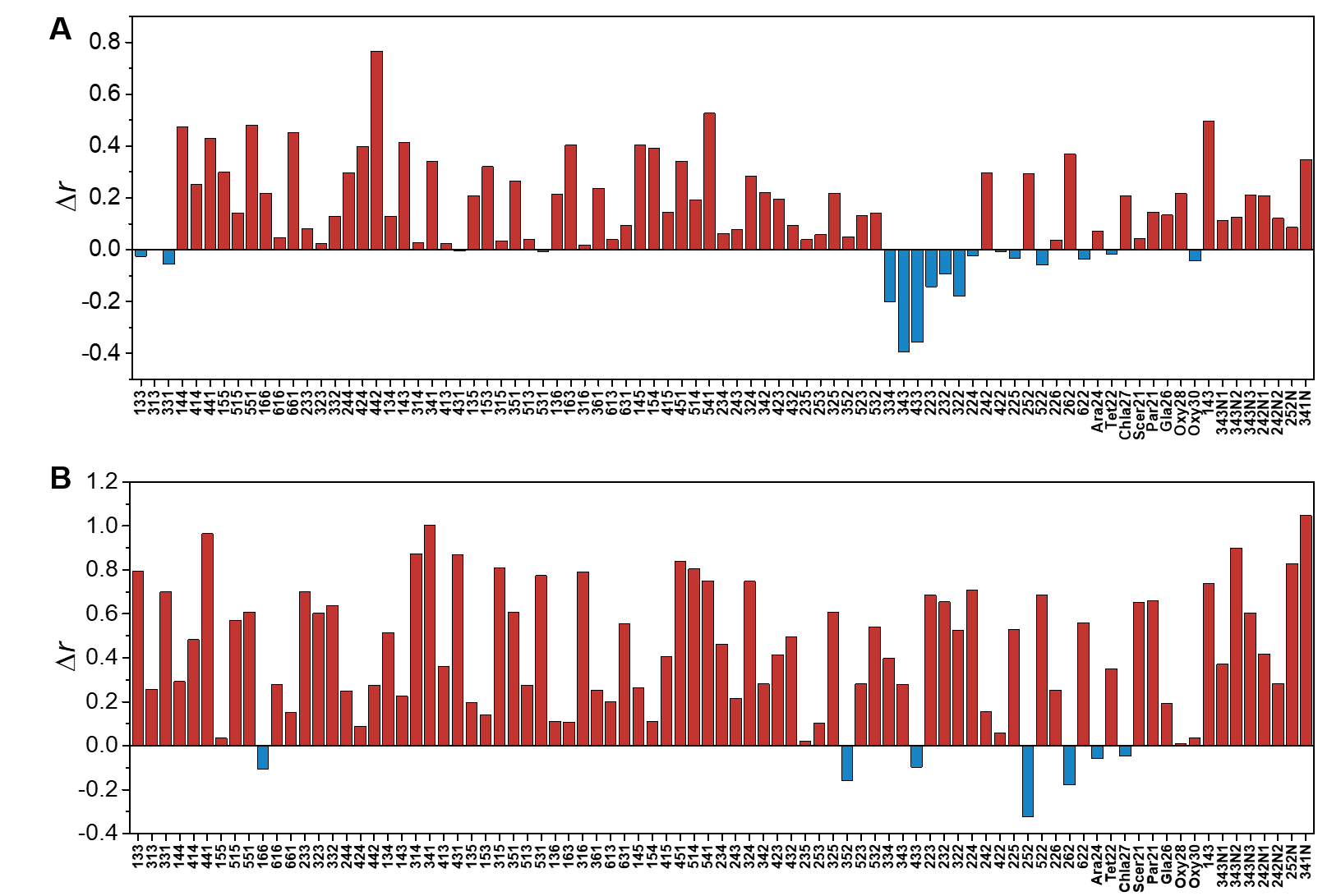


**Figure S17.** The *r* values difference (Δ*r* = *r*_DT2_ - *r*_WO_) for artificial design and natural sequences at 100 mM (**A**) KCl or (**B**) NaCl.


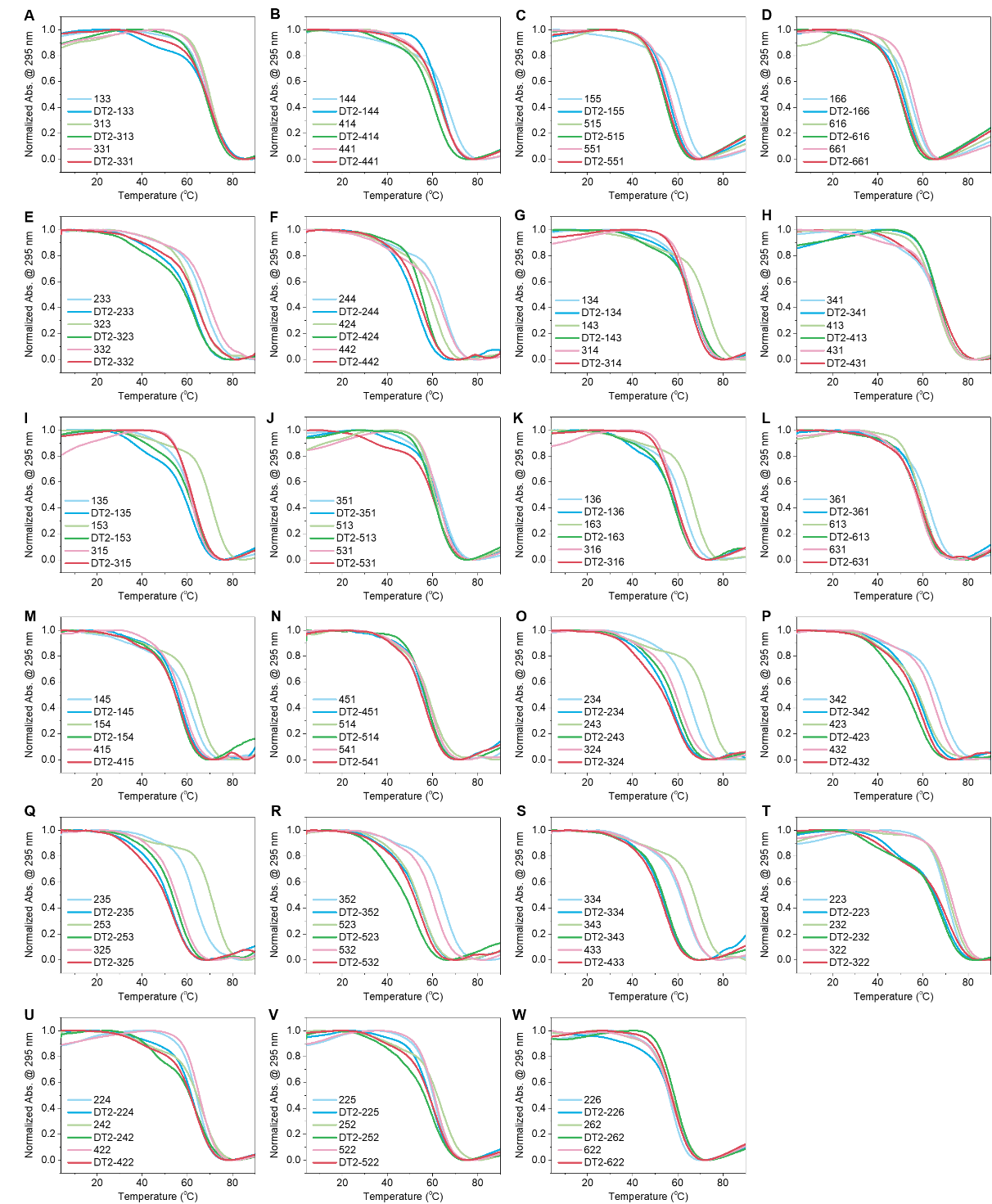


**Figure S18**. UV-melting curves of (**A**) ***133*** group, (**B**) ***144*** group, (**C**) ***155*** group, (**D**) ***166*** group, (**E**) ***233*** group, (**F**) ***244*** group, (**G**, **H**) ***134*** group, (**I**, **J**) ***135*** group, (**K**, **L**) ***136*** group, (**M**, **N**) ***145*** group, (**O**, **P**) ***234*** group, (**Q**, **R**) ***235*** group, (**S**) ***334*** group, (**T**) ***223*** group, (**U**) ***224*** group, (**V**) ***225*** group, and (**W**) ***226*** group without and with both terminal 2dTs. Experiments were carried out in 100 mM KCl. Notes: UV absorbance and melting measurements were performed on SAFAS (Monaco) or Cary100 (Agilent) spectrophotometers by recording the absorbance at 295 nm with 0.5 °C/min from 4.0 to 90.0 °C.


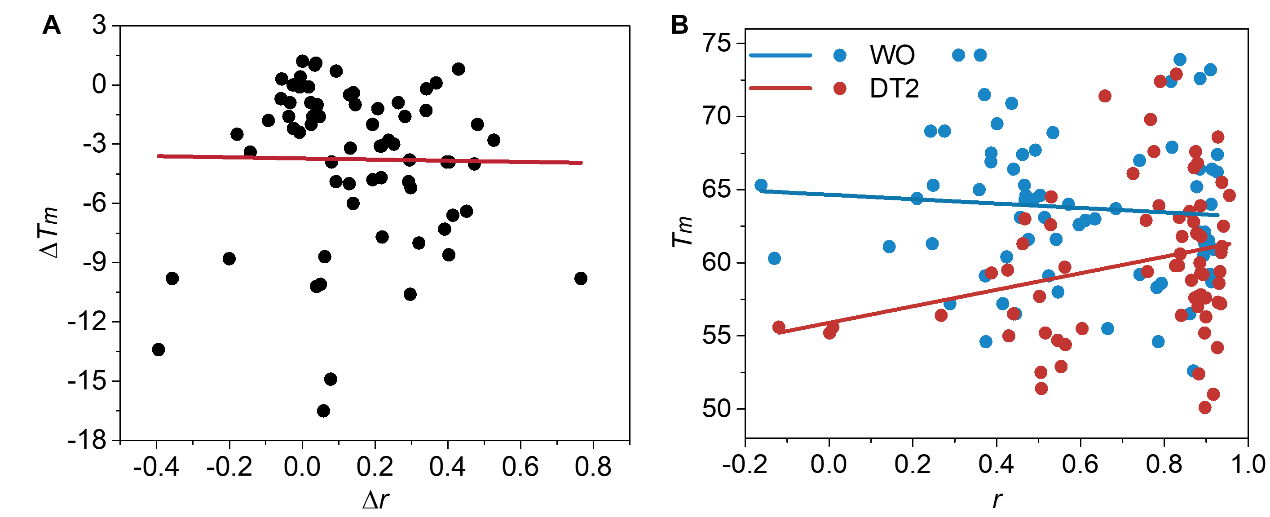


**Figure S19**. (**A**) The relationship between Δ*r* values and Δ*T_m_* for the sequences with (DT2) and without (WO) flanking nucleotides. Δ*T_m_* = -3.72 - 0.29Δ*r* (R^2^ = -0.01). (**B**) The relationship between *r* values and *T_m_*. *T_m_* *_(WO)_* = 64.7 - 1.5*r* (R^2^ = -0.009); *T_m_* *_(DT2)_* = 55.9 + 5.65*r* (R^2^ = 0.06). Experiments were performed in 100 mM KCl.

**
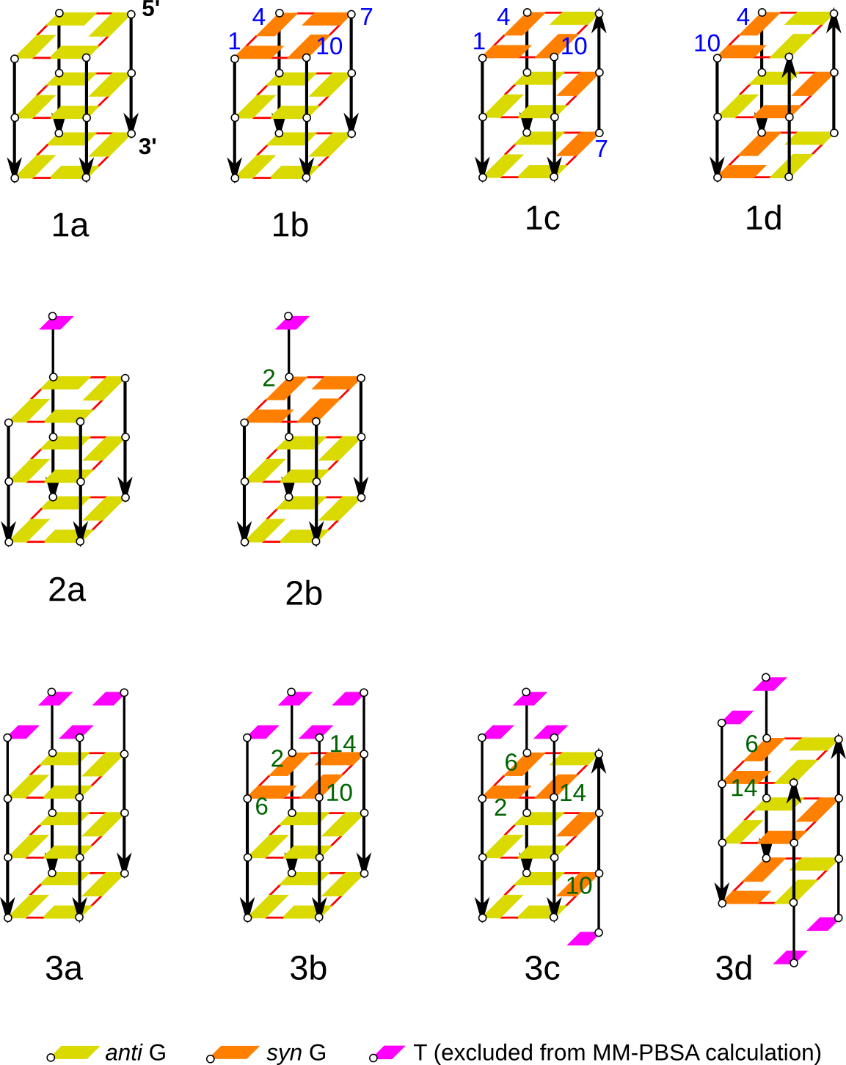
**

**Figure S20**. Models used for explicit solvent MD simulations followed by post-processing MM/PBSA free-energy calculations. The free-energy calculations are done for the G-stems in such a way (see the Supporting Information text) that each evaluated structure contains the same number of atoms, which allows to compare free energies of all models. The main goal of the computations was to estimate the free-energy effect of elimination of the 5ʹ-OH…N3 H-bond (specific for the *syn* conformation) due to addition of the covalent linkage of the 5’-end flanks. The blue numbers in models **1b**, **1c** and **1d** depict guanine numbering used in the 5ʹ-OH…N3 hydrogen-bond population analysis. The green numbers in models **2b**, **3b**, **3c** and **3d** depict guanine numbering used in the N2-H…phosphate hydrogen-bond population analysis.


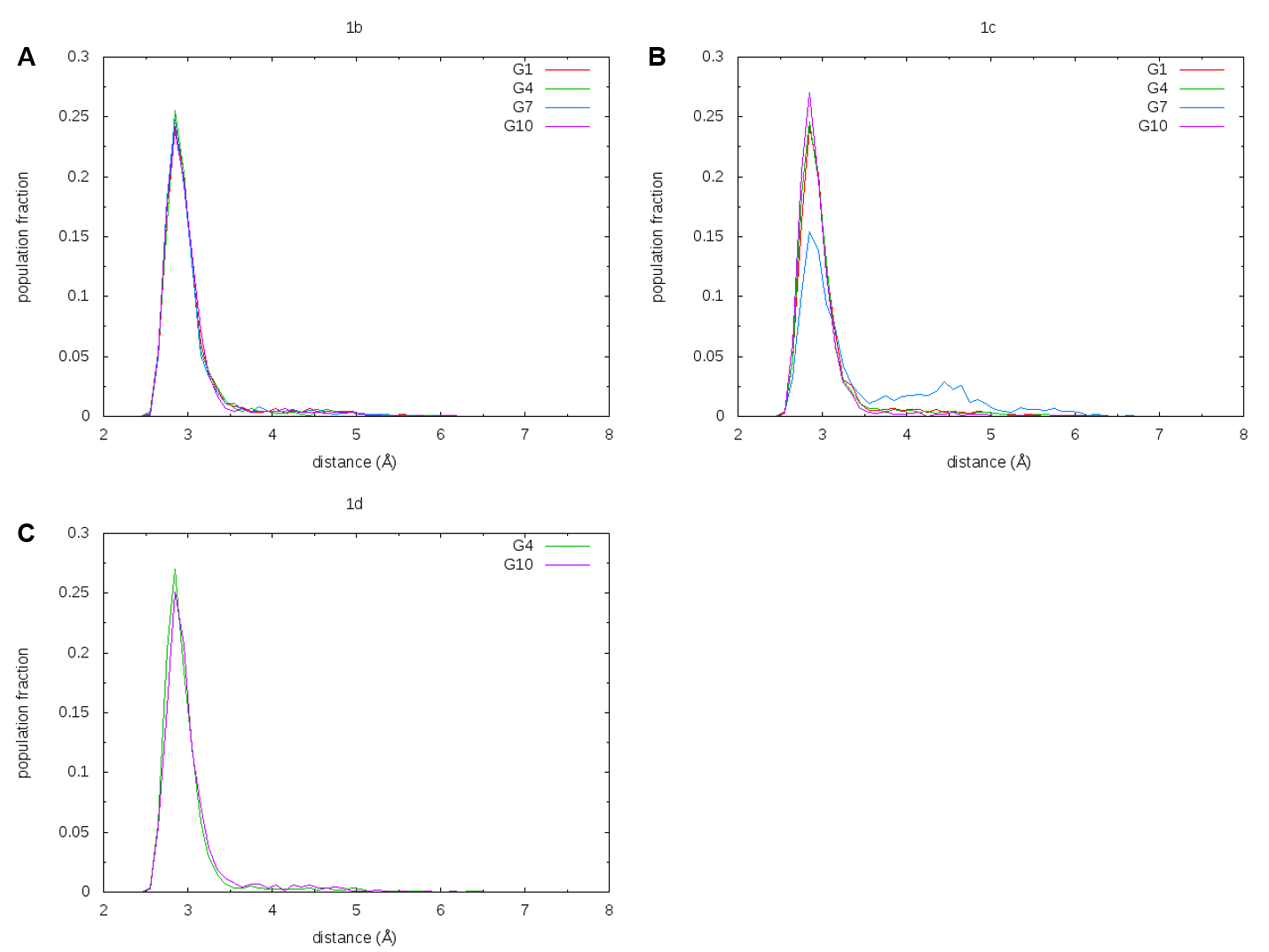


**Figure S21**. Distribution of the O – N distance corresponding to the terminal 5ʹ-OH···N3 hydrogen-bond in simulations of the models (**A**) **1b**, (**B**) **1c** and (**C**) **1d** (see Figure S20 and Table S10). The bin size is 0.1 Å. The peak centred around ~2.8 Å corresponds to the H-bond. Note that additional inclusion of the angle H-bond criterion (135 degree for the O-H···N angle) would have minimal (essentially invisible) effect on the shape and position of the peak, *i.e.,* the distance criterion is fairly sufficient to identify the H-bond for visualisation purposes.


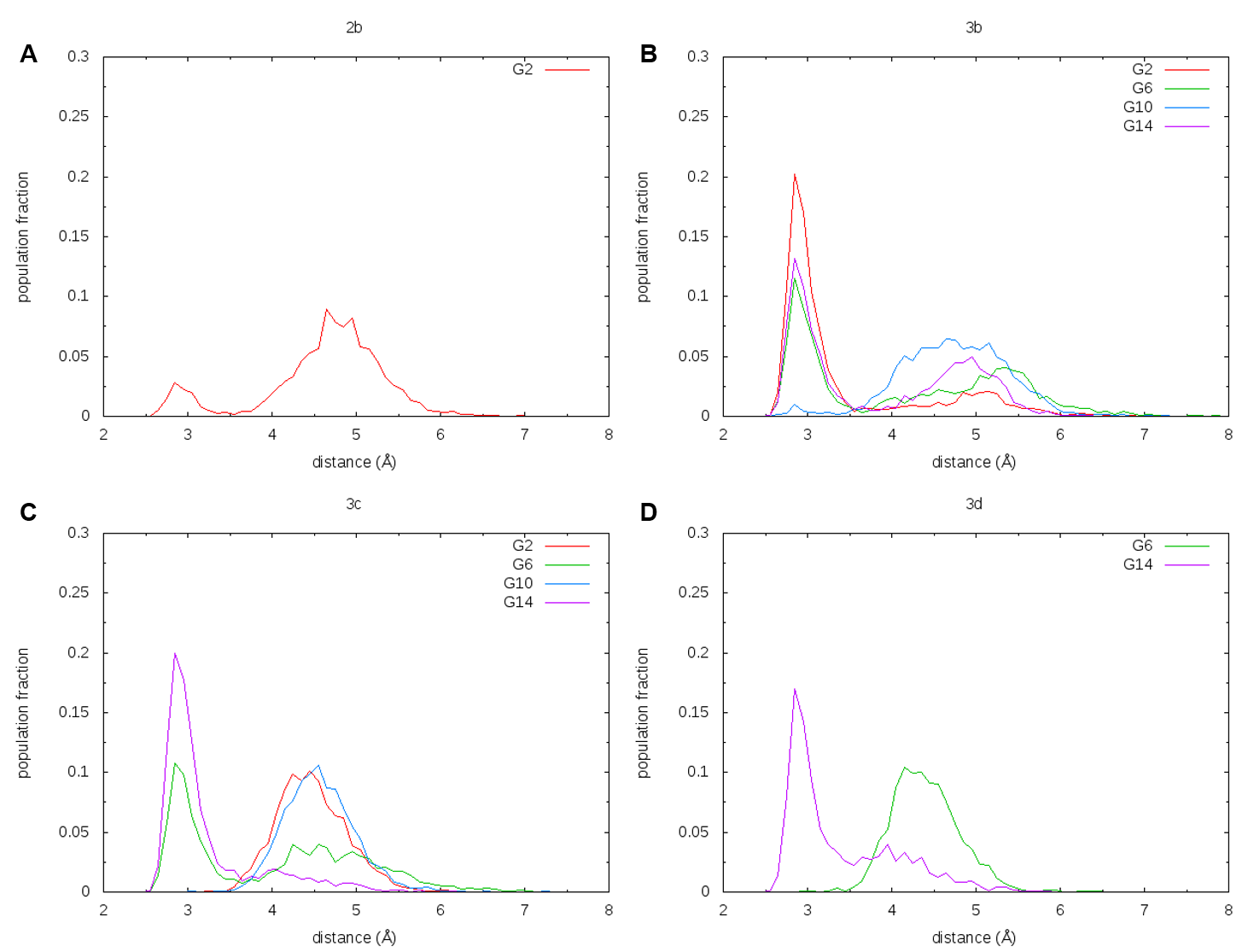


**Figure S22**. Distribution of the N2 – phosphate (OP1/OP2/O5ʹ/O3ʹ, whichever is closest to N2) distance corresponding to the internal base(N2)···phosphate interaction in simulations of the models (**A**) **2b**, (**B**) **3b,** (**C**) **3c** and (**D**) **3d** (see Figure S20 and Table S11). The bin size is 0.1 Å. The peak centred around ~2.8 Å corresponds to the H-bond. Note that additional inclusion of the angle H-bond criterion (135 degree for the N-H···OP1/OP2/O5ʹ/O3ʹ angle) would have minimal (essentially invisible) effect on the shape and position of the peak*, i.e.,* the distance criterion is fairly sufficient to identify the H-bond for visualisation purposes.


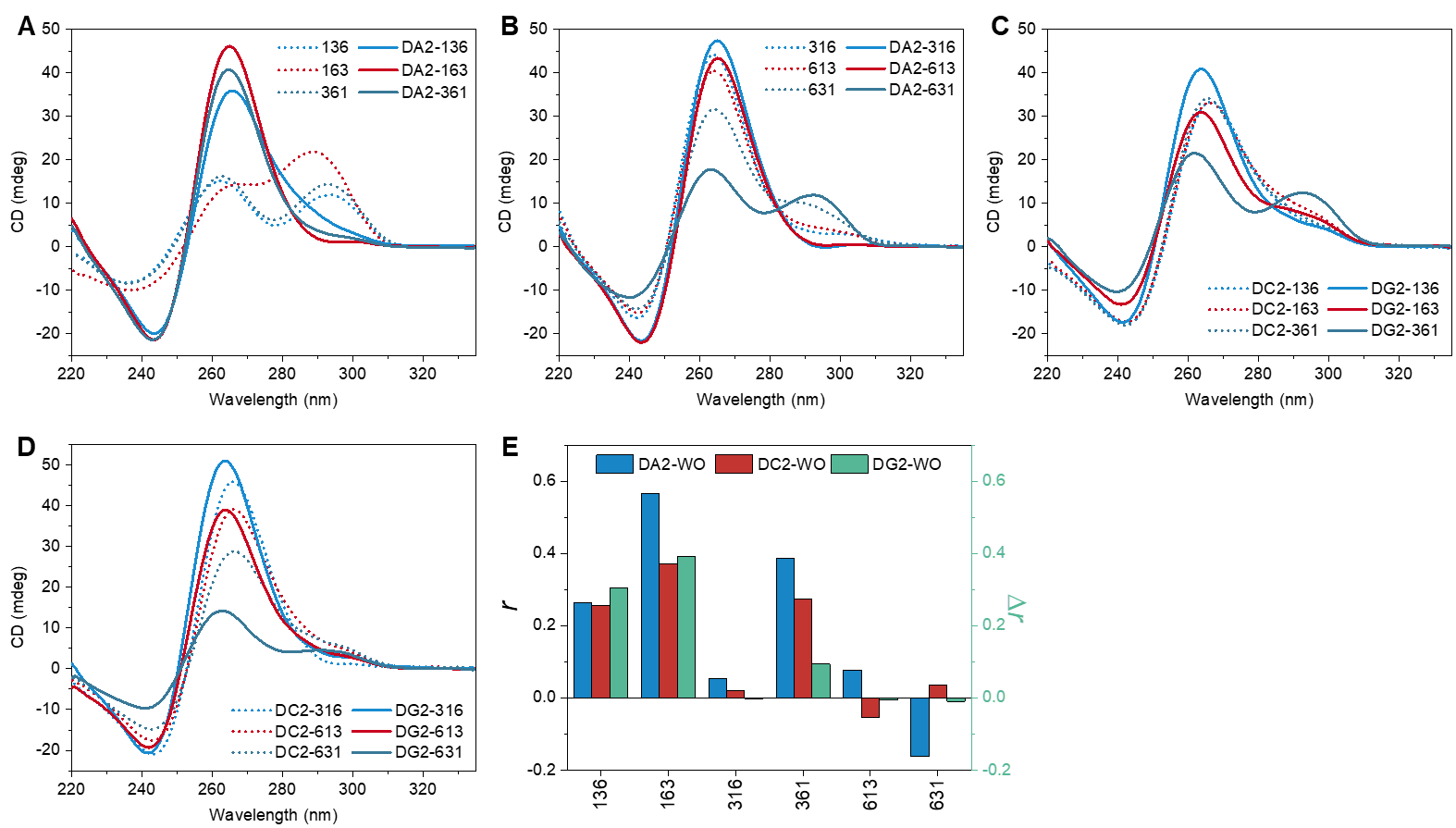


**Figure S23**. CD spectra of sequences with (**A**, **B**) 2dAs, (**C**) 2dC, (**D**) 2dGs overhang at both terminals, (**E**) their r values and differences of ***136*** group. Experiments were performed in 100 mM KCl. Sequence information shown below.

| DA2-136: AA G_3_ T G_3_ T_3_ G_3_ T_6_ G_3_ AA | DC2-361: CC G_3_ T_3_ G_3_ T_6_ G_3_ T G_3_ CC |
| --- | --- |
| DA2-163: AA G_3_ T G_3_ T_6_ G_3_ T_3_ G_3_ AA | DC2-613: CC G_3_ T_6_ G_3_ T G_3_ T_3_ G_3_ CC |
| DA2-316: AA G_3_ T_3_ G_3_ T G_3_ T_6_ G_3_ AA | DC2-631: CC G_3_ T_6_ G_3_ T_3_ G_3_ T G_3_ CC |
| DA2-361: AA G_3_ T_3_ G_3_ T_6_ G_3_ T G_3_ AA | DG2-136: GG G_3_ T G_3_ T_3_ G_3_ T_6_ G_3_ GG |
| DA2-613: AA G_3_ T_6_ G_3_ T G_3_ T_3_ G_3_ AA | DG2-163: GG G_3_ T G_3_ T_6_ G_3_ T_3_ G_3_ GG |
| DA2-631: AA G_3_ T_6_ G_3_ T_3_ G_3_ T G_3_ AA | DG2-316: GG G_3_ T_3_ G_3_ T G_3_ T_6_ G_3_ GG |
| DC2-136: CC G_3_ T G_3_ T_3_ G_3_ T_6_ G_3_ CC | DG2-361: GG G_3_ T_3_ G_3_ T_6_ G_3_ T G_3_ GG |
| DC2-163: CC G_3_ T G_3_ T_6_ G_3_ T_3_ G_3_ CC | DG2-613: GG G_3_ T_6_ G_3_ T G_3_ T_3_ G_3_ GG |
| DC2-316: CC G_3_ T_3_ G_3_ T G_3_ T_6_ G_3_ CC | DG2-631: GG G_3_ T_6_ G_3_ T_3_ G_3_ T G_3_ GG |

**
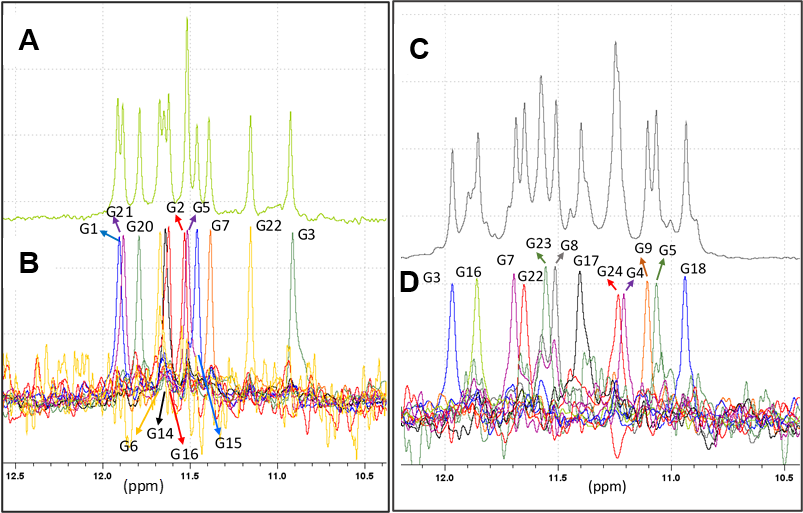
**

**Figure S24**. (**A**, **C**) 1D ^1^H-NMR spectra and (**B**, **D**) 1D {^1^H-^15^N} HSQC NMR obtained from 12 individual samples isotopically labelled at individual guanine positions for (**A**, **B**) 163 and (**C**, **D**) DT2-163. All samples were prepared in 10 mM KPi buffer, acquired at 25°C. The resolution of 12 guanines is indicative of a three-tetrad core G-quadruplex as expected.

**Procedure of NMR Experiments**

^1^H NMR experiments were performed on 400, 600, 700, or 800 MHz Advance III/NEO Bruker Advance spectrometers, equipped with cryoprobes. Oligonucleotides were prepared at concentrations ranging from 100 μM to 1.4 mM in 10 mM KPi buffer (10 mM K_2_HPO_4_/KH_2_PO_4_, pH 6.6, and the K^+^ concentration was adjusted to 100 mM) and subjected to annealing cycles:, consisting of heating at 95 °C and then chilling in an ice-bath at least three times.

5% isotopically enriched samples site-specifically labeled with ^15^N and ^13^C were synthesized in our laboratory (INSERM U1212, Bordeaux) using dGiBu-phosphoramidites (U-^13^C10, 98%; U-^15^N5, 98%; CP 95%) purchased from Cambridge Isotope Laboratories. All the standard phosphoramidites (dABz; dT; dGiBu; dCAc), reagents, and solvents used during the synthesis were purchased from Glen Research and used according to provider instructions. After the synthesis, the oligonucleotides were cleaved from the support and the nucleobases were deprotected by incubation with ammonium hydroxide at 55 °C for 16 hours, and then lyophilized before use.


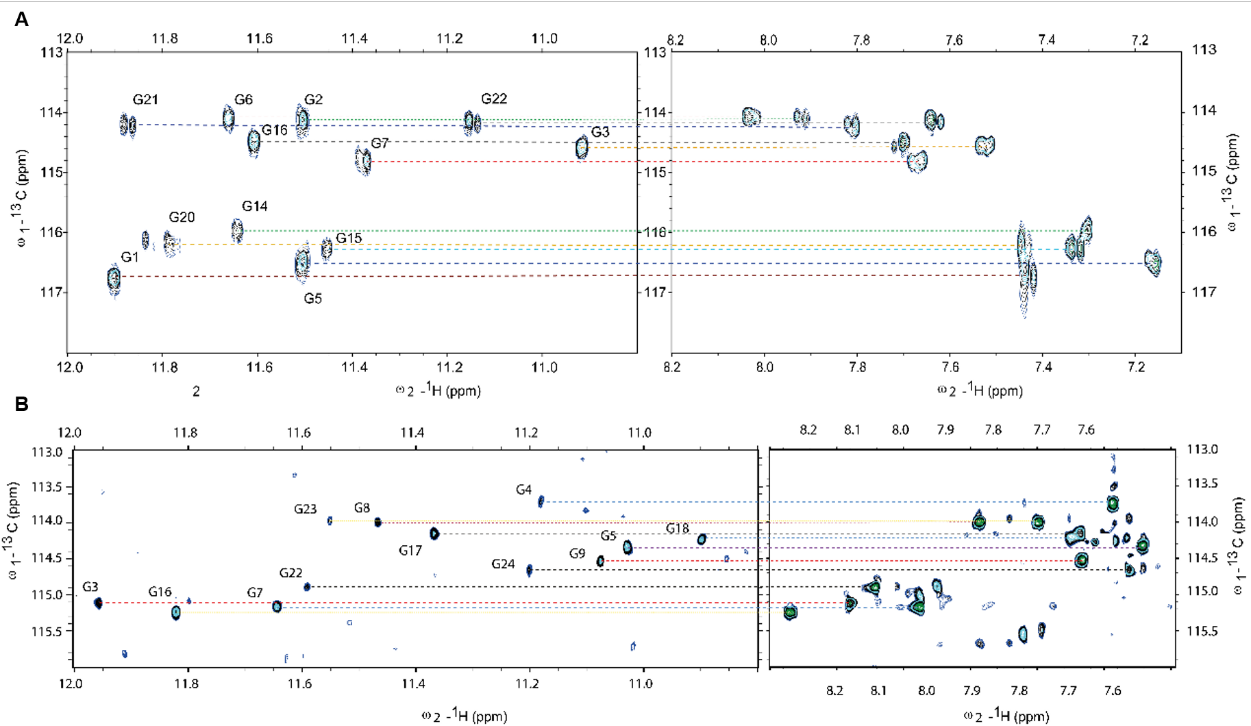


**Figure S25**. 2D ^1^H-^13^C JR-HMBC NMR spectra of (**A**) 163 and (**B**) DT2-163. Each spectrum allows to correlate the H8 and H1 imino guanine protons through C5 (^13^C natural abundance) guanine carbon atoms for 163 and DT2-163 respectively. Both samples were prepared in 10 mM KPi buffer. The above spectra were acquired at 25°C.


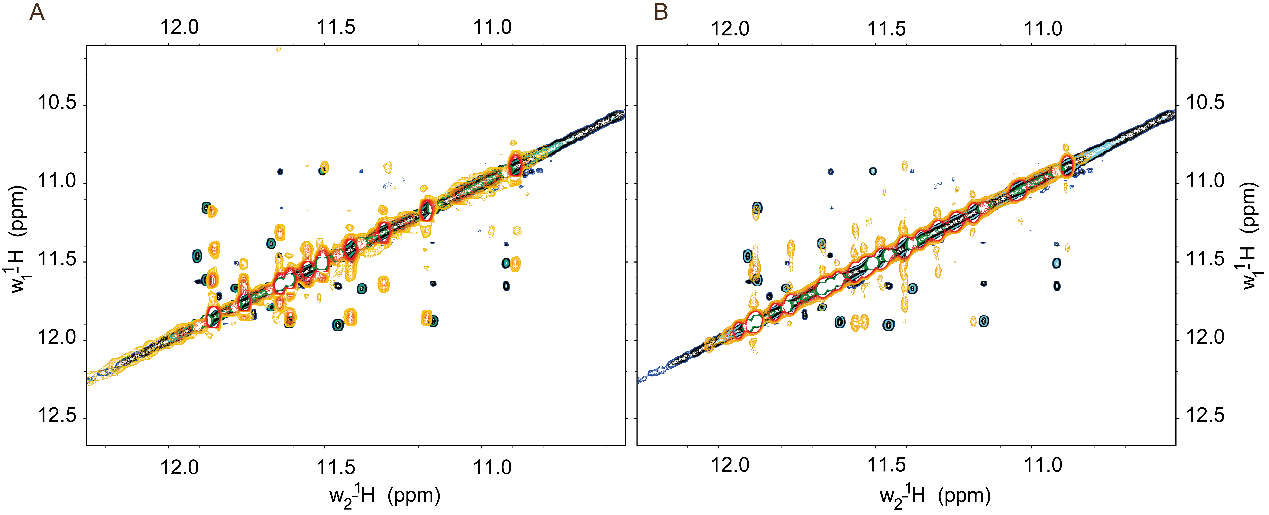


**Figure S26**. 2D ^1^H-^1^H NOESY NMR spectra depicting the imino region of oligo 163 (blue-green), overlay with 3'T2-163 and 5'T2-163 (yellow-orange) in panels **A** and **B,** respectively. (**A**) Small modifications on the imino peak-pattern of 3'T2-163 indicated that both 163 and 3'T2-163 showed antiparallel conformations. (**B**) 5'-end 2dTs made important modifications in the imino pattern, indicative of important conformational change towards all parallel.


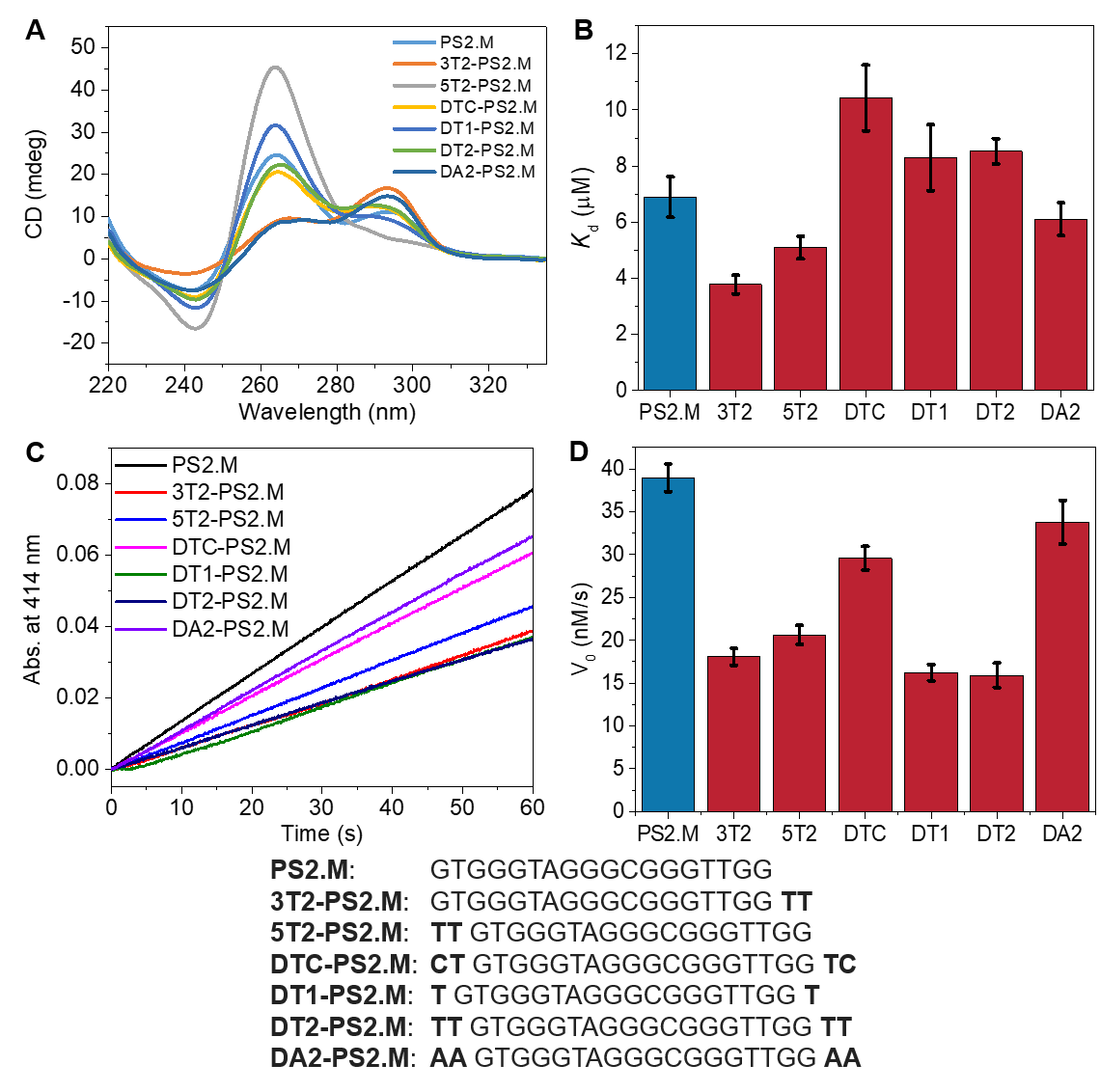


**Figure S27.** The influence of flanking nucleotides on the conformation and function for hemin aptamer, PS2.M. (**A**) The CD spectra for sequences with different flanking nucleotides at the terminal of PS2.M. (**B**) The binding affinity between PS2.M, its analogues and hemin. (**C**, **D**) The catalytic activity for the DNAzyme formed by hemin with PS2.M and its analogues. The DNAzyme activity experiments were performed with 0.4 μM DNA, 0.8 μM hemin, 0.6 mM ABTS and 0.6 mM H_2_O_2_ in 10 mM Tris-HCl buffer (pH 7.0) at 25 ^o^C. Catalytic experiments were performed as described previously (S37).


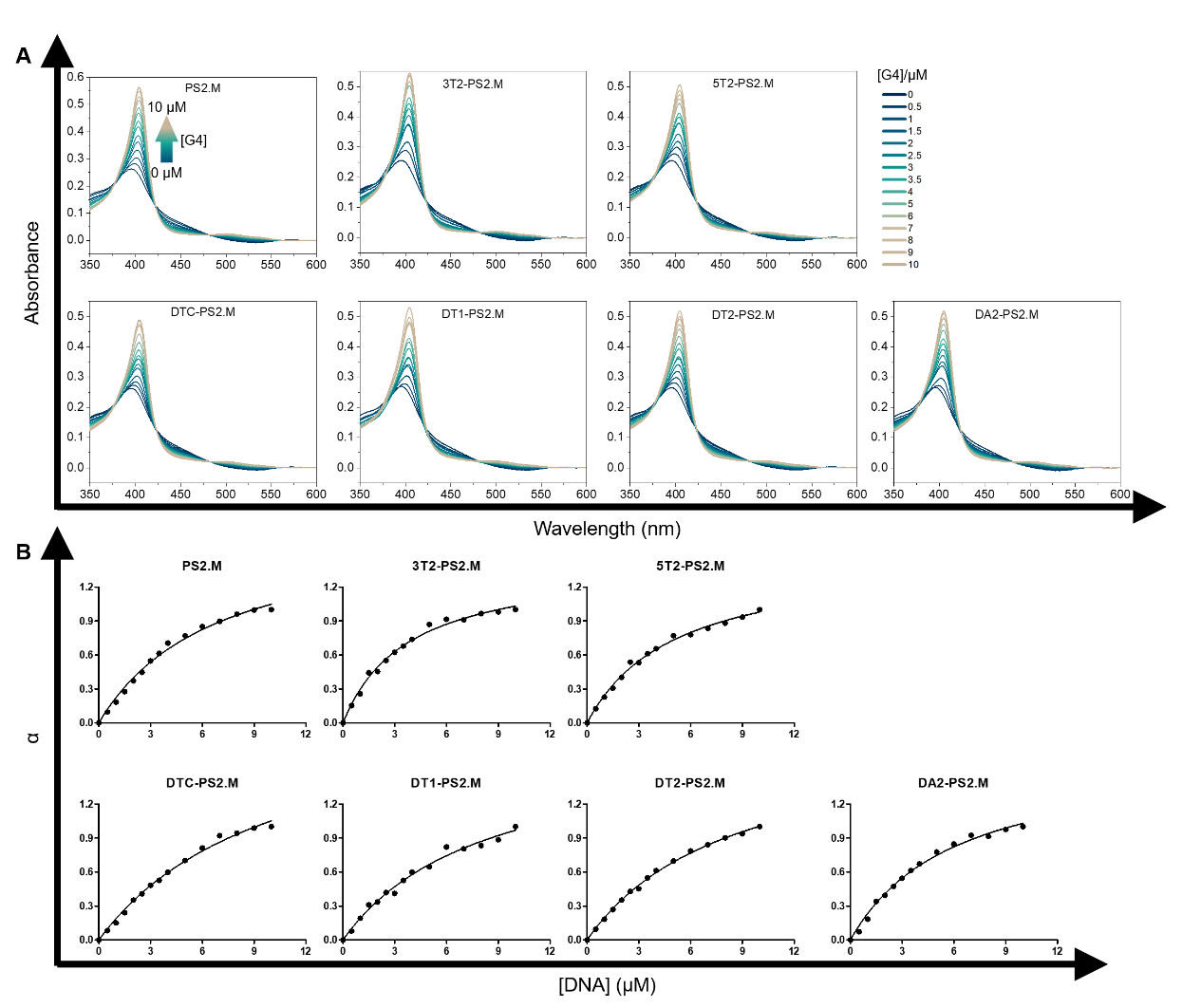


**Figure S28.** Titration for hemin with PS2.M and its analogues. (**A**) UV-Vis spectra of 5 μM hemin with increasing concentration of PS2.M and sequences with different flanking nucleotides, from 0 to 10 μM. (**B**) Plots of the fraction of bound hemin as a function DNA concentration. α is the bound fraction of hemin by PS2.M and its analogues.

To evaluate the affinity between G4s and hemin, spectrophotometric titration of 5 μM hemin with increasing concentration of DNA was performed by incubating G4 (0 to 10 μM) in 10 mM Tris-HCl buffer at 25 ^o^C for 2 h. Spectra were collected from 350 to 600 nm. The saturation curve for the binding of hemin with G4s was plotted by bound fraction (α, Eq. **S1**) of hemin *versus* DNA concentration. *K*_d_ was obtained by fitting of the curve with a one-site binding model (GraphPad Prism 5).

$\alpha=\frac{A_{x}-A_{0}}{A_{\infty}-A_{0}}$ [**S1**]

where *A_x_* is the absorbance at 405 nm for G4s incubated with hemin, *A_∞_* and *A_0_* are the respective values in the presence of saturating G4s and in the absence of G4s, respectively.

The spectrophotometric titration of hemin with G4s was characterized by a red shift of the Soret band from 399 to 405 nm, with a sharp hyperchromism. Then the fraction of bound hemin reached to a plateau with the DNA concentration. Finally, the saturation curves were fit with a one-site binding model for every sequence.


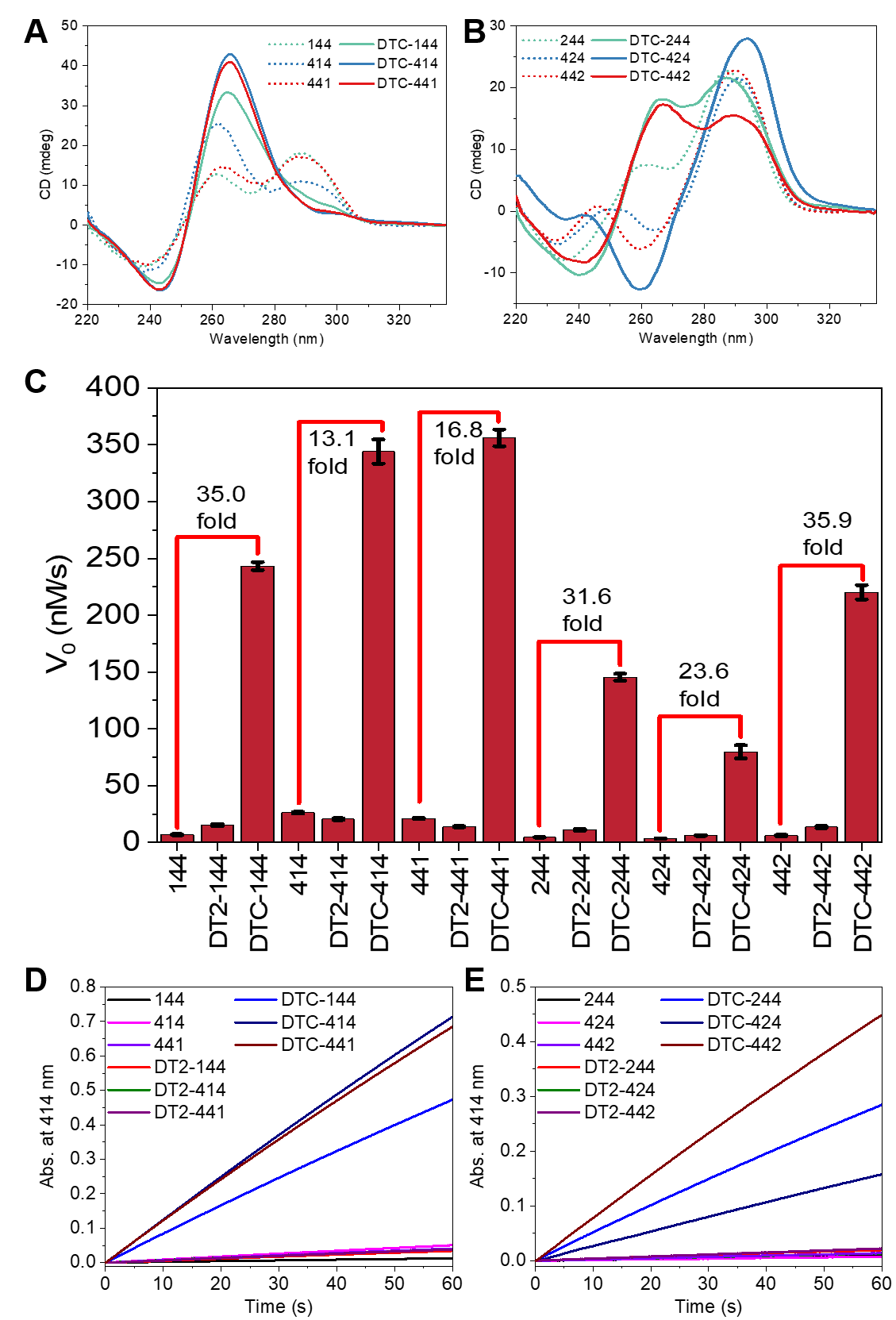


DTC-144: CT GGG T GGG TTTT GGG TTTT GGG TC

DTC-414: CT GGG TTTT GGG T GGG TTTT GGG TC

DTC-441: CT GGG TTTT GGG TTTT GGG T GGG TC

DTC-244: CT GGG TT GGG TTTT GGG TTTT GGG TC

DTC-424: CT GGG TTTT GGG TT GGG TTTT GGG TC

DTC-442: CT GGG TTTT GGG TTTT GGG TT GGG TC

**Figure S29.** The influence of flanking nucleotides on the conformation and the DNAzyme activity formed by hemin with G4 sequences (***144*** and ***244*** groups). (**A**, **B**) The CD spectra for sequences with 2dTCs at both terminals of (**A**) ***144*** and (**B**) ***244*** groups, with 100 mM KCl. (**C**) The catalytic activity and (**D**, **E**) reaction dynamics for (**D**) ***144*** and (**E**) ***244*** groups. The DNAzyme activity experiments were performed with 0.4 μM DNA, 0.8 μM hemin, 0.6 mM ABTS and 0.6 mM H_2_O_2_ in 10 mM Tris-HCl buffer (pH 7.0) at 25 ^o^C.


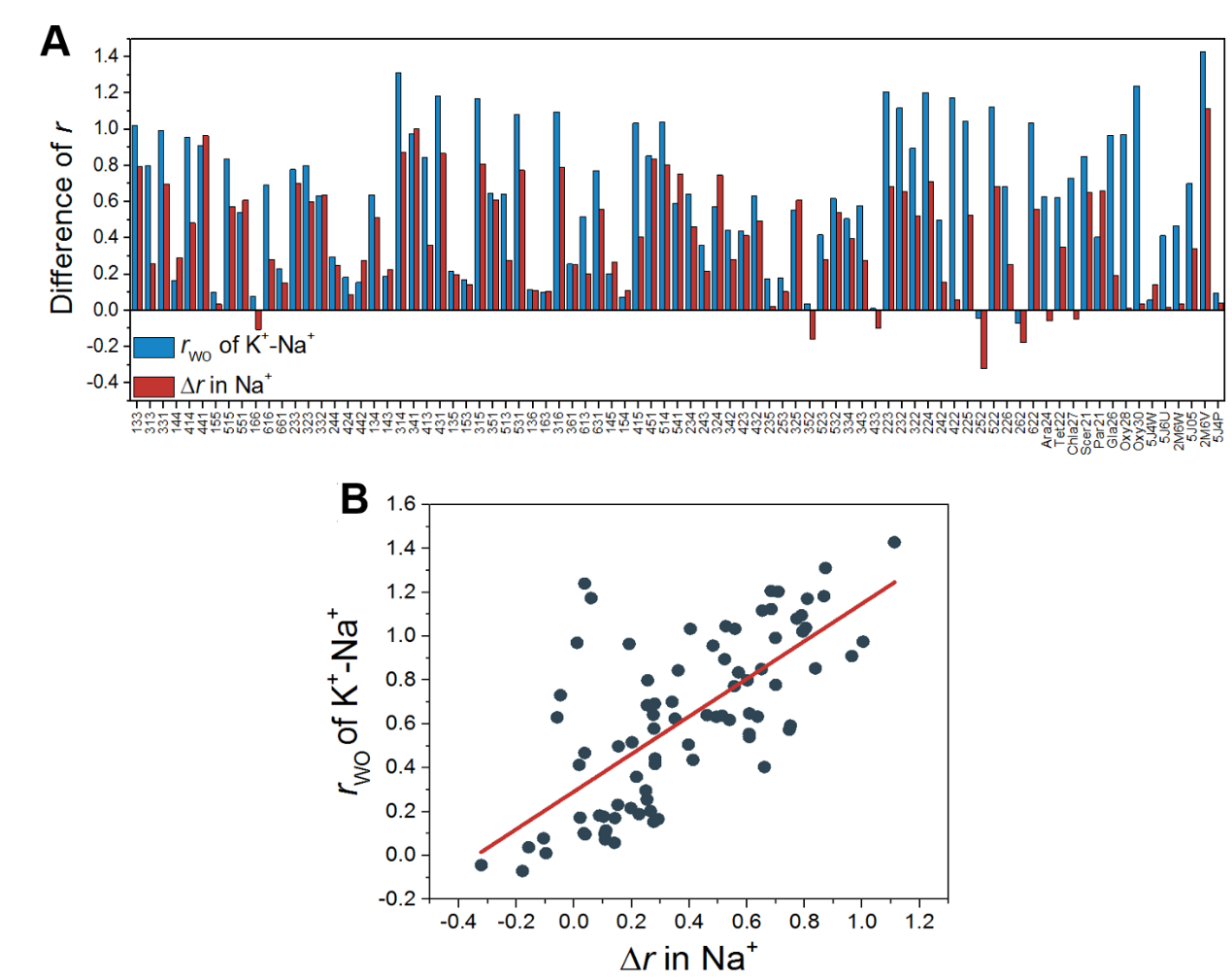


**Figure S30**. (**A**) The difference of *r* values with and without flanking 2dTs at both terminals in Na^+^-buffer (Δ*r* in Na^+^); and the difference of *r* values in K^+^- and Na^+^-buffer for sequences without flanking nucleotides (*r*_WO_ in K^+^- *r*_WO_ in Na^+^) and (**B**) their relationship, *r*_WO_ _(K_^+^_-Na_^+^_)_ = 0.29 + 0.86 Δ*r* (R^2^ = 0.49).


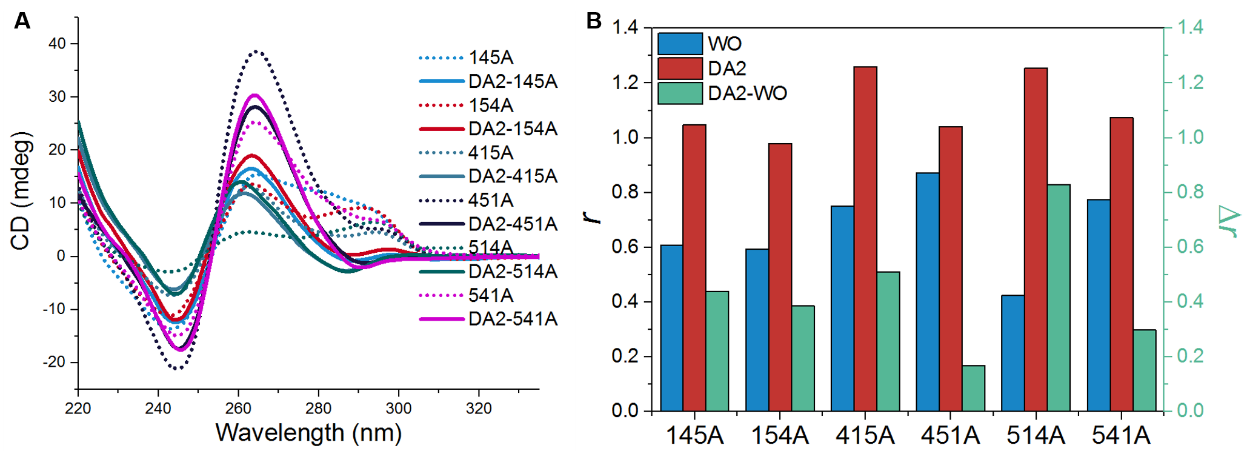


**Figure S31**. (**A**) CD spectra for ***145A*** group with all-adenine flanking and loop sequences. (**B**) The *r* values of sequences without (WO) and with (DA2) both terminal 2dAs, and their difference (Δ*r*). Experiments were carried out at 20 ^o^C by using 5 μM strand concentrations in 100 mM KCl.

| Name | Sequence (5'→3') |
| --- | --- |
| 145A | G_3_ A G_3_ A_4_ G_3_ A_5_ G_3_ |
| 154A | G_3_ A G_3_ A_5_ G_3_ A_4_ G_3_ |
| 415A | G_3_ A_4_ G_3_ A G_3_ A_5_ G_3_ |
| 451A | G_3_ A_4_ G_3_ A_5_ G_3_ A G_3_ |
| 514A | G_3_ A_5_ G_3_ A G_3_ A_4_ G_3_ |
| 541A | G_3_ A_5_ G_3_ A_4_ G_3_ A G_3_ |


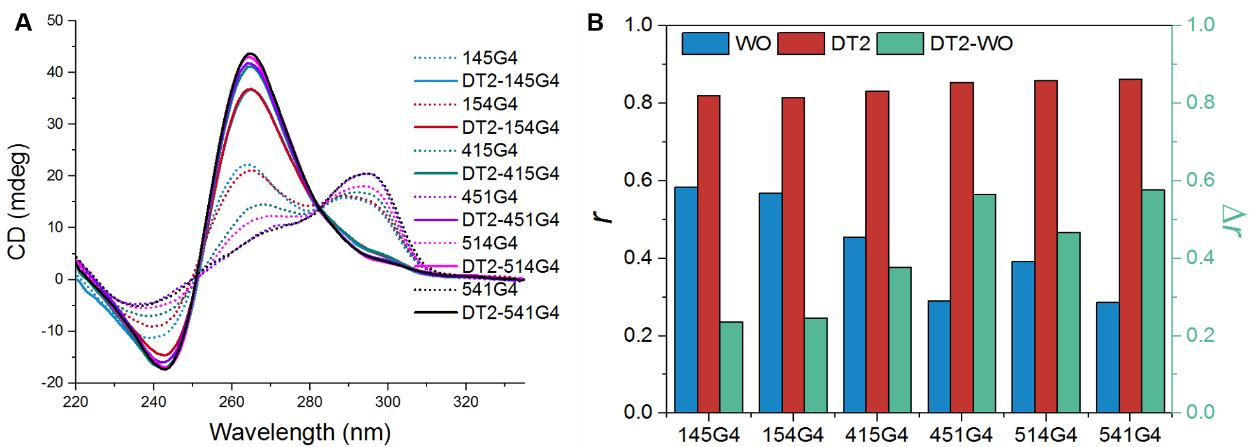


**Figure S32**. (**A**) CD spectra for ***145G4*** group with four G-quartets. (**B**) The *r* values of sequences without (WO) and with (DT2) both terminal 2dTs, and their difference (Δ*r*). Experiments were carried out at 20 ^o^C by using 5 μM strand concentrations in 100 mM KCl.

| Name | Sequence (5'→3') |
| --- | --- |
| 145G4 | G_4_ T G_3_ T_4_ G_3_ T_5_ G_4_ |
| 154G4 | G_4_ T G_4_ T_5_ G_4_ T_4_ G_4_ |
| 415G4 | G_4_ T_4_ G_4_ T G_4_ T_5_ G_4_ |
| 451G4 | G_4_ T_4_ G_4_ T_5_ G_4_ T G_4_ |
| 514G4 | G_4_ T_5_ G_4_ T G_4_ T_4_ G_4_ |
| 541G4 | G_4_ T_5_ G_4_ T_4_ G_4_ T G_4_ |


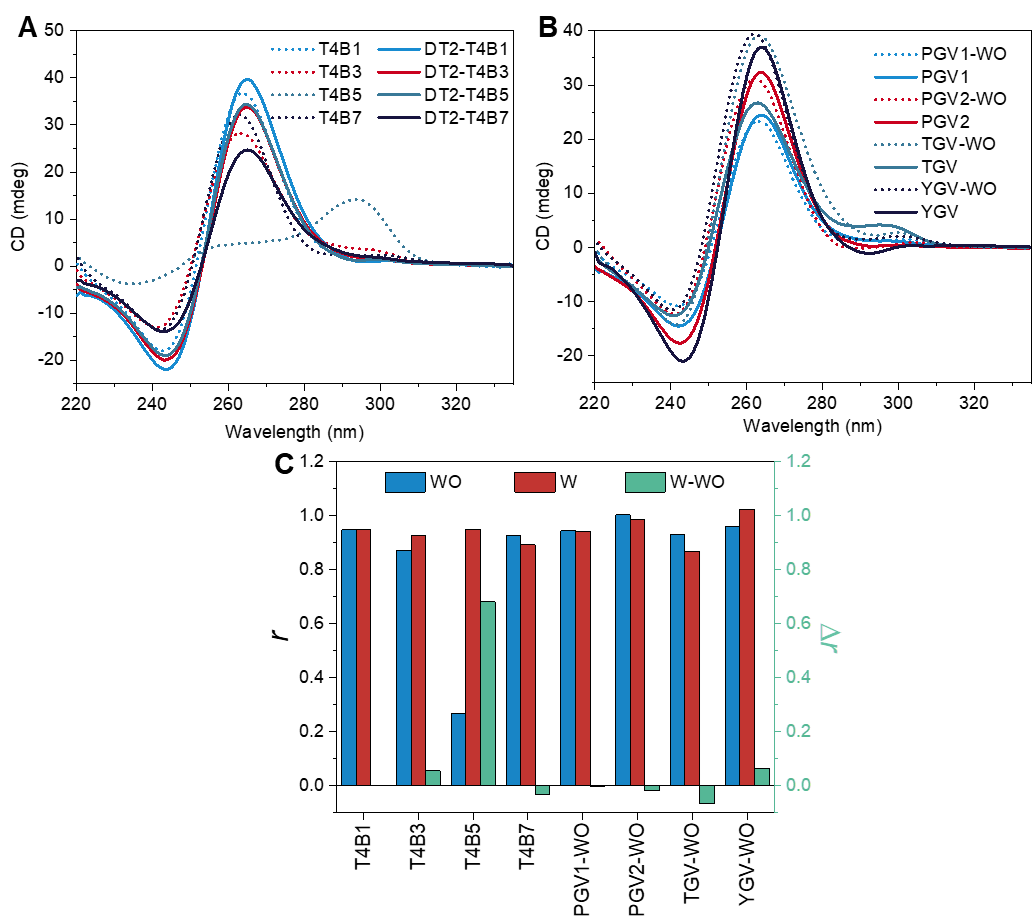


| Name | Sequence (5'→3') | Name | Sequence (5'→3') |
| --- | --- | --- | --- |
| TGV | A G_3_ T G_3_ CT G_3_ A G_2_ T | T4B1 | GT_4_G_2_ T G_3_ T G_3_ T G_3_ |
| TGV-WO | G_3_ T G_3_ CT G_3_ A G_2_ | DT2-T4B1 | TT GT_4_G_2_ T G_3_ T G_3_ T G_3_ TT |
| PGV1 | TT GTG T G_3_ T G_3_ T G_3_ T | T4B3 | G_3_ T GT_4_G_2_ T G_3_ T G_3_ |
| PGV1-WO | GTG T G_3_ T G_3_ T G_3_ | DT2-T4B3 | TT G_3_ T GT_4_G_2_ T G_3_ T G_3_ TT |
| PGV2 | TT G_2_ T G_3_ T G_3_ T G_3_ T | T4B5 | G_3_ T G_3_ T GT_4_G_2_ T G_3_ |
| PGV2-WO | G_2_ T G_3_ T G_3_ T G_3_ | DT2-T4B5 | TT G_3_ T G_3_ T GT_4_G_2_ T G_3_ TT |
| YGV | AA G_3_ A G_3_ C G_2_ C G_3_ ACA | T4B7 | G_3_ T G_3_ T G_3_ T GT_4_G_2_ |
| YGV-WO | G_3_ A G_3_ C G_2_ C G_3_ | DT2-T4B7 | TT G_3_ T G_3_ T G_3_ T GT_4_G_2_ TT |

**Figure S33**. CD spectra of G-rich sequences contain (**A**) G-vacancy, (**B**) bulge with 2dTs added at both terminals, (**C**) their r values and differences with and without flanking nucleotides. Experiments were performed in 100 mM KCl.

**References**

1. Clark, G.R., Pytel, P.D. and Squire, C.J. (2012) The high-resolution crystal structure of a parallel intermolecular DNA G-4 quadruplex/drug complex employing syn glycosyl linkages. *Nucleic Acids Res.*, **40**, 5731-5738.
2. Wang, Y. and Patel, D.J. (1993) Solution structure of the human telomeric repeat d[AG3(T2AG3)3] G-tetraplex. *Structure*, **1**, 263-282.
3. Luu, K.N., Phan, A.T., Kuryavyi, V., Lacroix, L. and Patel, D.J. (2006) Structure of the human telomere in K^+^ solution: an intramolecular (3+1) G-quadruplex scaffold. *J. Am. Chem. Soc.*, **128**, 9963-9970.
4. Case, D., Ben-Shalom, I., Brozell, S., Cerutti, D., Cheatham III, T., Cruzeiro, V., Darden, T., Duke, R., Ghoreishi, D., Gilson, M.K. *et al.*, (2018) San Francisco. AMBER 2018.
5. Berendsen, H.J.C., Grigera, J.R. and Straatsma, T.P. (1987) The Missing Term in Effective Pair Potentials. *J. Phys. Chem.*, **91**, 6269-6271.
6. Joung, I.S. and Cheatham, T.E., 3rd. (2008) Determination of alkali and halide monovalent ion parameters for use in explicitly solvated biomolecular simulations. *J. Phys. Chem. B*, **112**, 9020-9041.
7. Zgarbova, M., Sponer, J., Otyepka, M., Cheatham, T.E., 3rd, Galindo-Murillo, R. and Jurecka, P. (2015) Refinement of the Sugar-Phosphate Backbone Torsion Beta for AMBER Force Fields Improves the Description of Z- and B-DNA. *J. Chem. Theory Comput.*, **11**, 5723-5736.
8. Cornell, W.D., Cieplak, P., Bayly, C.I., Gould, I.R., Merz, K.M., Ferguson, D.M., Spellmeyer, D.C., Fox, T., Caldwell, J.W. and Kollman, P.A. (1996) A second generation force field for the simulation of proteins, nucleic acids, and organic molecules. *J. Am. Chem. Soc.*, **118**, 2309-2309.
9. Perez, A., Marchan, I., Svozil, D., Sponer, J., Cheatham, T.E., 3rd, Laughton, C.A. and Orozco, M. (2007) Refinement of the AMBER force field for nucleic acids: improving the description of alpha/gamma conformers. *Biophys. J.*, **92**, 3817-3829.
10. Krepl, M., Zgarbova, M., Stadlbauer, P., Otyepka, M., Banas, P., Koca, J., Cheatham, T.E., 3rd, Jurecka, P. and Sponer, J. (2012) Reference simulations of noncanonical nucleic acids with different chi variants of the AMBER force field: quadruplex DNA, quadruplex RNA and Z-DNA. *J. Chem. Theory Comput.*, **8**, 2506-2520.
11. Zgarbova, M., Luque, F.J., Sponer, J., Cheatham, T.E., 3rd, Otyepka, M. and Jurecka, P. (2013) Toward Improved Description of DNA Backbone: Revisiting Epsilon and Zeta Torsion Force Field Parameters. *J. Chem. Theory Comput.*, **9**, 2339-2354.
12. Essmann, U., Perera, L., Berkowitz, M.L., Darden, T., Lee, H. and Pedersen, L.G. (1995) A smooth particle mesh Ewald method. *J. Chem. Phys.*, **103**, 8577-8593.
13. Ryckaert, J.-P., Ciccotti, G. and Berendsen, H.J.C. (1977) Numerical integration of the cartesian equations of motion of a system with constraints: molecular dynamics of n-alkanes. *J. Comput. Phys.*, **23**, 327-341.
14. Miyamoto, S. and Kollman, P.A. (1992) Settle: An analytical version of the SHAKE and RATTLE algorithm for rigid water models. *J. Comput. Chem.*, **13**, 952-962.
15. Hopkins, C.W., Le Grand, S., Walker, R.C. and Roitberg, A.E. (2015) Long-Time-Step Molecular Dynamics through Hydrogen Mass Repartitioning. *J. Chem. Theory Comput.*, **11**, 1864-1874.
16. Salomon-Ferrer, R., Götz, A.W., Poole, D., Le Grand, S. and Walker, R.C. (2013) Routine Microsecond Molecular Dynamics Simulations with AMBER on GPUs. 2. Explicit Solvent Particle Mesh Ewald. *J. Chem. Theory Comput.*, **9**, 3878-3888.
17. Kollman, P.A., Massova, I., Reyes, C., Kuhn, B., Huo, S., Chong, L., Lee, M., Lee, T., Duan, Y., Wang, W. *et al.* (2000) Calculating structures and free energies of complex molecules: combining molecular mechanics and continuum models. *Acc. Chem. Res.*, **33**, 889-897.
18. Islam, B., Stadlbauer, P., Neidle, S., Haider, S. and Sponer, J. (2016) Can We Execute Reliable MM-PBSA Free Energy Computations of Relative Stabilities of Different Guanine Quadruplex Folds? *J. Phys. Chem. B*, **120**, 2899-2912.
19. Lim, K.W., Ng, V.C., Martin-Pintado, N., Heddi, B. and Phan, A.T. (2013) Structure of the human telomere in Na^+^ solution: an antiparallel (2+2) G-quadruplex scaffold reveals additional diversity. *Nucleic Acids Res.*, **41**, 10556-10562.
20. Lim, K.W., Amrane, S., Bouaziz, S., Xu, W., Mu, Y., Patel, D.J., Luu, K.N. and Phan, A.T. (2009) Structure of the human telomere in K^+^ solution: a stable basket-type G-quadruplex with only two G-tetrad layers. *J. Am. Chem. Soc.*, **131**, 4301-4309.
21. Zhang, Z., Dai, J., Veliath, E., Jones, R.A. and Yang, D. (2010) Structure of a two-G-tetrad intramolecular G-quadruplex formed by a variant human telomeric sequence in K^+^ solution: insights into the interconversion of human telomeric G-quadruplex structures. *Nucleic Acids Res.*, **38**, 1009-1021.
22. Phan, A.T., Kuryavyi, V., Luu, K.N. and Patel, D.J. (2007) Structure of two intramolecular G-quadruplexes formed by natural human telomere sequences in K^+^ solution. *Nucleic Acids Res.*, **35**, 6517-6525.
23. Ambrus, A., Chen, D., Dai, J., Bialis, T., Jones, R.A. and Yang, D. (2006) Human telomeric sequence forms a hybrid-type intramolecular G-quadruplex structure with mixed parallel/antiparallel strands in potassium solution. *Nucleic Acids Res.*, **34**, 2723-2735.
24. Phan, A.T., Luu, K.N. and Patel, D.J. (2006) Different loop arrangements of intramolecular human telomeric (3+1) G-quadruplexes in K^+^ solution. *Nucleic Acids Res.*, **34**, 5715-5719.
25. Stadlbauer, P., Kuhrova, P., Banas, P., Koca, J., Bussi, G., Trantirek, L., Otyepka, M. and Sponer, J. (2015) Hairpins participating in folding of human telomeric sequence quadruplexes studied by standard and T-REMD simulations. *Nucleic Acids Res.*, **43**, 9626-9644.
26. Foloppe, N., Hartmann, B., Nilsson, L. and Mackerell, A.D. (2002) Intrinsic Conformational Energetics Associated with the Glycosyl Torsion in DNA: A Quantum Mechanical Study. *Biophys. J.*, **82**, 1554-1569.
27. Islam, B., Stadlbauer, P., Krepl, M., Havrila, M., Haider, S. and Sponer, J. (2018) Structural dynamics of lateral and diagonal loops of human telomeric G-quadruplexes in extended MD simulations. *J. Chem. Theory Comput.*, **14**, 5011-5026.
28. Dai, J., Carver, M., Punchihewa, C., Jones, R.A. and Yang, D. (2007) Structure of the Hybrid-2 type intramolecular human telomeric G-quadruplex in K^+^ solution: insights into structure polymorphism of the human telomeric sequence. *Nucleic Acids Res.*, **35**, 4927-4940.
29. Largy, E., Marchand, A., Amrane, S., Gabelica, V. and Mergny, J.L. (2016) Quadruplex Turncoats: Cation-Dependent Folding and Stability of Quadruplex-DNA Double Switches. *J. Am. Chem. Soc.*, **138**, 2780-2792.
30. Dvorkin, S.A., Karsisiotis, A.I. and Webba da Silva, M. (2018) Encoding canonical DNA quadruplex structure. *Sci. Adv.*, **4**, eaat3007.
31. Genheden, S. and Ryde, U. (2015) The MM/PBSA and MM/GBSA methods to estimate ligand-binding affinities. *Expert Opin. Drug Discov.*, **10**, 449-461.
32. Cang, X., Sponer, J. and Cheatham, T.E., 3rd. (2011) Explaining the varied glycosidic conformational, G-tract length and sequence preferences for anti-parallel G-quadruplexes. *Nucleic Acids Res.*, **39**, 4499-4512.
33. Sponer, J., Mladek, A., Spackova, N., Cang, X.H., Cheatham, T.E. and Grimme, S. (2013) Relative Stability of Different DNA Guanine Quadruplex Stem Topologies Derived Using Large-Scale Quantum-Chemical Computations. *J. Am. Chem. Soc.*, **135**, 9785-9796.
34. Sponer, J. and Hobza, P. (1994) Nonplanar geometries of DNA bases. Ab initio second-order Moeller-Plesset study. *J. Phys. Chem.*, **98**, 3161-3164.
35. Hobza, P. and Sponer, J. (1999) Structure, Energetics, and Dynamics of the Nucleic Acid Base Pairs: Nonempirical Ab Initio Calculations. *Chem. Rev.*, **99**, 3247-3276.
36. Largy, E. and Mergny, J.L. (2014) Shape matters: size-exclusion HPLC for the study of nucleic acid structural polymorphism. *Nucleic Acids Res.*, **42**, e149.
37. Chen, J.L., Zhang, Y.Y., Cheng, M.P., Guo, Y.H., Sponer, J., Monchaud, D., Mergny, J.L., Ju, H.X. and Zhou, J. (2018) How Proximal Nucleobases Regulate the Catalytic Activity of G-Quadruplex/Hemin DNAzymes. *ACS Catal.*, **8**, 11352-11361.
